# The Lack of a COPII Cargo Receptor Erv14 Impacts Physiological Functions of the Vacuole in *Saccharomyces cerevisiae*

**DOI:** 10.1111/tra.70035

**Published:** 2026-04-23

**Authors:** Paul Rosas‐Santiago, Jorge Luis Ruiz Salas, Elizabeth Cordoba, Francisco Vera‐López Portillo, Prisciluis Caheri Salas‐Navarrete, Jaime Arturo Pimentel Cabrera, Alfredo Martínez, Nayeli Imelda Roldan Ramírez, Dieter Chavelas Hernández, Andrés Saralegui‐Amaro, Martha Calahorra, Hana Sychrová, Olga Zimmermannová

**Affiliations:** ^1^ Instituto de Biotecnología, Universidad Nacional Autónoma de México Cuernavaca México; ^2^ Instituto de Energías Renovables, Universidad Nacional Autónoma de México (IER‐UNAM) Temixco Mexico; ^3^ Departamento de Genética Molecular Instituto de Fisiología Celular, Universidad Nacional Autónoma de México, Circuito Exterior s/n, Ciudad Universitaria México City Mexico; ^4^ Laboratory of Membrane Transport Institute of Physiology of the Czech Academy of Sciences Prague Czech Republic

**Keywords:** Erv14 cargo receptor, intracellular pH, thermosensitivity, vacuole, Vph1, yeast

## Abstract

Erv14 is a cargo receptor of COPII vesicles, which is necessary for the efficient trafficking of various membrane proteins. In this work, we demonstrate that the deletion of the *ERV14* gene impacts various physiological functions of the vacuole. Compared to the wild‐type cells, cells lacking the *ERV14* gene exhibited higher vacuolar pH and altered vacuolar morphology with increased fragmentation. In addition, *erv14Δ* cells exhibit a thinner cell wall and an impaired process of endocytosis. We also found the importance of *ERV14* for cells to overcome environmental stresses, such as neutral external pH, increased zinc and calcium concentrations, and high temperature. Furthermore, comparing gene expression, proteome analysis, and structural modeling revealed new interactions between Erv14 and several vacuolar proteins, including subunits of V‐ATPase and other proteins involved in carbon metabolism.

## Graphical abstract

Loss of the cargo receptor Erv14 in Saccharomyces cerevisiae results in sensitivity to calcium, zinc, neutral extracellular pH, and elevated temperature. Furthermore, cells deleted in the *ERV14* exhibits vacuolar fragmentation, enlarged lipid droplets, and metabolic alterations. Ruiz Salas, J. L. (2026) https://BioRender.com/b65g523.

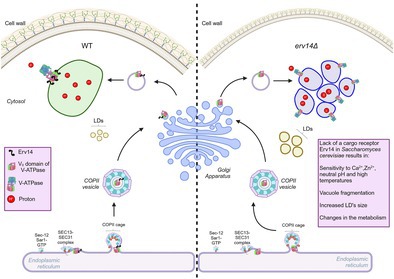

## 1 Introduction

All organisms must cope with different stresses, such as cold, heat, and starvation, to survive and complete their life cycles. For the unicellular yeast cells, a crucial intracellular organelle to survive, for example, nutrient starvation or various environmental stresses, is the vacuole [1, 2]. The vacuole plays many important roles in regulating intracellular pH and solute concentration in yeast cells. Moreover, an excess of metabolic or toxic compounds and ions accumulates in the vacuole as a response to cytoplasmic detoxification [3]. Proper vacuolar function is also necessary for cell response to osmotic shock. When cells are starving, the vacuole is crucial for the catabolism of compounds and protein turnover, and it provides part of the necessary building blocks for cellular metabolism [4, 5].

The vacuole morphology depends on nutritional cell status (i.e., glucose availability) [6, 7]. Three pathways are known to contribute to vacuole morphogenesis. Two of these pathways transport proteins from the endoplasmic reticulum (ER) to the Golgi, and the third one is the cytosol‐to‐vacuole transport (CVT), all of them finalizing their transport in the vacuole [8, 9, 10]. Vesicular trafficking is crucial for these pathways because proteins must be transported, classified, and packed into the correct vesicles to locate cellular compartments of their functions successfully. Several proteins participate in vesicular trafficking at different stages; some integrate the COPII multimeric complex. Cargo proteins are transported in COPII‐coated vesicles from the ER to the Golgi ([11], [12], [13, 14, 15, 16, 17, 18, 19]), and Erv14 is a cargo receptor of at least one‐third of cargo proteins that use the secretory pathway via COPII vesicles [20, 21, 22]. Previous studies have shown that the absence of Erv14 affects the capacity of yeast cells to survive high Na^+^ concentrations [23] or regulate plasma membrane (PM) potential, intracellular pH, and K^+^ homeostasis [24], mainly due to impairing cargo's ability to reach its destination. Erv14 belongs to the family of highly conserved cornichon cargo receptors. Animals and plants have more than one member; meanwhile, there is an *ERV14* paralogue *ERV15* is also present in the yeast [21]. Animal cornichon homologs CNIH2 and CNIH3 play two roles over AMPA‐type glutamate receptors (AMPARs) by increasing their surface expression and modulating the channel gating [25, 26]. Cornichons 1 and 4 in plants sort and activate glutamate receptor‐like channels 3.3 (GLRs) [27]. Though in yeast cells, the interaction of Erv14 with more than 40 various cargoes was characterized, its possible role as an activity modulator has not been found so far. Interestingly, in *Arabidopsis thaliana* cornichon 1 interacts with more than 500 proteins, among them, the e‐type subunit of the vacuolar V‐ATPase V_0_ integral domain [28, 29, 30].

In this study, we show that Erv14 is localized also in the vacuolar membrane where it possibly interacts with a subunit of the integral V_0_ domain of the V‐ATPase Vph1. Our AlphaFold 3 modeling shows that Erv14 may stabilize the V_0_ integral domain in the vacuolar membrane. Cells lacking the *ERV14* gene exhibit higher vacuolar pH and diminished vacuolar physiological functions, compromising cell growth under different stress conditions, such as alkaline external pH, high Ca^2+^ and Zn^2+^ concentrations, or temperatures over 28°C. Our data also show changes in genes related to vacuole homeostasis, cell wall, and lipid metabolism, evidencing a new role of Erv14 as a regulator of the V‐ATPase by its interaction with Vph1.

## 2 Results

### 2.1 Deletion of Erv14 Does Not Affect Vph1 Localization but Leads to Changes in Vacuolar pH and Plasma Membrane Potential

**FIGURE 1:**
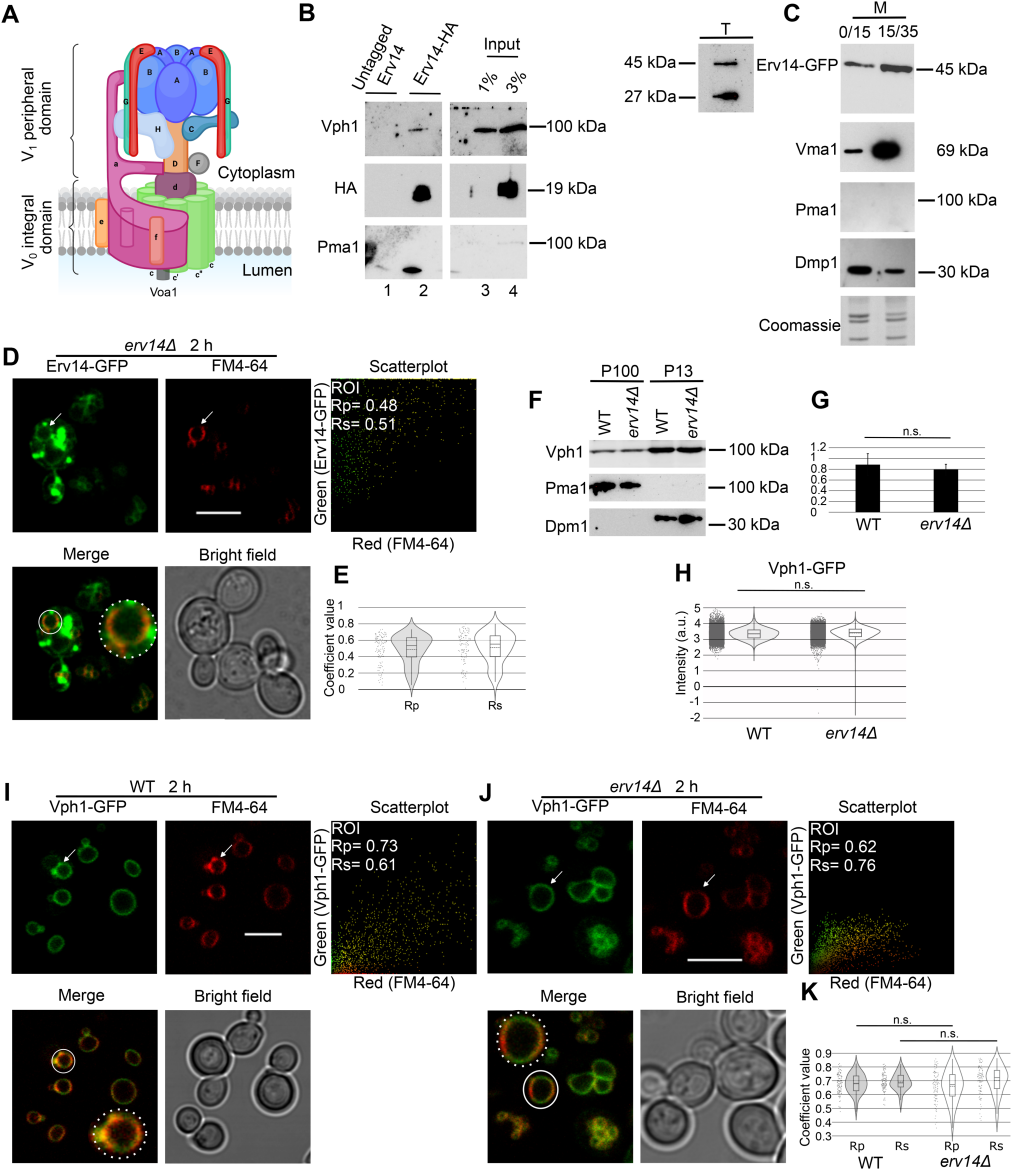
The location of Vph1 in the vacuolar membrane does not change in the absence of *ERV14*. (A) V‐ATPase model with labeled subunits. Created in BioRender. Ruiz Salas, J. (2024) https://BioRender.com/n79m870. (B) Immunoprecipitation of Erv14‐HA from BY4741*erv14Δ* cells complemented with pERV14‐HA led to the isolation of Vph1 in cells cultivated in YNB media (lane 2). Cells expressing pERV14 (untagged; lane 1) did not give a signal. Pma1 was not immunoprecipitated, although it was slightly present in the input. P100 membrane protein fraction was tested as an input (lanes 3 and 4). (C) Membrane vacuole enrichment from cells harboring Erv14‐GFP were prepared and examined by western blot analyses. Vma1, Pma1, and Dmp1 were used as markers of vacuolar membrane, plasma membrane, and endoplasmic reticulum, respectively. T, total protein extraction. M, membrane fraction isolation. (D) Confocal images of yeast cells expressing Erv14‐GFP and stained with FM4‐64 during 2 h. Colocalization analysis was performed using PSC colocalization plug‐in in Image J in a region of interest (ROI) represented with a white circle and in the insets as areas of higher magnification in the merged image. Pixel distribution of GFP (y‐axis) and FM4‐64 (x‐axis) signals are shown in the corresponding scatterplot. The obtained coefficients values of the linear Pearson (Rp) correlation coefficient and nonlinear Spearman (Rs) rank correlation coefficient. (E) Quantification of Rp and Rs coefficient values in more than 100 cells. Rp and Rs values ≥ 0.5 indicate colocalization. (F) Representative images of similar membrane distribution of Vph1 in WT and *erv14Δ* cells in low‐speed pellet (P13) and high‐speed pellet (P100) and normalized to Dpm1 (G) in three different experiments. Results were presented as bar graphs indicating standard deviation (SD) (*n* ≥ 3). Statistical analysis was performed using a paired, two‐tailed Student's *t*‐test, *p*‐values < 0.05 were considered statistically significant. (H) Mean fluorescence intensity of Vph1‐GFP from more than 41 000 WT and *erv14Δ* cells. Yeast cells were grown on YNB media using the material and methods described. n.s. stands for not significant using Mann Whitney test. Confocal images of WT (I) and *erv14Δ* (J) yeast cells expressing Vph1‐GFP and stained with FM4‐64 during 2 h. (K) Colocalization analysis as in (D) and (E). Scale bars correspond to 5 μm. White arrows mark the ROI.

The assembly of V‐ATPase is by the V_0_ integral and the V_1_ peripheral domains (Figure 1A). Vph1 is integrated into the V_0_ domain, contributing to H^+^ transport across the vacuolar membrane to reach the lumen [31, 32]. Erv14 interacts with various cargo proteins, including the Vph1 subunit [20, 33]. We corroborate this with coimmunoprecipitation (Figure 1B; complete image in Supplementary Figure S1) and immunoprecipitation assays (Supplemental Table S1).

**FIGURE 2:**
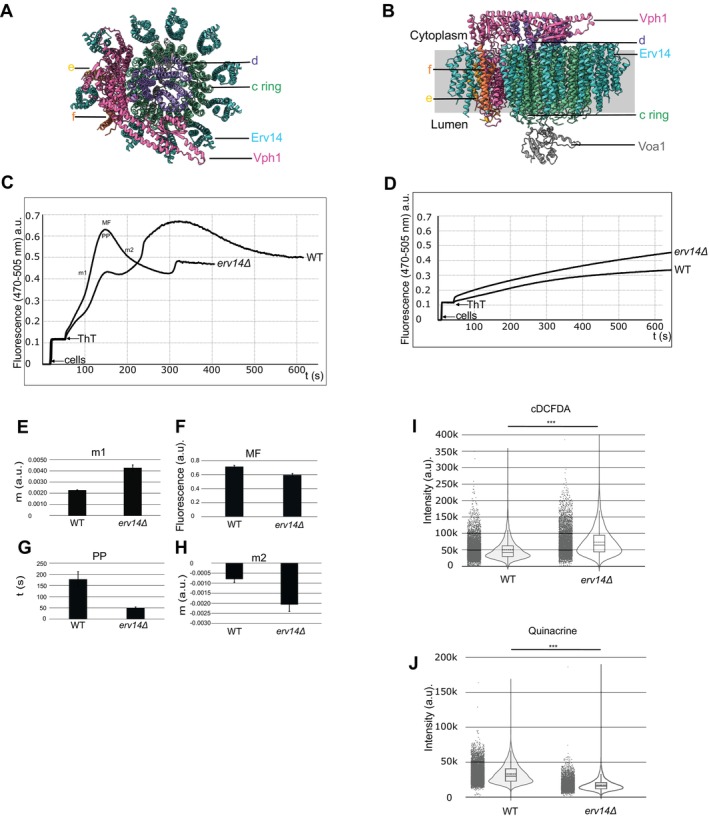
*ERV14* mutation induces changes in the plasma membrane potential and in the vacuolar pH. (A) Top view of the illustrative prediction of the V_0_ integral domain by Alphafold 3 interacting with Erv14. Colored subunits are the same as in (Figure 1A). c, c’, and c” are in green and named c ring, d is purple, Voa1 is dark gray, Vph1 is pink, e is orange, and f is ochre. Erv14 is in cyan. Structural alignment with PDB: 7FDA Structure of Reconstituted V‐ATPase, state1. (B) Side view as in (A). (C) Fluorescence changes of thioflavin (ThT) added to starved WT and *erv14Δ* cells. First, cells (50 mg, w. w.) were added to the incubation medium (10 mM MES‐TEA, 10 μM BaCl_2_ and 20 mM glucose) and after addition of 15 μM ThT traces were recorded until stabilization. Fluorescence changes were followed at 470–505 nm, excitation and emission wavelengths, respectively in an Olis‐modified SML spectrofluorometer with constant magnetic stirring and 30°C. Representative traces from three biological replicates are shown. (D) same as (C) but without glucose. m1 and m2, stands for slope; MF, maximum fluorescence; PP, plateau phase. Quantification of m1 (E), MF (F), PP (G) and m2 (H). Results were presented as bar graphs indicating standard deviation (SD) (*n* ≥ 3). Statistical analysis was performed using a paired, two‐tailed Student's *t*‐test, *p*‐values < 0.05 were considered statistically significant. Mean fluorescence intensity from cDCFDA (I) or quinacrine (J) from more than 4000 WT and *erv14Δ* cells. Yeast cells were grown on YNB media using the material and methods described. *P* value ***< 0.001 using Mann Whitney test.

Regarding the localization of Erv14 within the cell, we previously detected Erv14 in the ER and Golgi [34]. Vacuole membrane isolation by sucrose gradient showed that Erv14 is detected in this compartment (Figure 1C, Erv14‐GFP) by its increased abundant in the 15/35 fraction along with Vma1, integrating the V_1_ peripheral domains of the V‐ATPase. The plasma membrane fraction was not detected (Figure 1C, Pma1) and endoplasmic reticulum was still present in this fraction (Figure 1C, Dmp1). Figure 1C illustrates the partial cleavage of GFP from Erv14 in total protein extracts (T), with a substantial fraction of GFP remaining attached to Erv14 in both total protein extracts (T) and in the vacuolar membrane fraction (M), suggesting that Erv14 is not completely degraded at the vacuole. We also observed the localization of Erv14 in the vacuolar membrane by its colocalization with FM4‐64 (Figure 1D) with a median value of Pearson (Rp) and Spearman (Rs) coefficients of 0.53 (IQR 0.40–0.63) and 0.55 (IQR 0.40–0.65), respectively (Figure 1E). Several cargo proteins are held at the ER in *ERV14* deleted yeast cells. We observed that in the P13 fraction that corresponds to the ER [35] and partially to the vacuolar membrane [36], Vph1 (Figure 1F) is with no apparent accumulation (Figure 1G) at the ER fraction (P13) in the mutant (*erv14Δ*) compared to the wild type strain BY4741 (WT). To determine whether Vph1 abundance at the vacuolar membrane is affected by *ERV14* deletion, we employed an AMNIS imaging flow cytometer to capture single‐cell images from large populations of WT and *erv14Δ* strains and quantify total fluorescence intensity in both. The analysis revealed comparable fluorescence levels between WT and *erv14Δ* cells expressing Vph1–GFP (Figure 1H), with median values of 3.33 (IQR 3.08–3.58) for WT and 3.44 (IQR 3.18–3.67) for *erv14Δ*. Furthermore, colocalization assays between FM4‐64 and Vph1–GFP in WT (Figure 1I) and *erv14Δ* cells (Figure 1J) showed no significant differences in median correlation coefficients of Rp values of 0.68 (IQR 0.62–0.73) for WT and 0.67 (IQR 0.59–0.74) for *erv14Δ*, and Rs values of 0.68 (IQR 0.65–0.73) for WT and 0.72 (IQR 0.64–0.76) for *erv14Δ* (Figure 1K). These data indicate that the overall abundance and localization of Vph1 at the vacuolar membrane are not altered in *erv14Δ* cells. These results suggest that Erv14 is not limited in its functions as a cargo receptor of many cargoes within the ER‐Golgi membranes [20] but assigns its new role to this protein in the vacuolar membranes. To determine the role of Erv14 in the vacuolar membrane, we used *in silico* modeling (AlphaFold 3 platform [37]) to predict the possible association of Erv14 to different subunits of the V_0_ integral domain of the V‐ATPase. Interestingly, according to the model, Erv14 is predicted to interact with several subunits of the V_0_ domain, including Vph1 (Figure 2A,B). These results suggest that deletion of *ERV14* could affects subunits of the V₀ integral domain in the vacuolar membrane, which may in turn affect Pma1 activity [38, 39]. To investigate possible alterations in proton pumping activity caused by *ERV14* deletion, we employed a thioflavin T (ThT)‐based fluorescence assay using starved WT and *erv14Δ* cells prepared as described previously [40]. As shown in Figure 2C, addition of glucose to the buffer triggered a rapid increase in fluorescence intensity in both strains (compare with Figure 2D), reflecting activation of proton pumping. The fluorescence rise occurred more rapidly in *erv14Δ* cells compared to WT (Figure 2E), reaching maximum fluorescence (MF) for stabilization after 179 (SD 19) s versus 332 (SD 23) s, respectively (Figure 2F). The duration of the plateau phase (PP) was also shorter in *erv14Δ* cells 50 (SD 4.6) s compared to WT 178 (SD 33) s (Figure 2G). Subsequently, a faster decline in fluorescence was observed in *erv14Δ* cells, consistent with less stable proton pumping activity (Figure 2H). No fluorescence increase was detected in the absence of glucose (Figure 2D), confirming that the observed signal depends on glucose‐induced proton transport. Collectively, these results indicate that proton pumping activity is faster but less stable in *erv14Δ* cells than in WT, suggesting that the loss of *ERV14* alters the regulation or efficiency of proton transport, potentially affecting vacuolar function. We used two independent fluorescence probes to compare the vacuolar pH of the WT with *erv14Δ* strain. One was the pH‐sensitive fluorescein derivative cDCFDA, which increases its fluorescence intensity at higher pH. The other, the weakly basic dye quinacrine, accumulates within acidified compartments and can be observed as fluorescently labeled vacuoles [41, 42, 43, 44, 45, 46]. We used again an AMNIS imaging flow cytometer to quantify the total fluorescence detected in these two cell populations (Figure 2I,J). When cDCFDA was employed (Figure 2I), the fluorescence detected in the *erv14Δ* strain increased with a median value of 64 000 (IQR 44000‐94 000) compared to that in the WT of 42 000 (IQR 30000‐62 000). When we used quinacrine (Figure 2J), the fluorescence detected in *erv14Δ* was lower, with a median value of 15 000 (IQR 12000‐20 000) than that in the WT of 30 000 (IQR 22000‐40 000). The differences were statistically significant (Figure 2I,J). These results confirm that the luminal pH of the vacuole in *erv14Δ* cells is higher than that in WT cells and suggest that the function of the V‐ATPase, the main system maintaining the acidity of the vacuole [47], is impaired.

### 2.2 Vacuole Morphology and Endocytosis Are Abnormal in *erv14Δ* Cells

**FIGURE 3:**
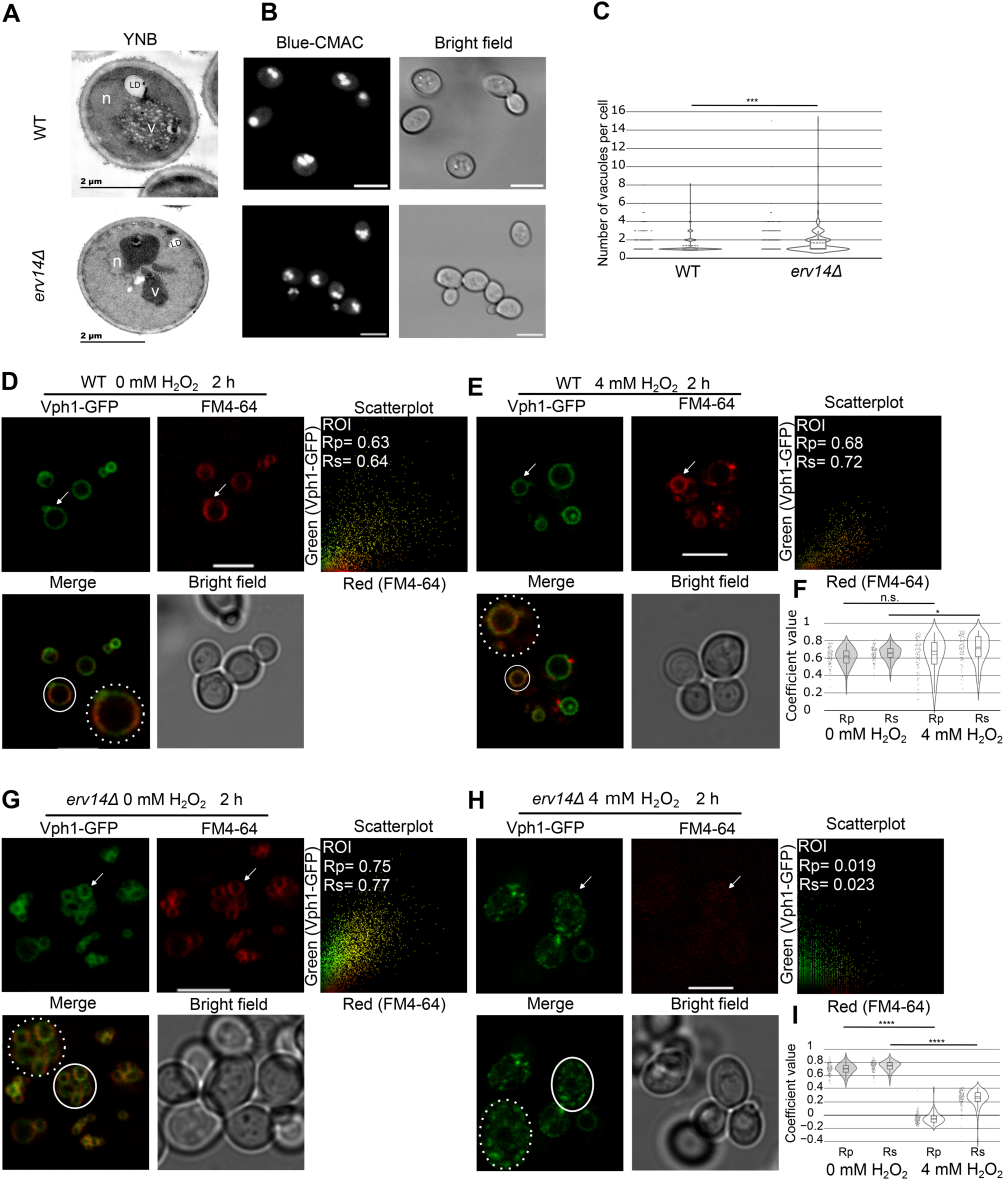
Cell morphology and endocytosis is affected in the *erv14Δ* yeast cells. (A) Cells were grown in YNB media to stationary phase, transferred to minimal media (YNB), and incubated for 12 h. Cells were then prepared for electron microscopy. Representative images of WT cells (upper panel) or *erv14Δ* cells (lower panel). n, nucleus; V, vacuole; LD, lipid droplet. Scale bar: 2 μm. (B) Cells were grown up to the exponential growth phase at 30°C. Then, they were transferred to minimal media (YNB) and incubated for 4 h. Vacuoles were stained with Blue‐CMAC (Materials and Methods) and analyzed by confocal microscopy. Images of stained WT cells (upper panel) or *erv14Δ* cells (lower panel). Scale bar: 5 μm. (C) Quantification of the number of vacuoles observed per cell in the strains imaged in B by counting Blue‐CMAC‐labeled cells (*n* > 200 cells per strain). *P* value ***< 0.001 using Mann Whitney test. WT (D) and *erv14Δ* (G) cells expressing Vph1 were stained with FM4‐64 for 2 h in 0 mM of H_2_O_2_ and then observed under confocal microscopy. WT (E) and *erv14Δ* (H) cells expressing Vph1 were stained with FM4‐64 for 2 h in 4 mM of H_2_O_2_ and then observed under confocal microscopy. Colocalization analysis was performed using PSC colocalization plug‐in in Image J in a region of interest (ROI) represented with a white circle and in the insets as areas of higher magnification in the merged image. Pixel distribution of GFP (y‐axis) and FM4‐64 (x‐axis) signals are shown in the corresponding scatterplot. The obtained coefficients values of the linear Pearson (Rp) correlation coefficient and nonlinear Spearman (Rs) rank correlation coefficient. Quantification of Rp and Rs coefficient values in more than 100 cells in WT (F) and *erv14Δ* (I) strains. Rp and Rs values ≥ 0.5 indicate colocalization. White arrows mark the ROI. Scale bars correspond to 5 μm. *P* value *< 0.01; ****< 0.0001 using Mann Whitney test.

Altered vacuolar pH can also influence vacuolar morphology [48, 49]. Therefore, we evaluated the vacuolar ultrastructure in *erv14Δ* cells using transmission electron microscopy (TEM) and compared it to the structure of vacuoles observed in the WT strain. Cells of both strains were grown in YNB media until reaching the stationary phase, transferred to fresh YNB incubated for the next 12 h (gradual glucose deprivation), and then prepared for TEM observations. Under this growth condition, WT cells showed a single vacuole (Figure 3A, YNB upper panel and Supplementary Figure S2A), as reported for WT strains in the stationary phase or with glucose deprivation [2, 50]. The *erv14Δ* strain showed fragmented vacuoles (Figure 3A, YNB lower panel and Supplementary Figure S2B). These results suggest that the vacuole response to gradual glucose starvation may depend on Erv14 function. We also examined the vacuole morphology in living cells stained with CMAC dye, which is suitable for observing vacuoles through confocal microscopy (Figure 3B). WT cells incubated in minimal media and grown at mid‐log phase contained 1–3 vacuoles per cell (Figure 3C). On the other hand, in *erv14Δ* cells, the average number of vacuoles per cell was significantly higher than in WT cells (Figure 3C). It indicates higher fragmentation of vacuoles associated with *erv14* deletion.

Vacuoles also serve for the degradation of proteins that are internalized via endocytosis from the plasma membrane [51, 52], and this process depends on the functional V‐ATPase [44, 46]. To assess if endocytosis is affected in *erv14Δ* cells, we stained yeast WT and *erv14Δ* cells with the FM4‐64 dye, which enters cells via the plasma membrane, and with prolonged incubation (up to 1 h) stains the vacuolar membrane [53]. After 2 h incubation, FM4‐64 enters the cells, stains the vacuole, and colocalizes with Vph1‐GFP at the vacuolar membrane in the WT and in the *erv14Δ* cells (Figure 3D,G and Figure 1I,J). To investigate potential endocytic defects in *erv14Δ* cells, we incubated both strains in the presence of 4 mM H_2_O_2_, a condition previously reported to delay endocytosis [54] (Figure 3E,H). In WT cells, comparison between untreated samples (0 mM H_2_O_2_; Rp = 0.64, IQR 0.56–0.70; Rs = 0.65, IQR 0.60–0.72) and cells exposed to 4 mM H_2_O_2_ (Rp = 0.67, IQR 0.53–0.77; Rs = 0.72, IQR 0.62–0.83) revealed no significant difference in the colocalization of FM4‐64 with Vph1‐GFP, as indicated by the median Rp values (Figure 3F). In contrast, *erv14Δ* cells exposed to the same oxidative stress exhibited a pronounced decrease in colocalization between FM4‐64 and Vph1‐GFP (Figure 3H). Specifically, while untreated *erv14Δ* cells displayed Rp = 0.70 (IQR 0.65–0.73) and Rs = 0.75 (IQR 0.70–0.77), treatment with 4 mM H_2_O_2_ resulted in a marked reduction (Rp = −0.06, IQR −0.10 to −0.01; Rs = 0.27, IQR 0.20–0.34) (Figure 3I). These data indicate that the lack of *ERV14* interferes with FM4‐64 endocytosis in yeast cells, as reported for some V‐ATPase mutants [46, 55].

### 2.3 Erv14 Is Necessary for Yeast Tolerance to Higher External pH, Increased Zinc and Calcium Concentrations, and High Temperatures

**FIGURE 4:**
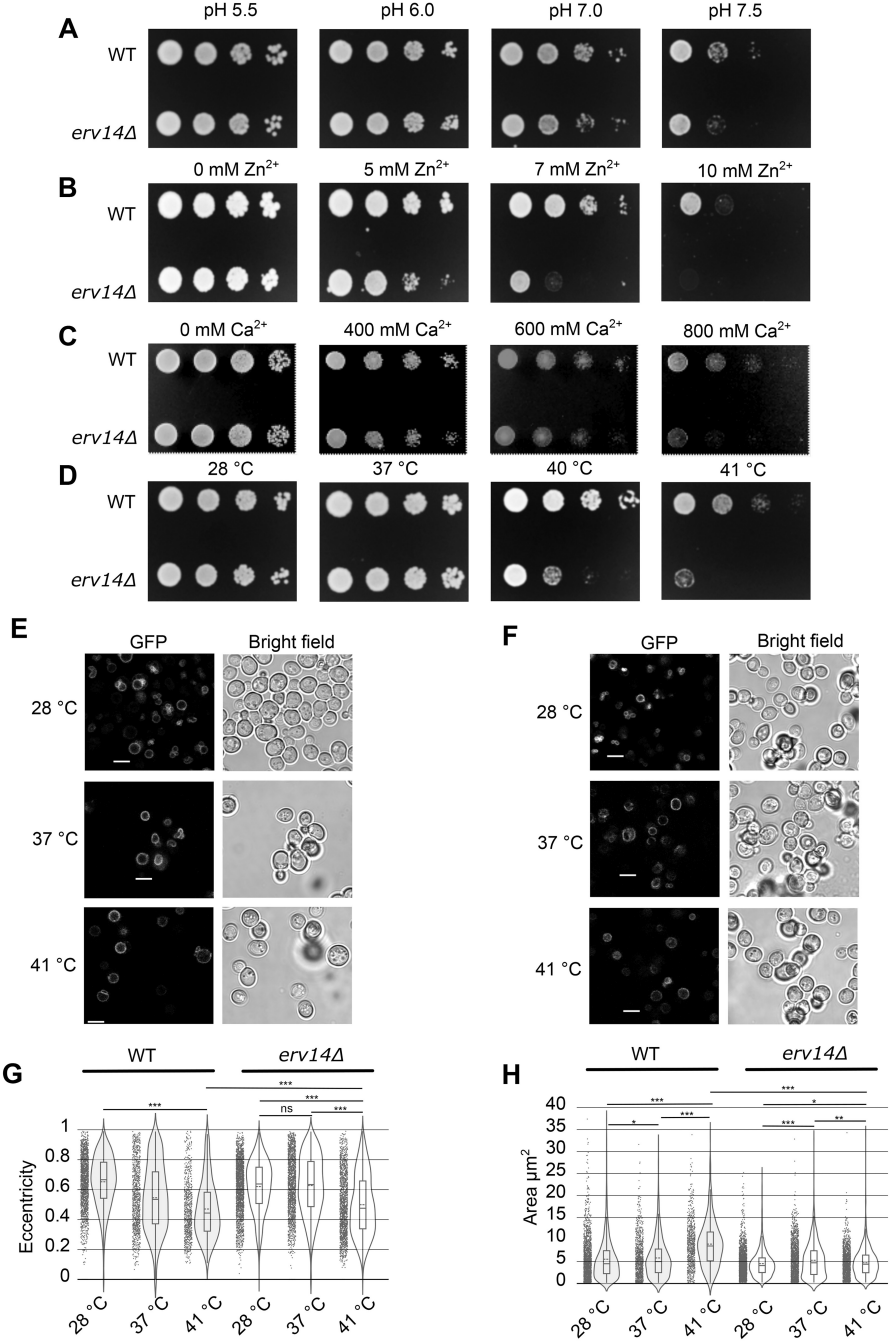
Growth defects in stressful conditions are associated with *ERV14* mutation. Growth of WT and *erv14Δ* strains on YPD media buffered in different pH levels (A), excess ZnSO_4_ (B), or CaCl_2_ (C) and incubated at 30°C for 2 days. (D) WT and *erv14Δ* were plated on YPD media and incubated at different temperatures for 2 days. pH levels, ZnSO_4_ or CaCl_2_ concentrations, and temperatures are indicated on the top in (A), (B), (C), and (D), respectively. In vivo, fluorescence micrographs of yeast show that increased temperature influences vacuolar morphology. Vph1‐GFP is monitoring at 28°C, 37°C, and 41°C in the WT (E) and *erv14Δ* (F) strains. Quantitative analysis of vacuolar eccentricity (G) and area (H) of more than 700 vacuoles from WT and *erv14Δ* strains at the same temperatures described in (E) or (F). The closer the values are to 0 (zero), the more spherical the vacuoles are. Scale bar: 5 μm. *P* value *< 0.05, **< 0.01, ***< 0.001 using the Mann Whitney test.

Acidification‐defective mutants related to V‐ATPase subunits exhibit impaired growth on media at alkaline pH [44] and on media buffered with either high concentrations of calcium or zinc [41, 56]. Thus, we next tested whether the deletion of *ERV14* affects the capacity of yeast cells to grow under any of these conditions. As shown in Figure 4, *erv14Δ* cells could not withstand the alkalinization of the media compared to the WT (Figure 4A). While the WT strain could grow in the presence of Zn^2+^ up to 10 mM, the *erv14Δ* strain tolerated only up to 7 mM Zn^2+^ (Figure 4B). Similarly, we observed that *erv14Δ* yeast cells were more sensitive to high extracellular calcium concentrations than WT cells (Figure 4C). These results indicate how Erv14 exerts its activity on vacuolar functions.

Interestingly, as cells enter the stationary growth, proteins such as the subunit Vph1 of the V‐ATPase segregate into domains at room temperature [57, 58]; however, with an increase of temperature, these vacuole domain segregations decrease [59]. Moreover, these vacuolar membrane domains exhibit phase separation during these periods of high temperatures [60] where Vph1 could participate to tolerate this stress [61]. First, we tested whether the growth of *erv14Δ* cells is sensitive to temperatures above 28°C and observed that *erv14Δ* cells could not grow as well as the WT cells at temperatures above 40°C; this phenotype was more apparent at 41°C (Figure 4D). Even though we observed Vph1 localization in the vacuolar membrane in *ERV14* deleted cells growing at 28°C (Figure 1I,J), it is reported that Vph1 needs to be in the vacuolar membrane and be functional to acidify the vacuolar lumen [62] and permit for example, the vacuolar fusion [48, 49] during logarithmic growth [59]. We decided to use Vph1 as a marker of vacuolar morphology and test the impact of the *ERV14* mutation in this process. First, we probed that Vph1 fused to GFP is functional (Supplementary Figure S3B). Vacuoles of yeast cells from both strains were imaged and analyzed during the logarithmic growth phase to determine whether a relationship between vacuole morphology and an increase in temperature exists (Figure 4E,F). First, vacuoles from WT cells harboring the Vph1‐GFP reporter were analyzed (Figure 4G–WT). The results showed that the WT vacuoles responded to temperature changes by increasing the abundance of round vacuoles (eccentricity, the closer to zero, the rounder) at 41°C. At 37°C, there were two vacuole populations, one circular and one elliptically shaped. The WT strain's most significant proportion of elliptical vacuoles was at 28°C (Figure 4G–WT). On the other hand, in cells of the *erv14Δ* strain, elliptically shaped vacuoles compromised most of the vacuole population at 28°C and 37°C, with no statistical significance between these two temperatures and more circularly shaped vacuoles were found only at 41°C (Figure 4G‐*erv14Δ*). However, the *erv14Δ* vacuole mean eccentricity was still significantly higher than in the WT even at 41°C (*p* < 0.001). We also quantified an increased area for the vacuoles in response to the increment in temperature in both strains (Figure 4H). The increase of the area with higher temperature was less steep in the *erv14Δ* strain than in the WT (Figure 4H, compare WT vs. *erv14Δ*), showing larger vacuoles in cells of the WT strain than in *erv14Δ* cells (Figure 4H–WT). These results suggest that Erv14 function is necessary for yeast tolerance to high temperature, high zinc and calcium concentrations, and external neutral pH conditions through enabling vacuole morphology adjustment.

### 2.4 *ERV14* Mutation Affects the Growth Rate of Yeast Cells

**FIGURE 5:**
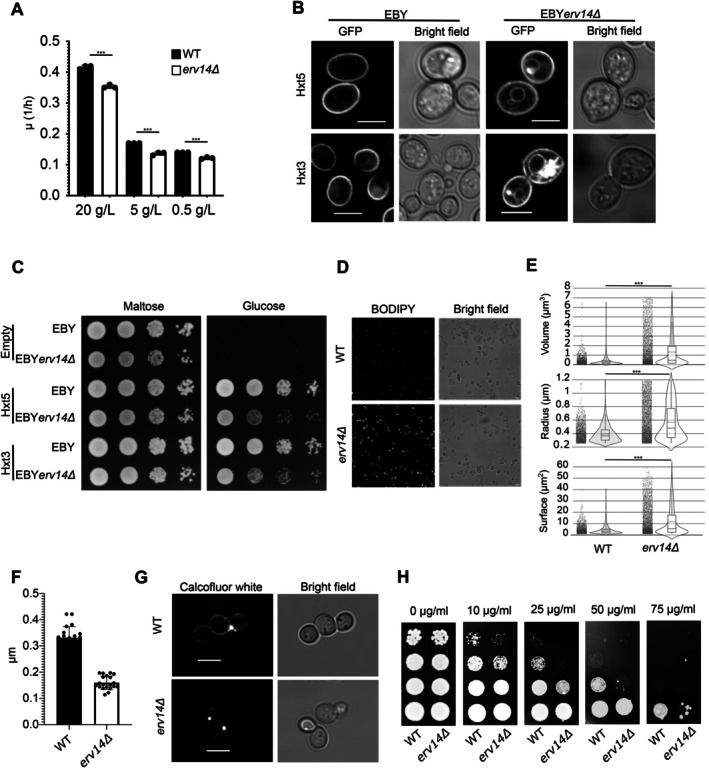
*ERV14* mutation affects the metabolism of yeast cells. (A) Growth rates of WT (solid black bars) and *erv14Δ* (empty bars) strains were calculated from the growth curves in YNB media with the different quantities of glucose. *P* values ***< 0.001. (B) Cells expressing Hxt3‐GFP and Hxt5‐GFP in EBY and EBY*erv14Δ* were visualized by fluorescent microscopy. (C) A drop test assay was conducted to evaluate glucose uptake by Hxt3 or Hxt5 in both EBY and EBY*erv14Δ* strains. (D) Live‐cell confocal imaging of yeast cells under YNB media from WT and *erv14Δ* strains and treated with BODIPY. (E) Quantitative analysis of lipid droplets volume, radius, and surface from WT and *erv14Δ* strains. More than 2000 lipid droplets were measured (see Materials and Methods). *P* value ***< 0.001 using the Mann Whitney test. Scale bar: 5 μm. (F) Cell wall measurements of images using transmission electron microscopy from WT and *erv14Δ* cells. (G) Images of yeast cells from WT and *erv14Δ* strains stained with Calcofluor white. (H) A drop test assay to evaluate the sensitivity of both strains grown on YNB media with different Calcofluor white concentrations. The scale bar represents 5 μm.

Recently, a study conducted in *S. cerevisiae* by Okreglak *et a*l. [63] found that vacuolar pH increases before cell division and diminishes as cells divide, linking the cell cycle with the vacuolar pH, regulating the cell's growth rate. We noticed that the growth rate in liquid media, even at low sugar concentrations such as 0.5 g/L, was lower in the *erv14Δ* strain (Figure 5A). To inquire more into the connection of Erv14 with the V‐ATPase and growth rate, we analyze the contribution of glucose uptake by HXT transporters due to previous reports indicating that in *S. cerevisiae*, some hexose (HXT) transporters are cargoes of Erv14 [20, 33], suggesting the relation of Erv14 and carbon metabolism. We observed the localization of two hexose transporters, Hxt3 and Hxt5, tagged with GFP expressed from multi‐copy plasmids in the EBY.VW.4000 strain, which lacks all genes encoding HXT transporters in the genome [64], or in its derivative lacking the *ERV14* gene. Hxt5 is localized at the PM in the WT (EBY) strain. In contrast, in the *erv14Δ* (EBY*erv14Δ*) strain, its localization includes both the PM and the ER (Figure 5B). We observed the same localization for Hxt3 (Figure 5B). Due to the partial stacking of Hxt3 or Hxt5 in the ER in cells without Erv14, we observed that the growth rate of EBY*erv14Δ* cells expressing Hxt3 or Hxt5 was slightly reduced in comparison with EBY cells on solid media (Figure 5C). To find out the general effect of *ERV14* deletion on carbon metabolism, we compared the rates of glucose consumption and the production of ethanol, glycerol, pyruvate, and acetic acid compounds between the WT (BY4741) and BY4741*erv14Δ* cells (Table 1). Though there was no significant difference in consumption of glucose, the specific rate of ethanol, glycerol, and pyruvate production was significantly lower in the *erv14Δ* strain (Table 1). Which indicates a general metabolic change in the *ERV14* deleted cells perhaps because vacuolar pH oscillation during cell cycle is not adjusted as in the WT cells [65].

**TABLE 1:** Specific rates (q) calculated for the growth of BY4741 and *BY4741erv14Δ* cultivated in minimal medium, pH 5.5 and 30°C.

Strain	μ (1/h)*	Specific rates (mmol/gDCW/h)				
		qGlc	qEtOH*	qGly*	qPyr*	qAceAc
BY4741	0.25 ± 0.005	22.88 ± 1.72	42.57 ± 2.16	4.58 ± 0.18	0.16 ± 0.01	1.00 ± 0.11
BY4741*erv14Δ*	0.20 ± 0.017	21.87 ± 4.81	29.93 ± 2.00	3.55 ± 0.19	0.09 ± 0.01	1.24 ± 0.39

Abbreviations: AceAc, acetic acid; DCW, dry cell weight; EtOH, ethanol; Glc, glucose; Gly, glycerol; Pyr, pyruvate.

* Among BY4741 and BY4741*erv14Δ* strains, the significant differences were found at *p* < 0.05.

How carbon metabolism is related to lipogenesis is an interesting field to the biofuel producers [66]. Because we observed different effects on the vacuole due to *ERV14* mutation (Figures 1, 2, 3, 4), we thought that, as previously stated, mutations in V‐ATPase are related to the correct LD (lipid droplet) assembly, maintenance or degradation [67, 68, 69]. To analyze the LD formation in WT and *erv14Δ* strains, cells grown in YNB media to the stationary growth phase were stained with BODIPY (Figure 5D) and the volume, surface, and radius of LD visualized by confocal microscopy were analyzed (Figure 5E). These measurements confirmed that the *erv14Δ* cells contain larger LDs than the WT (Figure 5E). These data evidence the impact on LDs in the *erv14Δ* strain probably due to change in V‐ATPase function.

Recently, it was shown in *Aspergillus fumigatus* that the synthesis of cell wall polymers is related to carbon metabolism [70]. We noticed that the cell wall of the WT yeast cells was thicker than that of the *erv14Δ* cells (Figure 3A). Therefore, we quantified the thickness of the cell wall in WT and *erv14Δ* cells. The measurements corroborate our observations that the cell walls from WT cells were thicker than those of the *erv14Δ* cells (Figure 5F). The staining of the cell wall of WT and *erv14Δ* strains with Calcofluor white (Figure 5G) also showed that the cell wall of *erv14Δ* cells was hardly observable compared to the WT. Moreover, while increasing Calcofluor white concentrations inhibits the growth of both WT and *erv14Δ* strains, the drop test in Figure 5H shows that *erv14Δ* cells were more sensitive to Calcofluor white than WT. This result suggests that the changes in the carbon metabolism by the *ERV14* mutation also impact the cell wall integrity.

### 2.5 Microarray Analysis Evidences a Different Regulation of Genes Related to Vacuoles and LDs in the *erv14Δ* Strain

**FIGURE 6:**
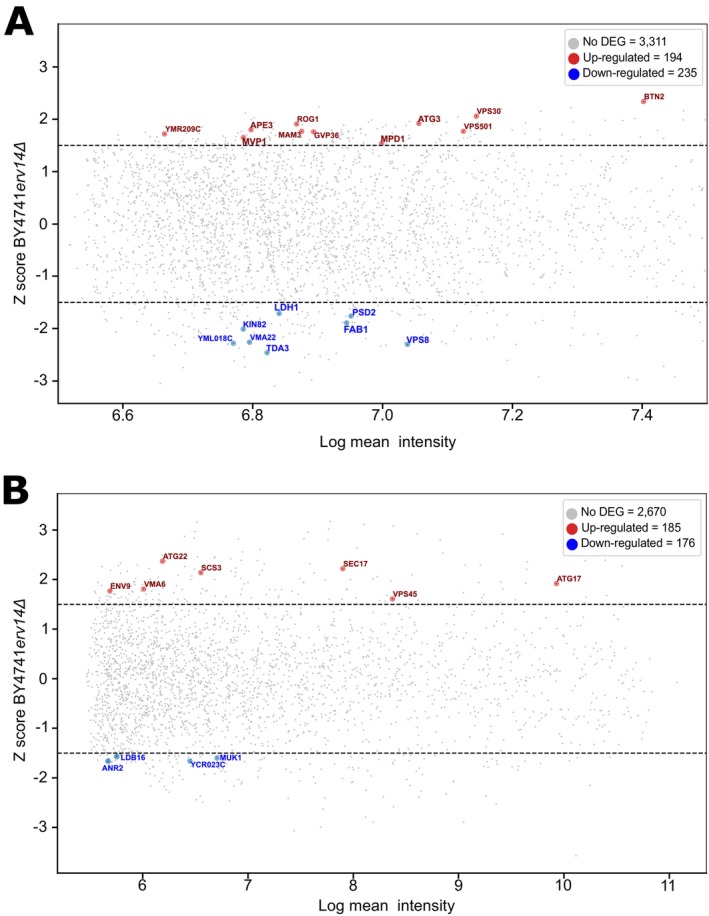
MA plot of DEGs in *erv14Δ* based on the absolute Z‐score value of −1.5 to 1.5. Highlighted genes involved vacuole fusion, autophagy, lipid droplet biogenesis, and hexose transport. Genes are up (red) and down (blue) regulated in *erv14Δ* after 16 h post inoculation (A) and after 3 days post inoculation (B).

Our observations described above (Figure 2) evidenced that the loss of function of Erv14 perturbs endocytosis, vacuole morphology, and the cell tolerance to some stress related to the normal function of the vacuole (Figures 3, 4 and 4). To inquire into the molecular processes in which Erv14 participates, we conducted a microarray analysis comparing the *erv14Δ* and WT strains. WT and *erv14Δ* strains were grown in YNB media and cells were collected under two conditions: 16 h post‐inoculation (16‐hpi) and three days post‐inoculation (3‐dpi) to evaluate the changes in gene expression triggered by *ERV14* deletion. These analyses selected a Z‐score value from −1.5 to 1.5 as a threshold to identify differentially expressed genes (DEGs). In the 16‐hpi assay, we observed an upregulation of 194 genes, and 235 genes were downregulated (Figure 6A), whereas, for the 3‐dpi condition, we recovered 361 DEGs, of which 185 were upregulated, and 176 were downregulated (Figure 6B). We focused mainly on genes related to the structure and function of the vacuole, and the phenotypes observed in the *erv14Δ* strain (Figures 2, 3, 4). Under 16‐hpi condition, one of the genes that increased its expression the most in *erv14Δ* was *BTN2* (Figure 6A). *BTN2*'s overexpression decreases arginine uptake in the vacuole, thus affecting its homeostasis [71, 72]. Conversely, we observed a decrease in expression of *VMA22*, product of which is necessary for the assembly of V‐ATPase [73, 74], and *FAB1* and *VPS8*, both important for vacuole structure and endocytosis, respectively [75, 76]. Interestingly, *BTN3*, a negative regulator of *BTN2* [77], was also downregulated. Under the 3‐dpi condition, the *erv14Δ* strain showed an increase of expression of *SEC17*, involved in vacuole fusion [78], as well as in *VMA6* and *VPS45*, which participate in the assembly and control of the levels of the V_0_ subunit of the V‐ATPase [79, 80, 81]. Together, these findings suggest a role of Erv14 in the adjustment of vacuolar function.

The increase observed in the size of LDs in the *erv14Δ* strain (Figure 5D,E) led us to investigate the expression of genes associated with the biogenesis/homeostasis of this organelle. We identified that under 16‐hpi, *LDH1* was downregulated. This gene's function is essential for maintaining LD structure [82], and interestingly, *LDH1* deletion produces giant lipid droplets [83]. At 3‐dpi, in turn, we found that *SCS3* and *ENV9* were upregulated; these genes are associated with the early stages of LD biogenesis and with their structure, respectively [84, 85, 86]. Furthermore, *SCS3* deletion impedes LD's liberation from the ER membrane [87]. In contrast, *LDB16*, which participates in LD's assembly and whose mutant exhibit LDs with irregular size and defective circularity [88], and *ANR2*, involved in LD metabolism, were downregulated [89, 90]. These results also confirm that Erv14 participates in the biogenesis/homeostasis of LDs.

*Saccharomyces cerevisiae* genome encodes 20 members of the HXT family [91]. The observed retention of Hxt3 and Hxt5 in *erv14Δ* cells (Figure 5B) might influence the expression of other *HXT*s as a consequence of the signal transduction pathways responsible for their expression [91, 92, 93]. In the microarrays, we identified a decrease in *HXT2* and *HXT3* under 3‐dpi but an increase in the expression of *HXT9* (Figure 6B), which could stem from the lack of Hxt3 and Hxt5 (and most probably also of Hxt2) at the PM. The complete list of genes from both microarrays is shown in Supplemental Table S2. A network genetic interaction analysis also evidenced genes involved in vacuolar morphology and homeostasis (Supplementary Figure S4A,B).

## 3 Discussion

Our data in *S. cerevisiae* (Figure 1B and Table S1) corroborates the interaction of Erv14 with Vph1, a subunit of the V_0_ domain of V‐ATPase [20, 33]. In *A. thaliana*, we reported that *At*CNIH1 interacts with *At*VHA‐e1, a subunit of the V_0_ domain [29]. Moreover, we observed that Erv14 was detected in the vacuoles of the yeast cells (Figure 1C,D), corroborating previous reports [33]. We also corroborate the functionality of Erv14 fused to GFP ([34]; Supplementary Figure S3A). We reported that the absence of *ERV14* retains K^+^ transporters Nha1 and Trk1 [23, 24] in the ER, nevertheless, Vph1 is still in the vacuolar membrane in *erv14* deleted yeast cells (Figure 1I–K) and is not held in the ER (Figure 1F,G) nor less present in the vacuole (Figure 1H). Vph1 is part of the V_0_, the integral proton‐translocating complex of V‐ATPase [94, 95] and interacts with Erv14 (Figure 1B; [20, 33]) in the vacuole (Figure 1B–D). All our results indicate that Erv14 may function as a chaperonin component of Vph1 in the ER [96] where, several chaperones are required to assemble the V_0_ domain [73, 97, 98, 99, 100, 101, 102, 103]. It is possible that in the absence of *ERV14* (Figure 1I–K) the stabilization of Vph1 in the vacuolar membrane is decreased (predicted by a model in Figure 2A,B) and Vph1 cannot properly fulfill its function, which may affect the activity of V‐ATPase, and, consequently, result in less‐acidic vacuolar lumen (Figure 2I,J), inhibition of the vacuolar fusion (Figures 3B,C and 4G), delayed endocytosis (Figure 3F,I), increased sensitivity to alkaline pH, high concentrations of calcium and, zinc ions and elevated temperatures (Figure 4A–D) and alters the proton pumping (Figure 2C,D). Moreover, Erv14 associates to some components of COPII vesicles [22, 33, 104] and at the same time interacts with the cargo proteins [33, 34]. As a proposal, it could be possible that Erv14 is doing something similar to the complex CNIH2/3‐AMPAR [26, 105] and regulates V‐ATPase activity not only because of its interaction with Vph1 but also by its interaction with V_0_ subunits (Figure 2A,B; Supplementary Figure S5A,B). Further studies will be necessary to test this hypothesis. Recent studies by GFP‐Trap pull‐down showed that Vph1 interacts with Rtc5 to promote the disassembly of the vacuolar V‐ATPase [106]. Nevertheless, Rtc5 was not detected by other studies such as cryo‐EM of V‐ATPase where other proteins like Vma12, 21, and 22 were detected as important for the regulation of assembly of V‐ATPase [74], opening the possibility of multiples ways of regulation of V‐ATPase complex as proposed by Erv14 (Figure 2A,B). Further studies would be necessary to determine the scope of Erv14 over the function of the V‐ATPase in the V_1_ subcomplex responsible for ATP hydrolysis [107, 108, 109].

Mutations in some subunits of the V‐ATPase complex are defective in endocytosis [46, 55], exhibit ultrastructural changes in their vacuoles, precisely forming non‐fusing vacuoles [9, 75]. We observed that the deletion of the *ERV14* generated abnormal endocytosis (Figure 3F,I) and fragmented vacuoles using TEM and staining living cells with CMAC dye (Figure 3A–C). Interestingly, a genetic screen study for yeast deletion‐mutant strains using FM4‐64 showed that either the mutation of *ERV14* or *VPH1* had small defects of vacuole fragmentation, showing the same phenotype [75]. This result showed convergence of either Erv14 or Vph1 in the vacuole fusion. Additionally, we detected the interaction of Erv14 with Vtc3 and Vtc4 (Supplemental Table S1) that are important for V‐ATPase stability and vacuole fusion [110, 111], increasing the evidence of our proposed participation of Erv14 in the vacuolar function. Furthermore, the genetic network interaction models based on our microarray data evidenced several genes involved in vacuole homeostasis and morphology (Supplementary Figure S4A,B). These results may further explain why the *erv14Δ* strain exhibits endocytosis deficiencies (Figure 3D–I), as well as defects in the vacuolar lumen pH maintenance (Figure 2I,J), or the sensitivity to neutral extracellular pH, high Ca^2+^ and Zn^2+^ extracellular concentrations, and temperature over 37°C (Figure 4A–D). Likely, the more fragmented vacuoles observed in the *erv14Δ* cells fail to form a homotypic fused vacuole [112, 113, 114], disrupting the catabolic activity of this organelle under the aforementioned stresses (Figure 4A,D).

Vacuolar and plasma membrane proton pumps control the cytosolic pH [115]. We observed that *erv14Δ* cells had lower cytosolic pH [24] and higher vacuolar pH (Figure 2I,J). These results could be related to the mislocalization of the Na^+^, K^+^/H^+^ antiporter Nha1 and K^+^‐importer Trk1, which both were found to be important for the maintenance of cytosolic pH [24, 116, 117] or because of the effect of altered function of the V‐ATPase over Pma1 [38, 39]. Together with results obtained in this work, it means that Erv14 plays an important role in the regulation of cellular pH.

It was reported that an increment in temperature evokes an increase in vacuole size [43] probably by the homotypic vacuole fusion that requires the correct acidification of this organelle via V‐ATPase [62]. We confirm that an increment in temperature increased the vacuole area in the WT strain (Figure 4H) and at the same time we detected the rounder vacuole (close to zero) at 41°C (Figure 4G), showing the vacuole‐vacuole fusion under higher temperatures. In the *erv14Δ* strain, the vacuole area was smaller (Figure 4H, *erv14Δ*), and the eccentricity was higher (Figure 4G, *erv14Δ*) than that in the WT at 41°C, suggesting that an incorrect vacuole acidification in the *ERV14* mutant (Figure 2I,J) prevents the increment in the vacuole size (Figure 4H). Moreover, our microarray analysis (Figure 6) showed that *FAB1* was downregulated in the *erv14Δ* strain. Fab1 produces PI(3,5)P2 which participates in vacuole morphology and regulates the V‐ATPase activity via its interaction with Vph1 [118, 119, 120, 121, 122, 123, 124]. This temperature‐sensitive phenotype was also observed in the *VMA4* mutant strain, a subunit of the V_1_ domain [125]. Additionally, we found that Vph1 is correctly located in *erv14Δ* vacuoles (Figure 1I–K). A feasible explanation for the correct Vph1 trafficking is an alternative route of this V‐ATPase subunit through unconventional protein secretion [126]. Nevertheless, more studies are required to clarify these results.

The data described here demonstrate that the Erv14 cargo receptor protein is necessary to maintain and adjust the yeast cell. We found evidence that *ERV14* deletion generates a general imbalance in yeast homeostasis. We observed that the *erv14Δ* cells do not grow at the same rate as the WT, even though their glucose uptake was the same as that of the WT (Table 1). Our data show that hexose transporters were partially stacked at ER in *erv14Δ* yeast cells (Figure 5B). COPII vesicles are part of the conventional ER‐Golgi‐TGN‐PM protein secretion [127, 128], and it was previously reported that Erv14 is necessary to stabilize the COPII vesicles [104]. Different stresses can trigger unconventional protein secretion and make it feasible for Hxt3 and Hxt5 to bypass the conventional COPII pathway to reach the PM in the *erv14Δ* cells [129]. *HXT9* increased its expression in our microarray assay, whereas *HXT2* and *HXT3* decreased their expression by 3‐dpi. Other *HXT* transcripts did not change, including *HXT5*. This result suggested differences in terms of glucose uptake in *erv14Δ* yeast cells (Figure 6). Hxt2 and Hxt3 transporters are known to interact with Erv14 [20, 33]. Hxt3 is a low‐affinity glucose transporter, and its expression is induced in a glucose‐independent manner. Hxt2 has a biphasic uptake kinetic responsible for transporting glucose at high or low glucose concentrations, and its expression is induced under low glucose concentrations [91, 130, 131]. Hxt9 is lowly expressed, and its transcript does not fluctuate in response to glucose concentrations. Mutants in the *HXT9* confer pleiotropic drug resistance, perhaps because it transports cycloheximide, sulfomethuron methyl, and 4‐nitroquinoline‐N‐oxide [132]. Hxt9 also transports N‐acetylglucosamine [133]. Therefore, the non‐selectivity of Hxt9 makes it a candidate for transporting glucose in the *erv14Δ* strain at the same rate as that in the WT (Table 1) despite the decrease in the *HXT2* and *HXT3* transcripts (Figure 6).

Recent work reported the impact of *ERV14* deletion in *Aspergillus niger* in process related to cell wall and lipid metabolism [134] manly affecting the mycelial growth. Under cell wall stress media with Calcofluor white we observed that the *erv14Δ* strain grew weaker than the WT strain (Figure 5H). Additionally, our microarray analysis showed that *SBE22*, involved in the transport of cell wall components [135] and *YEA4*, a UDP‐GlcNac transporter required for the cell wall synthesis [136] are overexpressed in the *erv14Δ* cells. Moreover, we quantified an increase in LD size in the *erv14Δ* strain. The reduction in the specific rates of pyruvate (Table 1) could have originated from the metabolic changes to increase the LD size (Figure 5D,E; [137]). Microarray analyses showed that some genes that modified their abundance are involved in LD biosynthesis (Figure 6). LDs are produced initially by the phosphorylation of glycerol [138], which produces glycerol‐3‐phosphate (G3P) and is used to form phosphatidic acid (PA), the precursor of the main component of LDs: triacylglycerol (TGA; [139, 140]). We determined that the specific rate of glycerol production was diminished in the *erv14Δ* strain (Table 1). Said reduction could be related to the consumption of this metabolite in order to produce the LDs precursor components. It is probably as well that LD consumption was not functional in the *erv14Δ* as the WT, likely because V‐ATPase is needed for LD utilization during growing cells [69, 141] in where Vph1 is participating in the TAG lipolysis [68]. Finally, it would be interesting to probe if other cargo receptors or chaperones or chemical chaperones [115, 142, 143, 144] could reestablish the normal vesicular trafficking in the *erv14Δ* mutant.

In summary, our new data show that Erv14 is necessary for the proper functioning of the vacuole, correct vacuolar morphology, vacuolar pH homeostasis, maintaining the LDs' normal size, and efficient endocytosis. Its presence allows yeast cells to grow at neutral pH, high Ca^2+^ and Zn^2+^ concentrations, and temperatures above 37°C. Altogether, Erv14 is a crucial COPII vesicle cargo receptor with general importance for yeast physiology. Our work further advances the understanding of the conventional pathway for vesicle trafficking in eukaryotic cells.

## 4 Materials and Methods

### 4.1 Plasmid Construction

*HXT3*, *HXT5*, and *VPH1* were amplified by PCR using their corresponding oligonucleotides (Supporting Information and Methods Table S1) and inserted by homologous recombination into the multi‐copy plasmid pGRU1 [145] adding the GFP sequence at the 3′‐end of these genes, resulting in pHXT3‐GFP, pHXT5‐GFP, and pVPH1‐GFP, respectively. All constructs were verified by restriction analysis and sequencing.

### 4.2 *S. cerevisiae* Strains, Culture Media, and Cultivations

The yeast strain used through this work are as follows: BY4741 (MATa *his3Δ1 leu2Δ0 met15Δ0 ura3Δ0*, EUROSCARF); BY4741*erv14Δ* [29]; EBY.VW.4000 (*hxt1‐17Δ::loxP gal2Δ::loxP stl1Δ::loxP agt1Δ::loxP ydl247wΔ::loxP yjr160cΔ::loxP*). Additionally, EBY.VW.4000*erv14Δ* knockout strains were generated by homologous recombination using the KanMX marker gene and the Cre‐loxP system [146]. LB media was used to grow bacterial strains. Yeast cells were grown in YPD (1% yeast extract, 2% peptone, and 2% dextrose), and pH was buffered using 50 mM MES/MOPS. Minimal YNB (with 0.67% yeast nitrogen base without amino acids) with 2% glucose and appropriate auxotrophic supplements were employed as indicated throughout the manuscript, as well as YPM (1% yeast extract, 2% peptone, and 2% maltose) media. For the cell growth experiments we used transparent flat‐bottom 96‐well plates (Corning) and measured A_600_ nm in a microplate reader (ELx808, Biotek). Yeast cells were cultivated in YNB media with 20 g/L of glucose at mid‐log phase and then diluted in fresh YNB with 20, 5, and 0.5 g/L of glucose and adjusted at OD_600_ = 0.05. Subsequently, 150 μL of each cell suspension was pipetted into individual wells, with each sample being tested in triplicate. One‐way analysis of variance (ANOVA) at *p* = 0.05 was used to determine statistical differences among the evaluated factors.

### 4.3 Fluorescence of ThT

For the experiments with fluorescent probe with thioflavin the procedure was done as reported before [40]. Yeast cells were grown in YPD media for 24 h and then submitted to starvation for 24 h. In a final medium of 2 mL of 10 mM MES‐TEA (2‐(N‐morpholino)ethanesulfonic acid adjusted to pH 6.0 with triethanolamine) with 10 μM BaCl_2_ and 20 mM glucose. The addition of 50 mg (w.v) of cells and the 15 μM ThT (Biotium) was made. Fluorescence was followed at the corresponding maxima of both excitation and emission wavelengths 470–505 nm. An SLM Aminco spectrofluorometer updated by Olis with stirring and temperatures regulation at 30°C was used. Slits were fixed at 8 nm.

### 4.4 Preparation of Microsomal Membrane Fractions

The microsomal membrane fraction was prepared from yeast cell cultures according to [147] with some modifications. Yeast cells were precultured at 30°C for 1 day in YNB medium. An aliquot of this cell suspension was subcultured in 500 mL for 12 h to reach the exponential phase. Cells were collected by centrifugation at 4000 × g for 5 min. After a 20 min incubation at 30°C in 0.1 M Tris, 10 mM DTT, 0.1 M glucose at pH 9.5, cells were pelleted at 4000 × g for 5 min and treated with a digestion buffer containing 0.1 M glucose, 0.9 M sorbitol, 50 mM Tris–Mes pH 7.6, 0.043% (w/v) YNB w/o amino acids, 0.043% (w/v) drop out mix ‐His and ‐Met, 0.05% Zymolyase and 5 mM DTT for 3 h at 30°C in agitation. Spheroplasts were collected by centrifugation at 4000 × g for 10 min at 4°C and resuspended in 1.1 M Glycerol, 1.5% PVP 40000, 50 mM Tris– Mes pH 7.6, 5 mM EGTA, 0.2% BSA, 1 mM PMSF, 1 mM DTT, 0.5 mM BHT and 1 mg/L Leupeptine and then homogenized in a glass homogenizer. After centrifugation at 4000 × g for 10 min to remove cell debris, the supernatant fraction was centrifuged at 13000 × g for 15 min, obtaining the P13 fraction from the pellet. Subsequently, the supernatant was centrifuged at 100000 × g for 1 h to collect the P100 membrane fraction from the resulting pellet. Fractions P13 and P100 were resuspended in a suspension buffer (0.3 M sorbitol, 5 mM Tris–Mes, pH 7.6, 1 mM DTT, 1 mM PMSF, and 1 mg/L leupeptine) and stored at −80°C until use.

### 4.5 Immunoprecipitation

For protein immunoprecipitation, protein G Dynabeads (Invitrogen; Cat 10003D) were bound to the anti‐HA antibody (ZYMED, 32–6700, Lot 40 990 898) by adding 3 μg of anti‐HA diluted in 200 μL PBS (10 mM Na_2_HPO_4_, 1.8 mM KH_2_PO_4_, 137 mM NaCl, 2.7 mM KCl; at pH 7.4 adjusted with HCl) with Tween 20 and according to the manufacturer's instructions (Invitrogen). We immunoprecipitated the target antigen by adding 0.5 mg of the P100 microsomal membrane fraction from BY4741*erv14Δ* expressing Erv14‐HA to the Dynabeads‐Ab complex. Then, a rotating table was used to incubate overnight at 4°C. Finally, a gentle, non‐denaturing elution (50 mM Glycine pH 2.8 elution buffer) procedure was done to resuspend the Dynabeads‐Ab‐Ag complex (Invitrogen). For the protein identification by the proteomic approach, 1 mg of either P13 or P100 microsomal membrane fraction was incubated with the Dynabeads protein G (Invitrogen; Cat 10003D) bound to the antibody anti‐HA (Invitrogen, H6908‐100ul, Lot 098M4812V) by adding 6 μg of anti‐HA diluted in 200 μL PBS.

### 4.6 SDS‐PAGE and Protein Immunoblotting

Samples were prepared according to Parry et al. [148]. Protein was precipitated by diluting the samples 50‐fold in 1:1 (v/v) ethanol:acetone and incubated overnight at −30°C. Said samples were centrifuged at 13 000 x *g* for 20 min at 4°C using a 5415R centrifuge (Eppendorf). Pellets were air‐dried and resuspended with Laemmli sample buffer (2.5% SDS final concentration) and heated at 60°C for 2 min before loading onto 12.5% (w/v) linear acrylamide mini‐gels. 15 mg of protein was loaded per lane. SDS‐PAGE‐separated proteins were electrophoretically transferred onto nitrocellulose membranes (Merck Millipore) using standard methods. Following transfer, proteins were stained with Ponceau S protein stain (0.1% w/v in 1% v/v acetic acid for 30 s) to envision equal loading/transfer of proteins. Membranes were then blocked with TBS (100 mM Tris, 150 mM NaCl) containing 0.02% (w/v) Na‐azide and 5% (w/v) fat‐free milk powder for 2 h at room temperature. Blocked membranes were incubated for a minimum of 3 h at room temperature with a 1:4000 dilution of primary antibody of anti‐GFP (Sigma‐Aldrich, G1544, Lot 046M4871V) and a 1:2000 dilution of primary antibody of anti‐PMA1 (Invitrogen, MA1‐91567, Lot UC2737883E) followed by the addition of a 1:2000 dilution of secondary antibodies (anti‐goat, rabbit or mouse) conjugated to horse radish (*Armoracia lapathifolia*) peroxidase (Santa Cruz Biotechnology). Immunodetection was carried out using the chemiluminescent LuminataTM Crescendo procedure (Merck Millipore). Images were captured using Gel DOCTM XR + SYSTEM (BIORAD).

### 4.7 Purification of the Vacuolar Membrane by Sucrose Discontinuous Gradient

The microsomal membrane fraction was prepared from yeast according to Nakanishi et al. [147] with some modifications. Yeast cells were precultured at 30°C for 1 day in YNB medium plus uracil, methionine, histidine, and leucine at 20 μg/mL. An aliquot of this cell suspension was cultured in 500 mL of YNB plus amino acids previously described for 12 h to reach the exponential phase. Cells were collected by centrifugation at 4000 *x g* for 5 min and resuspended in 0.1 M Tris, 10 mM DTT, 0.1 M glucose at pH 9.5 and incubated again for 20 min at 30°C. Cells were pelleted at 4000 x g for 5 min and treated with a digested buffer containing 0.1 M glucose, 0.9 M sorbitol, 50 mM Tris‐Mes pH 7.6, 0.043% (w/v) YNB w/o aa, 0.043% (w/v) drop out mix‐His and ‐Met, 0.05% Zymolase, and 5 mM DTT for 3 h at 30°C in a shaker. Spheroplasts were collected by centrifugation at 4000 x g for 10 min at 4°C and resuspended in 1.1 M Glycerol, 1.5% PVP 40 000, 50 mM Tris‐Mes pH 7.6, 5 mM EGTA, 0.2% BSA, 1 mM PMSF, 1 mM DTT, 0.5 mM BHT, and 1 mgL‐1 leupeptine and homogenized in a glass homogenizer. After centrifugation at 4000 *x g* for 10 min to remove cell debris, the supernatant fraction was ultracentrifuged at 100000 x g for 1 h, and the membranes collected from the pellet were diluted in suspension medium (consisting of 0.3 M sorbitol, 6 mM Tris‐Mes pH 7.6, and 2 mM DTT, 1 mM PMSF, and 1 mgL‐1 leupeptine) and named microsomes. The microsomal suspension was layered onto a discontinuous Suc gradient starting at the top layer of 9 mL of 15% (w/v) Suc over 9 mL of 35% (w/v) Suc and a cushion of 9 mL of 38% (w/v) Suc. Gradients were centrifuged at 100000 x g for 3 h at 4°C using a SW28 swinging‐bucket rotor in a Beckman XPN‐100 ultracentrifuge. On a discontinuous Suc gradient, the vacuolar membrane separates at the 0% to 15% Suc interface. Bands from the discontinuous gradient were collected, diluted in suspension medium, and ultracentrifuged at 100000 x g for 1 h. Resuspended pellets were collected, frozen in liquid nitrogen, and stored at −80°C.

### 4.8 Structure Prediction

The interaction of the V_0_ integral domain of the V‐ATPase with Erv14 from *S. cerevisiae* was initially predicted using the ColabFold notebook, based on AlphaFold Multimer, an advanced version of AlphaFold used for predicting protein–protein interactions and multimeric protein structures. However, due to the limitations in the number of input sequences in this version, AlphaFold 3 was subsequently utilized. AlphaFold 3, with its enhanced capacity for handling complex multimeric assemblies, enabled the inclusion of all subunits that make up the V_0_ integral domain and several Erv14. The sequences of the ATPase and the membrane proteins were retrieved from the UniProt database (accession numbers: cii P23968, ci P32842, c P25515, d P32366, V0a P53262, a P32563, e Q3E7B6, f P0C5R9, and Erv14 P53173). Default parameters and multiple sequence alignments were generated internally by AlphaFold's deep learning algorithm. The best‐ranked model was selected based on the predicted Local Distance Difference Test (pLDDT) score and predicted aligned error (PAE) matrix. The predicted models were analyzed and visualized using UCSF ChimeraX (version 1.8). Each chain was colored distinctly to facilitate the identification of interchain interactions. To identify key amino acid interactions within the predicted structure, molecular visualization and analysis were performed using ChimeraX. The protein complex was loaded into the software, and intermolecular contacts were evaluated using a distance‐based selection method. The residues involved in the interactions were determined using a cutoff distance of 3.0 Å for hydrogen bonds, salt bridges, as well as for hydrophobic interactions. To further validate the identified interacting residues, the model was uploaded to the PDBsum online platform. PDBsum's interaction analysis tools were used to corroborate the presence of hydrogen bonds, non‐bonded contacts, salt bridges, and disulphide bonds. The results from PDBsum were compared with the ChimeraX findings to ensure consistency in the identification of key amino acid contacts.

Hydrogen bonds and non‐bonded contacts are calculated by HBPLUS. Details of its calculations are given in:

https://www.ebi.ac.uk/thornton‐srv/software/HBPLUS/manual.html

Also shown are salt bridges, computed using the definition by Kumar and Nussinov [149].

Interface areas are computed using a program called NACCESS:

http://wolf.bms.umist.ac.uk/naccess

### 4.9 Protein Precipitation by Trichloroacetic Acid and Acetone

Protein precipitation from Immunoprecipitation assays (two independent replicates for each strain) was done as described in [150] by adding 2% (w/v) TE buffer pH 8.0, 0.06% (w/v) sodium deoxycholate, 15% (w/v) trichloroacetic acid followed by 2 h incubation on ice. Subsequently, the samples were centrifuged at 14000 x g for 20 min at 4°C. The supernatant was discarded, and the pellet was resuspended in 90% (v/v) acetone and incubated at −30°C overnight. Afterwards, membrane fractions were centrifuged at 14 000 x g for 20 min at 4°C. The supernatant was removed, and the resulting pellet was vacuum dried in a SpeedVac Concentrator (Savant, DNA120, Thermo Sci., Mexico) for 20 min.

### 4.10 Protein Identification by Tandem Mass Spectrometry (LC–MS/MS)

All samples were sent to the Proteomics Facility at the Institut de Recherches Cliniques de Montreal, Canada, and processed as previously described [151]. MS/MS samples were analyzed using Mascot (Matrix Science, London, UK; version 2.5.1). Mascot was set up to search the NCBI_S._cerevisiae_txid_4932 database (unknown version, 134 718 entries), assuming peptide digestion by trypsin. Mascot was set to screen with a fragment ion mass tolerance of 0.52 Da and a parent ion tolerance of 10.0 PPM. O + 18 of pyrrolysine and carbamidomethyl of cysteine were specified in Mascot as fixed modifications. Oxidation of methionine was specified in Mascot as another variable modification. Scaffold (version Scaffold_4.2.1, Proteome Software Inc., Portland, OR) was used to validate MS/MS based peptide and protein identifications. Peptide identifications were accepted if they could be established at a greater than 95.0% probability by the Peptide Prophet algorithm [152] with Scaffold delta‐mass correction. Protein identifications were accepted when found at a greater than 99.0% probability and with at least 2 identified peptides. Protein probabilities were assigned by the Protein Prophet algorithm [153]. The subcellular location was verified manually through the SGD project [154].

### 4.11 Confocal Microscopy

To observe the subcellular localization of the GFP‐tagged proteins, cells in exponential growth phase cultured in the specified growth media at 28°C and when necessary incubated either at 37° or 41°C during 4 h. Then, they were observed under an inverted multiphotonic confocal microscope Olympus FV1000 equipped with a 60× oil immersion objective (Olympus). GFP was visualized by excitation with a Multi‐line Argon laser at 488 nm, and with a spectral detector set between 515/30 nm for emission. Fluorescent dyes such as CellTrackerTM carboxy‐DCFDA was used in yeast cells grown at mid‐log phase, centrifuged and resuspended in 50 mM sodium citrate buffer, pH 5, containing 2% glucose to stain cells with carboxy‐DCFDA, BOBIPY (at final concentration of 1 μg/mL). FM4‐64 was used at final concentration of 4 μM. These dyes were excited by a laser at 488 nm. Cells resuspended in 10 mM HEPES buffer, pH 7.4, containing 5% glucose were stained with CMAC derivatives (at a final concentration of 100 μM), and Calcofluor white stained cells were excited with a laser at 405 nm. Cells labeled with quinacrine were treated as described by Preston et al. [155]. Cells were centrifuged and resuspended in 100 mM HEPES and quinacrine at 200 μM, pH 7.6, and incubated at 30°C for 15 min. Cells were washed twice with 100 mM HEPES and 2% glucose at pH 7.6, resuspended in the same wash buffer, and visualized by excitation laser at 488 nm.

### 4.12 Image Analysis

Colocalization quantification of images was done in a region of interest (ROI) in more than 100 yeast cells. The PSC colocalization plugin of ImageJ [156] was used to calculate Pearson and Spearman correlation coefficients [157].

### 4.13 Quantification of Fluorescence by Image‐Based Flow Cytometry

Fluorescence emitted by the pH‐sensitive probes cDCFDA and quinacrine was quantified in an Amnis imaging flow cytometer, combining the speed and statistical power of flow cytometry with the visual advantage of microscopy (Amnis, Seattle, WA, USA). Yeast cells of either WT or *erv14Δ* strains were stained either with cDCFDA or quinacrine as described before and expressed Vph1‐GFP. Yeast cell imaging acquisition was done by cell suspension aliquots of 50 μL (6 × 10^6^ cell/ml) and utilizing the image‐based flow cytometer AMNIS ImageStream Mark II at 60× magnification with low flow rate/high sensitivity setting using INSPIRE. The instrument was set as follows: Channels 01 (bright field) and 02 (green fluorescence). The objective used was a 60× NA 0.9, providing a pixel size of 0.3 mm^2^ and a laser excitation of 488 nm at 75 mW for green fluorescence. GFP, quinacrine, and cDCFDA emissions were collected on Channel 2 (480–560 nm). According to the settings of the INSPIRE software, yeast cell image acquisition was carried out with independent single cells in focus, and a threshold was adjusted for the bright field channel. These parameters allowed us to analyze around 4000 cells for each strain. The fluorescence of each single yeast cell was analyzed by a pixel mask generated by IDEAS software and cells were selected by the emission of the dyes' fluorescence collected by the specified channels (Amnis, Seattle, WA, USA).

### 4.14 Transmission Electron Microscopy

Yeast cells were grown in YNB media to stationary phase and then regrown 12 h in 50 mL of YNB at 30°C, at 200 rpm. The samples were processed according to Wright [158] with some modifications. For cells prefixation 15 mL of the cultures were transferred into 15 mL of the 2X fixative solution (4% glutaraldehyde; 0.2 M PIPES, pH 6.8; 0.2 M sorbitol; 2 mM MgCl_2_ and 2 mM CaCl_2_), incubated for 5 min at room temperature, and then centrifuged at 1500 r.p.m. for 5 min. Afterwards, the resulting supernatant was discarded, and the cellular pellets resuspended in 5 mL of the 2× fixative solution and incubated overnight at 4°C. Then, the samples were centrifuged at 1500 × g and resulting supernatant was discarded. Subsequently, the samples were washed with PIPES buffer (5 mL) and then with 25 mL of water four times. The cells were pelleted by centrifugation at 1500 × g by 5 min and then fixated with a solution of 2% KMnO_4_ for 45 min as in [158]. The samples were washed with water, treated with 1% uranyl acetate for 1 h, and dehydrated for 15 min each with successive ethanol solutions (25, 50, 75, 95, and 100%), and left in propylene oxide at room temperature. Next, the samples were embedded in EPON resin, and processed with an ultramicrotome to obtain 60 nm thin sections for visualization in TEM (Carl Zeiss, LIBRA120).

## 5 Analytic Methods

Samples from cell cultures were taken every 2 h to evaluate the specific rates of growth (μ), glucose consumption, and production of ethanol, pyruvate, acetate, and glycerol. The biomass was measured immediately, and 1 mL of the samples was frozen at −20°C. Biomass was measured indirectly as Absorbance at 600 nm (A_600_); these measurements were converted to grams of cell dry weight (gCDW) using this equation: g‐CDW = 0.5 × A_600_. The extracellular metabolites were quantified by high‐performance liquid chromatography (Waters, Millipore, Milford, MA) with a HPX‐87H ion exclusion column (Bio‐Rad, Hercules, CA, USA). A solution of 3 mM H_2_SO_4_, with a flow rate of 0.4 mL/min, was used as mobile phase. The column was operated at 60°C. The compounds were detected with a differential refractive index detector (Waters, Millipore, Milford, MA) and then quantified with a calibration curve made with pure HPLC‐grade standards.

### 5.1 Lipid Droplets Volume Characterization in Yeast Cells

To measure and analyze data relative to the number and size of intracellular lipid droplets in WT and *erv14Δ* strains, the original OIB files were processed using ImageJ software ver 1.54f [159]. Each single OIB file is composed of a z‐stack of two channels: bright field and BODIPY fluorescence. The fluorescence channel threshold was set using ImageJ's maximum entropy algorithm. The resulting stack was processed using the 3D object counter plugin [160], from which the lipid droplets volume and surface were obtained. Thousands of lipid droplets were measured for each strain, resulting in a large dataset that was processed using Plotly python to obtain the graphs described in the results (Plotly Technologies Inc., 2015). Mann Whitney tests were performed using the ScyPy python library to evaluate the statistical dependence between volume, surface, and radius population in all the WT and *erv14Δ* conditions [161]. The results reject the null hypotheses in all cases (*p* = 0.00).

### 5.2 WT and *erv14Δ* Yeast Cells Morphology as a Function of Temperature Variations

3D information relative to the vacuole's size and morphology was acquired from a dataset of confocal slices. This stack was projected using the z‐project algorithm (standard deviation option) included in Fiji [156]. Once the projected images were obtained, the fluorescence yeast cells signal was segmented from the background using the Fiji Level Set algorithm provided by ImageJ plugin (https://imagej.net/Level_Sets) from where a set of individually labeled regions of interest (ROI's) were obtained. Ideally, each ROI bounds to a single cell; nevertheless, in some cases, the ROI bounds a cluster of cells with arbitrary morphology and size. To eliminate this misestimation of the morphology and size, the entire ROI collection was manually curated, and the clustered regions were eliminated. Once the collection was filtered, the morphology parameters associated with the yeast population were measured. Each population was statistically compared to each other using a set of Mann Whitney tests.

### 5.3 RNA Isolation and Microarrays Analysis

The *S. cerevisiae* BY4741 (*MATa his3Δleu2Δmet15Δura3Δ*) and BY4741*erv14Δ* (*MATa his3Δleu2Δmet15Δura3Δ;erv14Δ:KanMx*) strains were grown in 50 mL of minimal growth medium (YNB, 2% glucose) and supplied with histidine, methionine, leucine and uracil (20 μg/mL). The strains BY4741 and BY4741*erv14Δ* were incubated at 30°C and 250 rpm; then, the cells were collected at 16 h post inoculation (hpi) and at 3 days post inoculation (dpi). The RNA was extracted with YeaStar RNA Kit (Zymo Research, USA), according to the manufacturer's instructions. The integrity of the RNA was assessed by electrophoresis on a denaturing agarose gel. To estimate RNA concentrations, each sample was measured with a NanoDrop 2000 (ThermoScientific, USA). The gene expression array was manufactured by The Institute for Cell Physiology at UNAM (IFC‐UNAM). For cDNA synthesis, 30 μg of total RNA was used as a template, labeling with dUTP‐Cy3 for BY4741 and dUTP‐Cy5 for BY4741*erv14Δ*. The data were analyzed with the package genArise developed by the Computational Unit of the IFC‐UNAM [162].

### 5.4 Genetic Interaction Network

Genetic network interactions of other candidates' genes related to the homeostasis and structure of the vacuole were inferred from the list of the 429 misregulated genes found in the cells harvested 16 hpi in BY4741*erv14Δ* and the YeastMine database downloaded from https://yeastmine.yeastgenome.org/yeastmine/begin.do. The list of inferred interactions (Supporting Information and Methods Table S2) was used to build the global interaction network from the *erv14Δ*. Computational analyses were performed in Python 3.7. We used the Pandas library for data analysis. To determine the degree of centrality from the global transcription network, the Module network was employed. We used the Matplotlib and seaborn modules for graphical representations.

### 5.5 Statistical Analysis

Data represents means of three independent biological replicates ± SD. GraphPad Prism 10 software was used to determine statistically significant values (*p* < 0.05) using a two‐tailed unpaired Student's test. Additional tests for assessing statistical differences are specified, when necessary, in the figure legend.

## Author Contributions

Conceptualization: Paul Rosas‐Santiago; Methodology: Paul Rosas‐Santiago, Jorge Luis Ruiz Salas, Elizabeth Cordoba, Jaime Arturo Pimentel Cabrera, Nayeli Imelda Roldan Ramírez, Dieter Chavelas Hernández, Martha Calahorra; Formal analysis: Paul Rosas‐Santiago, Jorge Luis Ruiz Salas, Francisco Vera‐López Portillo, Prisciluis Caheri Salas‐Navarrete, Jaime Arturo Pimentel Cabrera, Alfredo Martínez, Martha Calahorra, Nayeli Imelda Roldan Ramírez, Andrés Saralegui‐Amaro; Investigation: Paul Rosas‐Santiago, Jorge Luis Ruiz Salas, Elizabeth Cordoba, Dieter Chavelas Hernández, Francisco Vera‐López Portillo, Prisciluis Caheri Salas‐Navarrete, Nayeli Imelda Roldan Ramírez, Andrés Saralegui‐Amaro, Olga Zimmermannová, Hana Sychrová; Resources: Paul Rosas‐Santiago, Alfredo Martínez, Olga Zimmermannová, Hana Sychrová; Writing – original draft: Paul Rosas‐Santiago; Writing – review and editing: Paul Rosas‐Santiago, Jorge Luis Ruiz Salas, Elizabeth Cordoba, Francisco Vera‐López Portillo, Prisciluis Caheri Salas‐Navarrete, Jaime Arturo Pimentel Cabrera, Alfredo Martínez, Dieter Chavelas Hernández, Andrés Saralegui‐Amaro, Olga Zimmermannová, Hana Sychrová; Visualization: Paul Rosas‐Santiago; Supervision: Paul Rosas‐Santiago; Project administration: Paul Rosas‐Santiago; Funding acquisition: Paul Rosas‐Santiago.

## Funding

This work was supported by Dirección General de Asuntos del Personal Académico, Universidad Nacional Autónoma de México, IA205822.

## Ethics Statement

The authors have nothing to report.

## Conflicts of Interest

The authors declare no conflicts of interest.

## Supporting information


**Supplementary Figure 1** Complete image of Figure 1. Immunoprecipitation of Erv14‐HA from BY4741*erv14Δ* cells complemented with pERV14‐HA led to the isolation of Vph1 in cells cultivated in YNB media. Cells expressing pERV14 (untagged) did not give a signal. Pma1 was not immunoprecipitated, although it was slightly present in the input. P100 membrane protein fraction was tested as an input.
Supplementary Figure 2. Cell morphology is affected in the *erv14Δ* yeast cells. Cells were grown in YNB media to stationary phase, transferred to minimal media (YNB), and incubated for 12 h. Cells were then prepared for electron microscopy. Image of WT cells (A) or *erv14Δ* cells (B). Scale bar: 2 μm.
Supplementary Figure 3. GFP‐tagged Erv14 and Vph1 remain functional. (A) Drop test under different butyric acid (BA) concentrations demonstrate the functionality of BY4741*erv14Δ* cells transformed with pERV14‐GFP vector and pGRU1‐GFP vector (empty vector) by conferring tolerance up to 10 mM BA. (B) Drop test under different zinc sulfate (Zn^2+^) concentrations demonstrate the functionality of BY4741*vph1Δ* cells transformed with pVPH1‐GFP vector and pGRU1‐GFP vector (empty vector) by conferring tolerance up to 10 mM Zn^2+^.
Supplementary Figure 4. Genetic interaction network of *erv14Δ* at 16 hpi. (A) Global genetic interaction based on the up and down‐regulation of the 429 genes in the null mutant of *ERV14*. Only green nodes have some changes in the transcription level (B) Measurement of the degree of centrality showing some genes related to the biogenesis and homeostasis of the vacuole, such as *VAM7*, *VPS41*, *FAB1*, *VAC7*, *VAC1*4 and other genes that encode for several subunits related to the V‐ATPase assembly (*VMA9*, *MA13*, *VMA1*, *VMA8*, *VMA11*, *VMA3*, *VPH1* and *VOA1*).
Supplementary Figure 5. Schematic comparison of regulation sites between CNIH2 and Erv14. (A) Structure of the AMPA receptor GluA1/A2 in complex with the accessory subunit pairs CNIH2 and TARP‐γ8 (PDB model 7OCA). The proteins forming the AMPAR complex are visualized using different colors: glutamate receptor 2 in pink, glutamate receptor 1 in green, and CNIH2 in cyan. The amino acid residues forming the interface between CNIH2 and GluA subunits are highlighted in black. Cargo binding and identification sites are highlighted in red. COPII binding sites are shown in orange. (B) Structure of the integral V0 domain of V‐ATPase with Erv14. The proteins forming the model are visualized using different colors: Vph1 in pink, the integral V0 domain in green, and Erv14 in cyan. The proposed amino acid residues forming the regulatory interface of ERV14 with Vph1 are highlighted in black. Cargo binding and identification sites are highlighted in red. COPII binding sites are shown highlighted in orange.

**Table S1: Supplemental** Proteins identified from ER fractions analized by LC–MS/MS from *S. cerevisiae* Cellular localization and biological process was obtain from SGD project [154].

**Table S2: Supplementary** Genes identified by microarray analyses from *S. cerevisiae* .

**Data S1:** Supporting Information and Methods Table 1. Oligonucleotides used for gene cloning.

**Data S2:** Supporting Information and Methods Table 2: List of interactions used to build the global interaction network of the erv14Δ strain.

## Data Availability

This study includes no data deposited in external repositories. All relevant data can be found in the manuscript and Supporting Information files.
