# Supplementary figures and images for: The Lack of a COPII Cargo Receptor Erv14 Impacts Physiological Functions of the Vacuole in Saccharomyces cerevisiae
Source: Traffic. 2026 Apr 23;27:e70035. doi: 10.1111/tra.70035 (PMC13106738; doi:10.1111/tra.70035)

S1

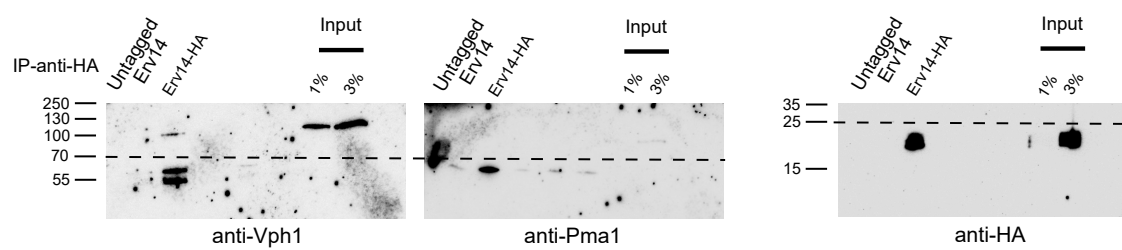

**A** WT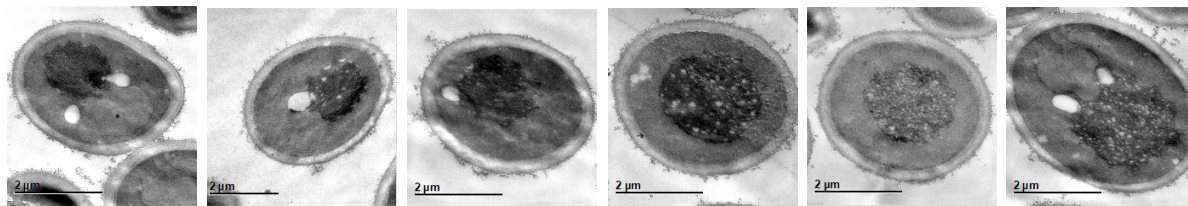**B** *erv14 $\Delta$* 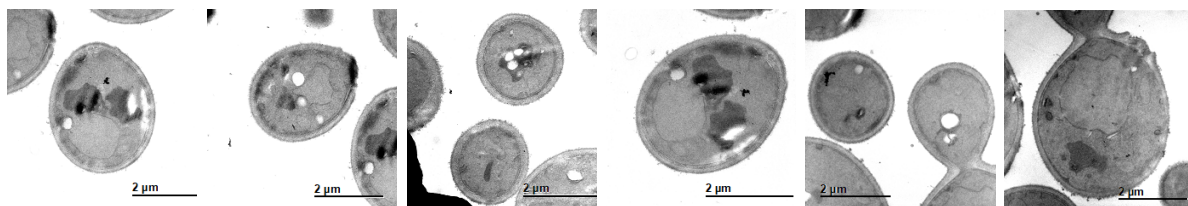

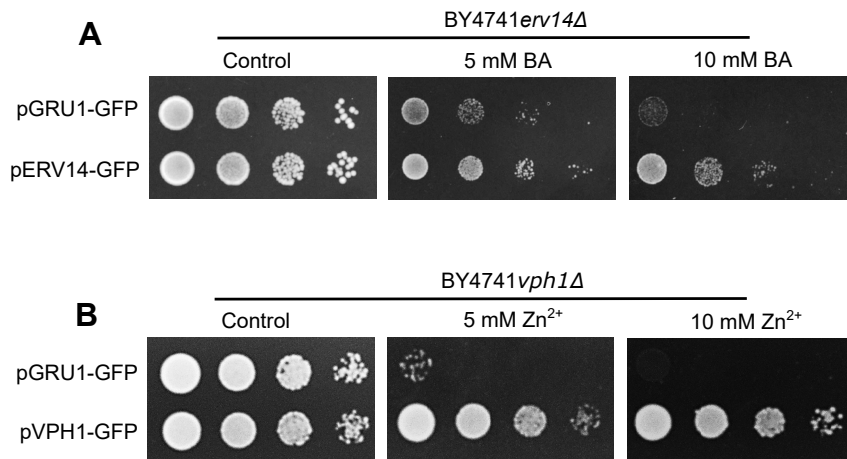

A

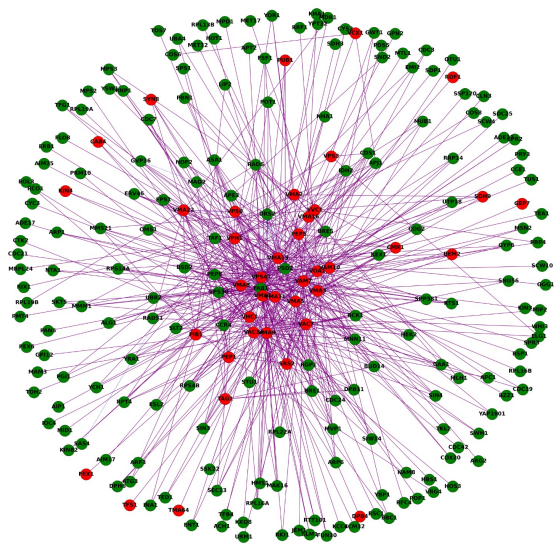

B

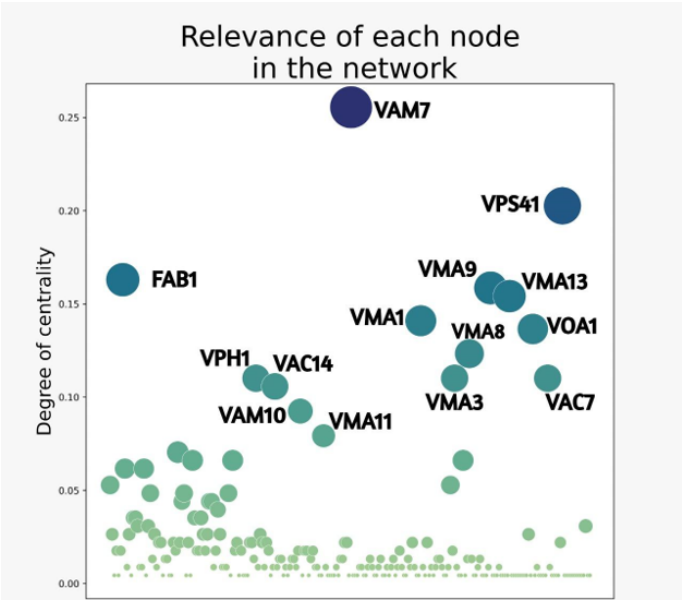

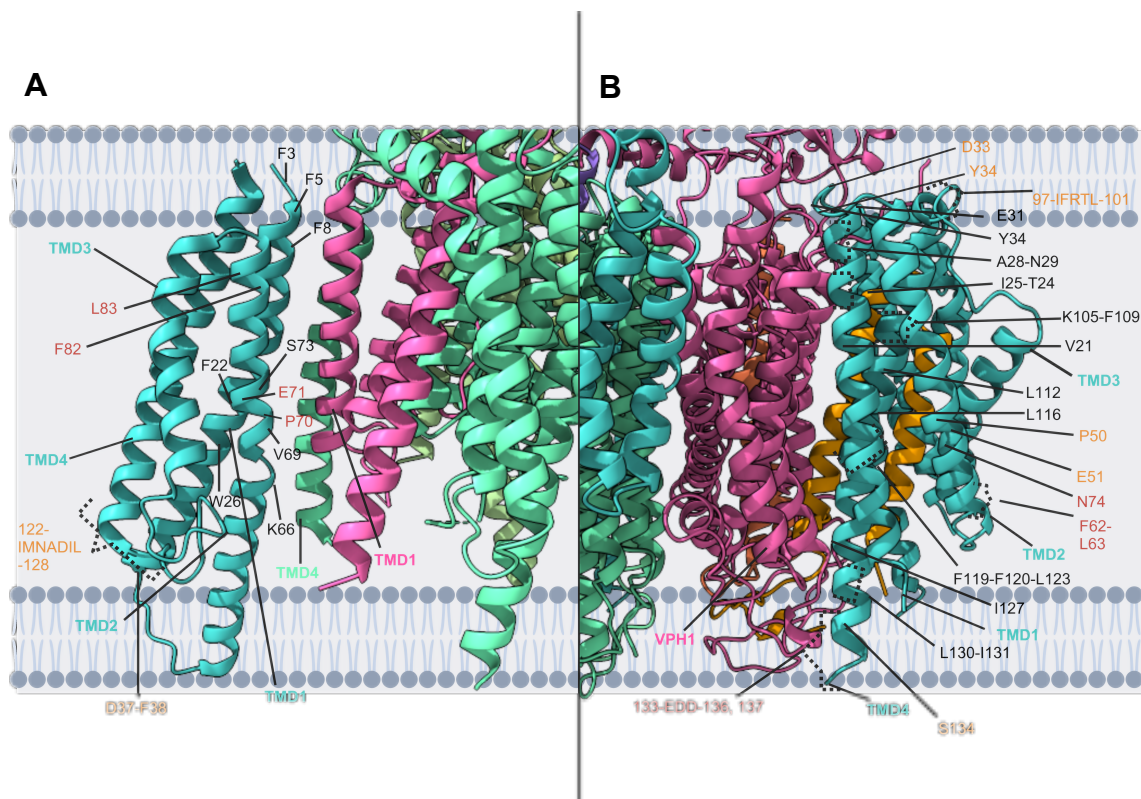

Supplement: Supplementary file 1 — Supplementary Figure 1 Complete image of Figure 1. Immunoprecipitation of Erv14‐HA from BY4741erv14Δ cells complemented with pERV14‐HA led to the isolation of Vph1 in cells cultivated in YNB media. Cells expressing pERV14 (untagged) did not give a signal. Pma1 was not immunoprecipitated, although it was slightly present in the input. P100 membrane protein fraction was tested as an input. Supplementary Figure 2. Cell morphology is affected in the erv14Δ yeast cells. Cells were grown in YNB media to stationary phase, transferred to minimal media (YNB), and incubated for 12 h. Cells were then prepared for electron microscopy. Image of WT cells (A) or erv14Δ cells (B). Scale bar: 2 μm. Supplementary Figure 3. GFP‐tagged Erv14 and Vph1 remain functional. (A) Drop test under different butyric acid (BA) concentrations demonstrate the functionality of BY4741erv14Δ cells transformed with pERV14‐GFP vector and pGRU1‐GFP vector (empty vector) by conferring tolerance up to 10 mM BA. (B) Drop test under different zinc sulfate (Zn2+) concentrations demonstrate the functionality of BY4741vph1Δ cells transformed with pVPH1‐GFP vector and pGRU1‐GFP vector (empty vector) by conferring tolerance up to 10 mM Zn2+. Supplementary Figure 4. Genetic interaction network of erv14Δ at 16 hpi. (A) Global genetic interaction based on the up and down‐regulation of the 429 genes in the null mutant of ERV14. Only green nodes have some changes in the transcription level (B) Measurement of the degree of centrality showing some genes related to the biogenesis and homeostasis of the vacuole, such as VAM7, VPS41, FAB1, VAC7, VAC14 and other genes that encode for several subunits related to the V‐ATPase assembly (VMA9, MA13, VMA1, VMA8, VMA11, VMA3, VPH1 and VOA1). Supplementary Figure 5. Schematic comparison of regulation sites between CNIH2 and Erv14. (A) Structure of the AMPA receptor GluA1/A2 in complex with the accessory subunit pairs CNIH2 and TARP‐γ8 (PDB model 7OCA). The proteins forming the AMP [file TRA-27-e70035-s003.pdf]
