# Supplementary material for: The Lack of a COPII Cargo Receptor Erv14 Impacts Physiological Functions of the Vacuole in Saccharomyces cerevisiae
Source: Traffic. 2026 Apr 23;27:e70035. doi: 10.1111/tra.70035 (PMC13106738; doi:10.1111/tra.70035)
Supplement: Supplementary file 2 — Table S1: Supplemental Proteins identified from ER fractions analized by LC–MS/MS from S. cerevisiae Cellular localization and biological process was obtain from SGD project [154]. [file TRA-27-e70035-s002.pdf]

Supplemental Table S1. Proteins identified from **ER** fractions analized by LC-MS/MS from S. cerevisiae
Cellular localization and biological process was obtain from SGD project (Wong et al., 2023)

| Protein Name | MW (kDA) | Cellular Localization | Biological Process |
|---|---|---|---|
| CDC19 | 54.54 | Plasma membrane and cytoplasm | Involved in glycolytic process |
| CHO2 | 101.21 | Endoplasmic Reticulum | Involved in phosphatidylcholine biosynthetic process |
| CWH43 | 107.86 | Plasma membrane | Involved in GPI anchor biosynthetic process |
| DIP5 | 68.11 | Vacuole | Transport |
| ELO3 | 39.47 | Endoplasmic reticulum | Involved in fatty acid biosynthetic process |
| ENA1 | 120.33 | Plasma Membrane | Transport |
| FAS1 | 228.654 | Lipid droplet and cytosol | Involved in long-chain fatty acid biosynthetic process |
| FKS1 | 214.84 | Plasma membrane | Involved in beta-glucan biosynthesis |
| GAP1 | 65.66 | Endosome, Vacuole | Transport |
| HMG1 | 115.63 | Endoplasmic Reticulum | Involved in ergosterol biosynthetic process |
| HTB1 | 14.26 | Nucleosome | Involved in chromatin organization |
| ILV2 | 74.94 | Mitochondrion | Involved in branched-chain amino acid biosynthetic process |
| IST2 | 105.87 | Plasma membrane | Involved in regulation of phosphatidylinositol dephosphorylation |
| MRH1 | 36.2 | Plasma membrane, Mitochondrion | Involved in biological process unknown |
| PDC1 | 61.48 | Cytoplasm | Involved in glycolytic fermentation to ethanol and in pyruvate metabolic process |
| PDR5 | 170.44 | Plasma membrane | Involved in intracellular monoatomic cation homeostasis |
| PHO84 | 64.372 | Plasma Membrane | Transport |
| PHO89 | 62.649 | Plasma Membrane | Transport |
| PMA1 | 99.58 | Plasma membrane | Involved in transmembrane transport |
| PMT1 | 92.66 | Endoplasmic reticulum | Involved in ERAD pathway |
| PMT2 | 86.87 | Endoplasmic reticulum | Involved in regulation of endoplasmic reticulum unfolded protein response |
| POR1 | 30.42 | Mitochondrion | Transport |
| RPS27A | 17.96 | Cytoplasm | Involved in cytoplasmic translation |
| RTN1 | 32.92 | Golgi apparatus | Involved in response to endoplasmic reticulum stress |
| SEC63 | 75.32 | Endoplasmic Reticulum | Involved in post-translational protein targeting to endoplasmic reticulum membrane |
| SPF1 | 135.25 | Endoplasmic Reticulum and cis- Golgi | Involved in intracellular divalent ion homeostasis |
| SSH1 | 53.31 | Endoplasmic reticulum | Involved in SRP-dependent cotranslational protein targeting to membrane |
| STM1 | 30 | Cytoplasm | Regulation of translational initiation in response to stress |
| STT3 | 81.53 | Endoplasmic Reticulum | Involved in protein N-linked glycosylation |
| TDH3 | 35.74 | Mitochondrion | Involved in glycolytic process |
| TEF1-TEF2 | 50.04 | Cytoplasm | Involved in tRNA export from nucleus |
| TOM40 | 42.02 | Mitochondrion | Involved in protein import into mitochondrial matrix |
| VPH1 | 95.5 | Vacuole | Involved in vacuolar acidification |
| VTC3 | 96.55 | Vacuole | Involved in vacuolar transport |
| VTC4 | 83.15 | Vacuole and endoplasmic reticulum | Involved in polyphosphate metabolic process |

Supplemental Table S1. Proteins identified from **GA** fractions analized by LC-MS/MS from S. cerevisiae
Cellular localization and biological process was obtain from SGD project (Wong et al., 2023)

| **Protein Name** | **MW (kDA)** | **Cellular Localization** | **Biological Process** |
|---|---|---|---|
| AKR1 | 85.81 | Golgi apparatus | Involved in regulation of endocytosis |
| CHO2 | 101.21 | Endoplasmic Reticulum | Involved in phosphatidylcholine biosynthetic process |
| CWH43 | 107.86 | Plasma membrane | Involved in GPI anchor biosynthetic process |
| DIP5 | 68.11 | Vacuole | Transport |
| ELO3 | 39.47 | Endoplasmic reticulum | Involved in fatty acid biosynthetic process |
| EMP70 | 75.95 | Endosome, Vacuole | Involved in endosomal and vacuolar transpor |
| ENA1 | 120.33 | Plasma Membrane | Transport |
| ERV29 | 35.02 | Endoplasmic reticulum | involved in endoplasmic reticulum to Golgi vesicle-mediated transport |
| FAS1 | 228.654 | Lipid droplet and cytosol | Involved in long-chain fatty acid biosynthetic process |
| FAS2 | 206.89 | Cytoplasm | Involved in long-chain fatty acid biosynthetic process |
| FKS1 | 214.84 | Plasma membrane | Involved in beta-glucan biosynthesis |
| GAP1 | 65.66 | Endosome, Vacuole | Transport |
| HMG1 | 115.63 | Endoplasmic Reticulum | Involved in ergosterol biosynthetic process |
| HTB1 | 14.26 | Nucleosome | Involved in chromatin organization |
| IST2 | 105.87 | Plasma membrane | Involved in regulation of phosphatidylinositol dephosphorylation |
| MRH1 | 36.2 | Plasma membrane, Mitochondrion | Involved in biological process unknown |
| PDC1 | 61.48 | Cytoplasm | Involved in glycolytic fermentation to ethanol and in pyruvate metabolic process |
| PDR5 | 170.44 | Plasma membrane | Involved in intracellular monoatomic cation homeostasis |
| PEP1 | 177.69 | Golgi apparatus | Involved in vacuolar transport |
| PHO84 | 64.372 | Plasma Membrane | Transport |
| PMA1 | 99.58 | Plasma membrane | Involved in transmembrane transport |
| PMR1 | 104.55 | Golgi membrane | Enables P-type manganese transporter activity |
| PMT1 | 92.66 | Endoplasmic reticulum | Involved in ERAD pathway |
| PMT2 | 86.87 | Endoplasmic reticulum | Involved in regulation of endoplasmic reticulum unfolded protein response |
| POR1 | 30.42 | Mitochondrion | Transport |
| RPL12A | 17.82 | Cytoplasm | Involved in cytoplasmic translation |
| RPL23B | 14.48 | Cytoplasm | Involved in cytoplasmic translation |
| RPS14A | 14.54 | Cytoplasm | Involved in cytoplasmic translation |
| RPS27A | 17.96 | Cytoplasm | Involved in cytoplasmic translation |
| RTN1 | 32.92 | Golgi apparatus | Involved in response to endoplasmic reticulum stress |
| SEC63 | 75.32 | Endoplasmic Reticulum | Involved in post-translational protein targeting to endoplasmic reticulum membrane |
| SFT2 | 24.27 | Golgi Membrane | Involved in retrograde transport, endosome to Golgi |
| SPF1 | 135.25 | Endoplasmic Reticulum and cis- Golgi | Involved in intracellular divalent ion homeostasis |
| SSH1 | 53.31 | Endoplasmic reticulum | Involved in SRP-dependent cotranslational protein targeting to membrane |
| STM1 | 30 | Cytoplasm | Regulation of translational initiation in response to stress |
| STT3 | 81.53 | Endoplasmic Reticulum | Involved in protein N-linked glycosylation |
| TCB3 | 171.07 | Endoplasmic Reticulum | Acts upstream of or within phospholipid homeostasis |
| TDH3 | 35.74 | Mitochondrion | Involved in glycolytic process |
| TEF1-TEF2 | 50.04 | Cytoplasm | Involved in tRNA export from nucleus |
| TMA7 | 6.95 | Nucleus | Involved in cytoplasmic translation |
| TY1AB | 198.66 | Cytoplasm | DNA recombination |
| VPH1 | 95.5 | Vacuole | Involved in vacuolar acidification |
| VTC3 | 96.55 | Vacuole | Involved in vacuolar transport |
| VTC4 | 83.15 | Vacuole and endoplasmic reticulum | Involved in polyphosphate metabolic process |
