# Supplementary material for: The Lack of a COPII Cargo Receptor Erv14 Impacts Physiological Functions of the Vacuole in Saccharomyces cerevisiae
Source: Traffic. 2026 Apr 23;27:e70035. doi: 10.1111/tra.70035 (PMC13106738; doi:10.1111/tra.70035)
Supplement: Supplementary file 3 — Table S2: Supplementary Genes identified by microarray analyses from S. cerevisiae . [file TRA-27-e70035-s005.pdf]

Supplemental Table S2. Gene identified of Microarray of 16 hpi from S. cerevisiae

Gene	Z-score	Function
FPS1	3.49	Aquaglyceroporin, plasma membrane channel; involved in efflux of glycerol and xylitol, and in transport of acetate, arsenite, and antimonite; key factor in maintaining redox balance by mediating passive diffusion of glycerol; phosphorylated by Hog1p and Slt2p MAPK; deletion improves xylose fermentation; regulated by Rgc1p and Ask10p, which are regulated by Hog1p phosphorylation under osmotic stress; phosphorylation by Ypk1p required to maintain an open state
Deleted	2.96	
BRE5	2.85	Ubiquitin protease cofactor; forms deubiquitination complex with Ubp3p that coregulates anterograde and retrograde transport between the ER and Golgi compartments, deubiquitinating COPII and COPI vesicle coat constituents, Sec23p and Sec27p; involved along with Ubp3p in the steady-state retention of Golgi membrane proteins, such as glycosyltransferases; null is sensitive to brefeldin A
YLR125W	2.81	Putative protein of unknown function; mutant has decreased Ty3 transposition; YLR125W is not an essential
TIM18	2.78	Component of the mitochondrial TIM22 complex; involved in insertion of polytopic proteins into the inner membrane; may mediate assembly or stability of the complex
YLR073C	2.49	Protein that inhibits Doa4p deubiquitinating activity; contributes to ubiquitin homeostasis by regulating the conversion of free ubiquitin chains to ubiquitin monomers by Doa4p; GFP-fusion protein localizes to endosomes
PRP4	2.36	Splicing factor; component of the U4/U6-U5 snRNP complex
BTN2	2.34	v-SNARE binding protein; facilitates specific protein retrieval from a late endosome to the Golgi; modulates arginine uptake, possible role in mediating pH homeostasis between the vacuole and plasma membrane H(+)-ATPase; contributes to prion curing; preferentially expressed after severe ethanol stress, involved in clearance of denatured proteins caused by severe ethanol stress
YLR077W	2.34	Protein required for assembly of respiratory complex III; mitochondrial inner membrane protein; required for an early step in assembly of respiratory complex III (cytochrome bc1 complex); mRNA is targeted to mitochondria
LOT5	2.29	Protein of unknown function; gene expression increases in cultures shifted to a lower temperature; protein abundance increases in response to DNA replication stress
AIM25	2.24	Mitochondria protein of unknown function; interacts genetically with TOR1 to regulate chronological lifespan, and the response to both heat shock and oxidative stress; involved in maintaining the integrity of the mitochondrial network; negative regulator of mitophagy flux; non-tagged protein is detected in purified mitochondria in high-throughput studies; null mutant is viable and displays an elevated frequency of mitochondrial genome loss; similar to murine NOR1
YBP1	2.22	Protein involved in cellular response to oxidative stress; required for oxidation of specific cysteine residues of transcription factor Yap1p, resulting in nuclear localization of Yap1p in response to stress; YBP1 has a paralog, YBP2, that arose from the whole genome duplication
MET17	2.21	O-acetyl homoserine-O-acetyl serine sulfhydrylase; required for methionine and cysteine biosynthesis
POP1	2.20	Subunit of RNase MRP, nuclear RNase P and telomerase complexes; RNase MRP cleaves pre-rRNA, nuclear RNase P cleaves tRNA precursors to generate mature 5' ends and facilitates turnover of nuclear RNAs, while telomerase replenishes telomeric DNA; binds to the RPR1 RNA subunit in RNase P
DSS4	2.20	Guanine nucleotide dissociation stimulator for Sec4p; functions in the post-Golgi secretory pathway; binds zinc, found both on membranes and in the cytosol
OTU1	2.16	Deubiquitylation enzyme that binds to the chaperone-ATPase Cdc48p; may contribute to regulation of protein degradation by deubiquitylating substrates that have been ubiquitylated by Ufd2p; member of the Ovarian Tumor (OTU) family; protein abundance increases in response to DNA replication stress
YAP1801	2.15	Protein of the AP180 family, involved in clathrin cage assembly; binds Pan1p and clathrin; YAP1801 has a paralog, YAP1802, that arose from the whole genome duplication
URA1	2.12	Dihydroorotate dehydrogenase; catalyzes the fourth enzymatic step in the de novo biosynthesis of pyrimidines, converting dihydroorotic acid into orotic acid
CDC42	2.07	Small rho-like GTPase; essential for establishment and maintenance of cell polarity; plays a role late in cell fusion via activation of key cell fusion regulator Fus2p; acts with ESCRT proteins at sites of nuclear envelope and ER fission; mutants have defects in the organization of actin and septins; human homolog CDC42 can complement yeast cdc42 mutant
VPS30	2.06	Subunit of phosphatidylinositol (PtdIns) 3-kinase complexes I and II; Complex I is essential in autophagy, Complex II is required for vacuolar protein sorting; required for overflow degradation of misfolded proteins when ERAD is saturated; C-terminus has novel globular fold essential for autophagy through the targeting of the PI3-kinase complex I to the pre-autophagosomal structure; ortholog of higher eukaryote gene Beclin 1; human BECN1 can complement yeast null mutant
YMR226C	2.04	NADP(+)-dependent serine dehydrogenase and carbonyl reductase; acts on serine, L-allo-threonine, and other 3-hydroxy acids; green fluorescent protein fusion protein localizes to the cytoplasm and nucleus; may interact with ribosomes, based on co-purification experiments
YRA1	2.03	Nuclear polyadenylated RNA-binding protein; required for export of poly(A)+ mRNA from the nucleus; proposed to couple mRNA export with 3' end processing via its interactions with Mex67p and Pcf11p; interacts with DBP2; inhibits the helicase activity of Dbp2; functionally redundant with Yra2p, another REF family member
YDL180W	2.02	Putative protein of unknown function; green fluorescent protein (GFP)-fusion protein localizes to the vacuole
MMO1	2.01	Protein of unknown function; SWAT-GFP, seamless-GFP and mCherry fusion proteins localize to the mitochondria; mRNA identified as translated by ribosome profiling data; MMO1 is a non-essential gene
YLR311C	2.00	Dubious open reading frame; unlikely to encode a functional protein, based on available experimental and comparative sequence data
COS6	1.99	Endosomal protein involved in turnover of plasma membrane proteins; member of the DUP380 subfamily of conserved, often subtelomeric COS genes; required for the multivesicular vesicle body sorting pathway that internalizes plasma membrane proteins for degradation; Cos proteins provide ubiquitin in trans for nonubiquitinated cargo proteins
YDL187C	1.99	Dubious open reading frame; unlikely to encode a functional protein, based on available experimental and comparative sequence data
CDC21	1.99	Thymidylate synthase; required for de novo biosynthesis of pyrimidine deoxyribonucleotides; expression is induced at G1/S; human homolog TYMSOS can complement yeast cdc21 temperature-sensitive mutant at restrictive temperature
SGF11	1.99	Integral subunit of SAGA histone acetyltransferase complex; regulates transcription of a subset of SAGA-regulated genes, required for the Ubp8p association with SAGA and for H2B deubiquitylation
LCL1	1.98	Putative protein of unknown function; deletion mutant is fluconazole resistant and has long chronological lifespan
YDR053W	1.98	Putative protein of unknown function; open reading frame overlaps 5' end of essential DBF4 gene encoding the regulatory subunit of the Cdc7p-Dbf4p kinase complex
AIM17	1.98	Protein of unknown function; the authentic, non-tagged protein is detected in highly purified mitochondria in high-throughput studies; null mutant displays reduced frequency of mitochondrial genome loss
RRP9	1.96	Protein involved in pre-rRNA processing; associated with U3 snRNP; component of small ribosomal subunit (SSU) processosome; ortholog of the human U3-55k protein
SAF1	1.95	F-Box protein involved in proteasome-dependent degradation of Aah1p; involved in proteasome-dependent degradation of Aah1p during entry of cells into quiescence; interacts with Skp1
YDR444W	1.95	Putative hydrolase acting on ester bonds
MUK1	1.95	Guanine nucleotide exchange factor (GEF); involved in vesicle-mediated vacuolar transport, including Golgi-endosome trafficking and sorting through the multivesicular body (MVB); specifically stimulates the intrinsic guanine nucleotide exchange activity of Rab family members (Vps21p/Ypt52p/Ypt53p); partially redundant with GEF VPS9; required for localization of the CORVET complex to endosomes; contains a VPS9 domain
TOS7	1.94	Protein involved in secretion and cell wall organization; localizes to plasma membrane microdomains called MCCs (membrane compartment occupied by Can1); GFP-fusion protein localizes to the cell periphery and vacuole; Sur7/PalI family transmembrane domain (TMD) containing protein; TOS7 has a paralog, DCV1, that arose from the whole genome duplication
SHG1	1.94	Subunit of the COMPASS (Set1C) complex; COMPASS methylates histone H3 on lysine 4 and is required in transcriptional silencing near telomeres
RPL39	1.94	Ribosomal 60S subunit protein L39; required for ribosome biogenesis; loss of both Rpl31p and Rpl39p confers lethality; also exhibits genetic interactions with SIS1 and PAB1; homologous to mammalian ribosomal protein L39, no bacterial homolog
YME2	1.94	Integral inner mitochondrial membrane protein; role in maintaining mitochondrial nucleoid structure and number; mutants exhibit an increased rate of mitochondrial DNA escape; shows some sequence similarity to exonucleases
ATH1	1.93	Acid trehalase required for utilization of extracellular trehalose; involved in intracellular trehalose degradation during growth recovery after saline stress
ATG3	1.92	E2-like enzyme; involved in autophagy and cytoplasm-to-vacuole targeting (Cvt) pathway; plays a role in formation of Atg8p-phosphatidylethanolamine conjugates, which are involved in membrane dynamics during autophagy and Cvt; interaction with Atg8p regulated by acetylation by NuA4 histone acetyltransferase Esa1p while attenuation of Atg3 acetylation is mediated by histone deacetylase Rpd3p; Atg12p-Atg5p conjugate enhances E2 activity of Atg3p by rearranging its catalytic site
ROG1	1.91	Lipase with specificity for monoacylglycerol; preferred substrate is 1-oleoylglycerol; null mutation affects lipid droplet morphology and overexpression causes increased accumulation of reactive oxygen species
YGR242W	1.91	Dubious open reading frame; unlikely to encode a functional protein, based on available experimental and comparative sequence data; partially overlaps the verified ORF YAP1802/YGR241C
CDC3	1.91	Component of the septin ring that is required for cytokinesis; septins are GTP-binding proteins that assemble with other septins into rod-like complexes that can associate with other rods to form filament polymers; septin rings at the mother-bud neck act as scaffolds for recruiting factors needed for cell division and as barriers to prevent diffusion of specific proteins between mother and daughter cells
YBL100W-B	1.90	Retrotransposon TYA Gag and TYB Pol genes; transcribed/translated as one unit; polyprotein is processed to make a nucleocapsid-like protein (Gag), reverse transcriptase (RT), protease (PR), and integrase (IN); similar to retroviral genes
KKQ8	1.90	Putative serine/threonine protein kinase with unknown cellular role; KKQ8 has a paralog, HAL5, that arose from the whole genome duplication
UBR2	1.89	Cytoplasmic ubiquitin-protein ligase (E3); component of the Mub1p-Ubr2p-Rad6p ubiquitin ligase complex required for the ubiquitination and degradation of Rpn4p; mediates formation of the ternary complex
DBF20	1.89	Ser/Thr kinase involved in late nuclear division; one of the mitotic exit network (MEN) proteins; necessary for the execution of cytokinesis; also plays a role in regulating the stability of SWI5 and CLB2 mRNAs; DBF20 has a paralog, DBF2, that arose from the whole genome duplication
MRPL50	1.88	Mitochondrial ribosomal protein of the large subunit; not essential for mitochondrial translation
COS8	1.88	Endosomal protein involved in turnover of plasma membrane proteins; member of the DUP380 subfamily of conserved, often subtelomeric COS genes; required for the multivesicular vesicle body sorting pathway that internalizes plasma membrane proteins for degradation; Cos proteins provide ubiquitin in trans for nonubiquitinated cargo proteins
YEL1	1.88	Guanine nucleotide exchange factor specific for Arf3p; localized to the bud neck and tip; required for localization of Arf3p to the bud neck and tip
YBL005W-A	1.86	Retrotransposon TYA Gag gene co-transcribed with TYB Pol; translated as TYA or TYA-TYB polyprotein; Gag is a nucleocapsid protein that is the structural constituent of virus-like particles (VLPs); similar to retroviral Gag
YBL100W-B	1.85	Retrotransposon TYA Gag and TYB Pol genes; transcribed/translated as one unit; polyprotein is processed to make a nucleocapsid-like protein (Gag), reverse transcriptase (RT), protease (PR), and integrase (IN); similar to retroviral genes
GPN2	1.85	Putative GTPase with a specific role in RNA pol II and polIII biogenesis; involved in the assembly and subsequent nuclear import of RNA polymerases II and III; required for establishment of sister chromatid cohesion; contains a Gly-Pro-Asn motif in the G domain; similar to Npa3p and Gpn3p; highly conserved across species and homologous to human gene GPN2/ATPBD1B
YPR064W	1.84	Putative protein of unknown function; conserved among S. cerevisiae strains; YPR064W is not an essential gene
PDR8	1.83	Transcription factor; targets include ATP-binding cassette (ABC) transporters, major facilitator superfamily transporters, and other genes involved in the pleiotropic drug resistance (PDR) phenomenon; PDR8 has a paralog, YRR1, that arose from the whole genome duplication
AAC1	1.82	Mitochondrial inner membrane ADP/ATP translocator; exchanges cytosolic ADP for mitochondrially synthesized ATP; phosphorylated; Aac1p is a minor isoform while Pet9p is the major ADP/ATP translocator; relocalizes from mitochondrion to cytoplasm upon DNA replication stress
TSL1	1.82	Large subunit of trehalose 6-phosphate synthase/phosphatase complex; Tps1p-Tps2p complex converts uridine-5'-diphosphoglucose and glucose 6-phosphate to trehalose; contributes to survival to acute lethal heat stress; mutant has aneuploidy tolerance; protein abundance increases in response to DNA replication stress; TSL1 has a paralog, TPS3, that arose from the whole genome duplication relocalizes from mitochondrion to cytoplasm upon DNA replication stress
SNT2	1.81	Subunit of Snt2C complex, RING finger ubiquitin ligase (E3); physically associates with Ecm5p and Rpd3p; along with Ecm5p, recruits Rpd3p to small number of promoters; colocalizes with Ecm5p, independently of Rpd3p, to promoters of stress response genes upon oxidative stress; involved in ubiquitination, degradation of excess histones; interacts with Ubc4p; role in regulating genes encoding amine transporters; relocalizes from nucleus to cytoplasm upon DNA replication stress
RBL2	1.81	Protein involved in microtubule morphogenesis; required for protection from excess free beta-tubulin; proposed to be involved the folding of beta-tubulin; similar to mouse beta-tubulin cofactor A; protein abundance increases in response to DNA replication stress
PDS5	1.81	Cohesion maintenance factor; involved in sister chromatid condensation and cohesion; colocalizes with cohesin on chromosomes; performs its cohesin maintenance function in pre-anaphase cells by protecting the integrity of the cohesion complex; regulates homolog pairing and facilitates synaptonemal complex formation, axis formation, interhomolog recombination and synapsis during meiosis; relocalizes to the cytosol in response to hypoxia; scaffolding protein contains 26 HEAT repeats
APE3	1.80	Vacuolar aminopeptidase Y; processed to mature form by Prb1p
OMS1	1.80	Protein integral to the mitochondrial membrane; has a conserved methyltransferase motif and is predicted to be an RNA methyltransferase; multicopy suppressor of respiratory defects caused by OXA1 mutations
YCR013C	1.80	Dubious open reading frame; unlikely to encode a functional protein, based on available experimental and comparative sequence data; transcription of both YCR013C and the overlapping essential gene PGK1 is reduced in a gcr1 null mutant
MET32	1.80	Zinc-finger DNA-binding transcription factor; involved in transcriptional regulation of the methionine biosynthetic genes; targets strong transcriptional activator Met4p to promoters of sulfur metabolic genes; feedforward loop exists in the regulation of genes controlled by Met4p and Met32p; lack of such a loop for MET31 may account for the differential actions of Met32p and Met31p; MET32 has a paralog, MET31, that arose from the whole genome duplication
RPL22A	1.79	Ribosomal 60S subunit protein L22A; required for translation of long 5' UTR of IME1 mRNA and meiotic entry; required for the oxidative stress response, pseudohyphal and invasive growth; homologous to mammalian ribosomal protein L22, no bacterial homolog; RPL22A has a paralog, RPL22B, that arose from the whole genome duplication
YER079W	1.79	Putative protein of unknown function
CDC19	1.79	Pyruvate kinase; functions as a homotetramer in glycolysis to convert phosphoenolpyruvate to pyruvate, the input for aerobic (TCA cycle) or anaerobic (glucose fermentation) respiration; regulated via allosteric activation by fructose bisphosphate; CDC19 has a paralog, PYK2, that arose from the whole genome duplication
YBL100W-B	1.79	Retrotransposon TYA Gag and TYB Pol genes; transcribed/translated as one unit; polyprotein is processed to make a nucleocapsid-like protein (Gag), reverse transcriptase (RT), protease (PR), and integrase (IN); similar to retroviral genes
FAR10	1.78	Protein involved in recovery from arrest in response to pheromone; acts in a cell cycle arrest recovery pathway independent from Far1p; interacts with Far3p, Far7p, Far8p, Far9p, and Far11p; potential Cdc28p substrate; FAR10 has a paralog, VPS64, that arose from the whole genome duplication
SAS4	1.78	Subunit of the SAS complex (Sas2p, Sas4p, Sas5p); acetylates free histones and nucleosomes and regulates transcriptional silencing; required for the HAT activity of Sas2p
YBL100W-A	1.78	Retrotransposon TYA Gag gene co-transcribed with TYB Pol; translated as TYA or TYA-TYB polyprotein; Gag is a nucleocapsid protein that is the structural constituent of virus-like particles (VLPs); similar to retroviral Gag
VPS501	1.77	Vacuolar SNX-BAR protein; cooperates with SEA complex to regulate TORC1 signaling during autophagy induction; binds phosphatidylinositol-3-phosphate; vacuolar localization of Vps501p is dependent upon direct interaction with Iml1p and a unique lipid binding specificity that is also required for its function; VPS501 has paralog VPS5 that arose from the whole genome duplication
MAM3	1.77	Protein involved in magnesium homeostasis; required for Mg2+ sequestration in vacuole; required for normal mitochondrial morphology; has similarity to hemolysins
ARP6	1.77	Actin-related protein that binds nucleosomes; a component of the SWR1 complex, which exchanges histone variant H2AZ (Htz1p) for chromatin-bound histone H2A
CYC2	1.77	Mitochondrial peripheral inner membrane protein; contains a FAD cofactor in a domain exposed in the intermembrane space; exhibits redox activity in vitro; likely participates in ligation of heme to acytochromes c and c1 (Cyc1p and Cyt1p)
PGI1	1.76	Glycolytic enzyme phosphoglucose isomerase; catalyzes the interconversion of glucose-6-phosphate and fructose-6-phosphate; required for cell cycle progression and completion of the gluconeogenic events of sporulation
GVP36	1.76	BAR domain protein that localizes to early and late Golgi vesicles; required for adaptation to varying nutrient concentrations, fluid-phase endocytosis, polarization of the actin cytoskeleton, and vacuole biogenesis
PRP42	1.75	U1 snRNP protein involved in splicing; required for U1 snRNP biogenesis; contains multiple tetriatricopeptide repeats
ESL2	1.75	hEST1A/B (SMG5/6)-like protein; contributes to environment-sensing adaptive gene expression responses; Esl2p and Esl1p contain a 14-3-3-like domain and a putative PilT N-terminus ribonuclease domain; interacts with Pex14p; may interact with ribosomes, based on co-purification experiments; green fluorescent protein (GFP)-fusion protein localizes to the nucleus and cytoplasm; ESL2 has a paralog, ESL1, that arose from the whole genome duplication
MTC7	1.74	Protein of unknown function; predicted metabolic role based on network analysis derived from ChIP experiments, a large-scale deletion study and localization of transcription factor binding sites; null mutant is sensitive to temperature oscillation in a cdc13-1 mutant
YIR020W-A	1.74	Dubious open reading frame; unlikely to encode a functional protein, based on available experimental and comparative sequence data
HEK2	1.74	RNA binding protein involved in asymmetric localization of ASH1 mRNA; represses translation of ASH1 mRNA, an effect reversed by Yck1p-dependent phosphoryation; regulates telomere position effect and length; similarity to hnRNP-K; increases mRNA stability by binding (CNN)n repeat elements
SPT5	1.74	Spt4p/5p (DSIF) transcription elongation factor complex subunit; the Spt4/5 complex binds to ssRNA in a sequence-specific manner, and in concert with RNAP I and II has multiple roles regulating transcriptional elongation, RNA processing, quality control, and transcription-coupled repair; interacts with DNA upstream of RNAPII and the non-template strand of the transcription bubble; Spt5p is the only transcription elongation factor conserved in all domains of life
SND2	1.74	Protein involved in SRP-independent targeting of substrates to the ER; component of an alternative ER targeting pathway that has partial functional redundancy with the GET pathway; preference for substrates with downstream transmembrane domains; interacts with Snd1p, Pho88p/Snd3p and Sec61p-translocon subunits; can compensate for loss of SRP; role in the late endosome-vacuole interface; putative role in secretory protein quality control
PEX6	1.73	AAA-peroxin; heterodimerizes with AAA-peroxin Pex1p and participates in the recycling of peroxisomal signal receptor Pex5p from the peroxisomal membrane to the cystosol; mutations in human PEX6 can lead to severe peroxisomal disorders and early death
GAA1	1.73	Subunit of the GPI:protein transamidase complex; removes the GPI-anchoring signal and attaches GPI (glycosylphosphatidylinositol) to proteins in the ER; human homolog GPAA1 can complement growth defects of yeast thermosensitive mutant at restrictive temperature
USB1	1.73	Putative poly(U)-specific 3'-to-5' RNA exonuclease; involved in 3'-end processing of U6 snRNA removing uridines and generating a terminal 2?,3? cyclic phosphate; essential protein that localizes to the nucleus and mitochondria; overexpression suppresses the respiratory defects of oxa1 and mtf2 mutants; homolog of S.pombe gene, mpn1 and human gene, hUSB1; mutations in hUSB1 are associated with a rare genodermatosis, poikiloderma with neutropenia (OMIM 604173)
SLM1	1.73	Phosphoinositide PI4,5P(2) binding protein, forms a complex with Slm2p; acts downstream of Mss4p in a pathway regulating actin cytoskeleton organization in response to stress; TORC2 complex substrate and effector; protein abundance increases in response to DNA replication stress; SLM1 has a paralog, SLM2, that arose from the whole genome duplication
GAT1	1.73	Transcriptional activator of nitrogen catabolite repression genes; contains a GATA-1-type zinc finger DNA-binding motif; activity and localization regulated by nitrogen limitation and Ure2p; different translational starts produce two major and two minor isoforms that are differentially regulated and localized
YMR209C	1.72	Putative S-adenosylmethionine-dependent methyltransferase; SWAT-GFP and mCherry fusion proteins localize to the endoplasmic reticulum and vacuole respectively; YMR209C is not an essential gene
RFC4	1.72	Subunit of heteropentameric Replication factor C (RF-C); which is a DNA binding protein and ATPase that acts as a clamp loader of the proliferating cell nuclear antigen (PCNA) processivity factor for DNA polymerases delta and epsilon; relocalizes to the cytosol in response to hypoxia
IDH2	1.72	Subunit of mitochondrial NAD(+)-dependent isocitrate dehydrogenase; complex catalyzes the oxidation of isocitrate to alpha-ketoglutarate in the TCA cycle; phosphorylated
SLT2	1.71	Serine/threonine MAP kinase; coordinates expression of all 19S regulatory particle assembly-chaperones (RACs) to control proteasome abundance; involved in regulating maintenance of cell wall integrity, cell cycle progression, nuclear mRNA retention in heat shock, septum assembly; required for mitophagy, pexophagy; affects recruitment of mitochondria to phagophore assembly site; plays role in adaptive response of cells to cold; regulated by the PKC1-mediated signaling pathway
SWH1	1.71	Protein similar to mammalian oxysterol-binding protein; contains ankyrin repeats and FFAT motif; interacts with ER anchor Scs2p at the nucleus-vacuole junction; regulated by sterol binding; SWH1 has a paralog, OSH2, that arose from the whole genome duplication
NTA1	1.71	Amidase; removes the amide group from N-terminal asparagine and glutamine residues to generate proteins with N-terminal aspartate and glutamate residues that are targets of ubiquitin-mediated degradation
YLR177W	1.70	Putative protein of unknown function; phosphorylated by Dbf2p-Mob1p in vitro; some strains contain microsatellite polymophisms at this locus; not an essential gene; YLR177W has a paralog, PSP1, that arose from the whole genome duplication
ATP19	1.70	Subunit k of the mitochondrial F1F0 ATP synthase; F1F0 ATP synthase is a large, evolutionarily conserved enzyme complex required for ATP synthesis; associated only with the dimeric form of ATP synthase
ARA2	1.70	NAD-dependent arabinose dehydrogenase; involved in biosynthesis of dehydro-D-arabinono-1,4-lactone; similar to plant L-galactose dehydrogenase
SDH3	1.70	Subunit of succinate dehydrogenase and of TIM22 translocase; functions as cytochrome b subunit of succinate dehydrogenase, which couples oxidation of succinate to transfer of electrons to ubiquinone as part of the TCA cycle and the mitochondrial respiratory chain; also required for mitochondrial inner membrane protein import as part of the TIM22 complex; SDH3 has a paralog, SHH3, that arose from the whole genome duplication
YLR076C	1.70	Dubious open reading frame; unlikely to encode a functional protein, based on available experimental and comparative sequence data; partially overlaps the essential gene RPL10 which encodes the ribosomal protein
YDR157W	1.70	Putative protein of unknown function; conserved across S. cerevisiae strains
VRG4	1.69	Golgi GDP-mannose transporter; regulates Golgi function and glycosylation in Golgi; VRG4 has a paralog, HVG1, that arose from the whole genome duplication
MID1	1.69	Stretch-activated Ca2+-permeable cation channel; required for Ca2+ influx stimulated by mating pheromones and some abiotic stresses; sensor of compressive mechanical stress; N-glycosylated membrane protein of the ER and plasma membrane; interacts with Cch1p; oligomerizes; is an alpha2/delta-like protein and participates in the generation of stretch-activated Ca2+-permeable cation channel activity when expressed in mammalian cells
COX20	1.69	Mitochondrial inner membrane protein; required for proteolytic processing of Cox2p and its assembly into cytochrome c oxidase
FCY2	1.68	Purine-cytosine permease; mediates purine (adenine, guanine, and hypoxanthine) and cytosine accumulation; relative distribution to the vacuole increases upon DNA replication stress
BSP1	1.68	Adapter that links synaptojanins to the cortical actin cytoskeleton; the synaptojanins are Inp52p and Inp53p
MRPL11	1.67	Mitochondrial ribosomal protein of the large subunit; localizes to vacuole in response to H2O2
YJR098C	1.67	Putative protein of unknown function; the authentic, non-tagged protein is detected in highly purified mitochondria in high-throughput studies
TIR4	1.67	Cell wall mannoprotein; expressed under anaerobic conditions and required for anaerobic growth; transcription is also induced by cold shock; member of the Srp1p/Tip1p family of serine-alanine-rich protein

FSF1	1.67	Putative protein; predicted to be an alpha-isopropylmalate carrier; belongs to the sideroblastic-associated protein family; non-tagged protein is detected in purified mitochondria; likely to play a role in iron homeostasis
NOP2	1.67	rRNA m5C methyltransferase; methylates cytosine at position 2870 of 25S rRNA; has an essential function independent of rRNA methylation; contains seven beta-strand methyltransferase motif; essential for processing and maturation of 27S pre-rRNA and large ribosomal subunit biogenesis; localized to the nucleolus; constituent of 66S pre-ribosomal particles; rRNA methylation defect and lethality are functionally complemented by human NOP2, a gene upregulated in cancer
HOT1	1.67	Transcription factor for glycerol biosynthetic genes; required for the transient induction of glycerol biosynthetic genes GPD1 and GPP2 in response to high osmolarity; targets Hog1p to osmostress responsive promoters; has similarity to Msn1p and Gcr1p
RTS1	1.66	B-type regulatory subunit of protein phosphatase 2A (PP2A); Rts1p and Cdc55p are alternative regulatory subunits for PP2A catalytic subunits, Pph21p and Pph22p; PP2A-Rts1p protects cohesin when recruited by Sgo1p to the pericentromere; highly enriched at centromeres in the absence of Cdc55p; required for maintenance of septin ring organization during cytokinesis, for ring disassembly in G1 and for dephosphorylation of septin, Shs1p; homolog of the mammalian B' subunit of PP2A
YMR173W-A	1.66	Dubious open reading frame; unlikely to encode a functional protein, based on available experimental and comparative sequence data; overlaps the verified gene DDR48/YML173W
YEN1	1.65	Holliday junction resolvase; promotes template switching during break-induced replication (BIR), causing non-reciprocal translocations (NRTs); localization is cell-cycle dependent and regulated by Cdc28p phosphorylation; homolog of human GEN1; similar to S. cerevisiae endonuclease Rth1
SAM1	1.65	S-adenosylmethionine synthetase; catalyzes transfer of the adenosyl group of ATP to the sulfur atom of methionine; SAM1 has a paralog, SAM2, that arose from the whole genome duplication
MVP1	1.65	Protein required for sorting proteins to the vacuole; Mvp1p and Vps1p act in concert to promote membrane traffic to the vacuole; participates in transcription initiation and/or early elongation of specific genes; interacts with 'foot domain' of RNA polymerase II; deletion results in abnormal CTD-Ser5 phosphorylation of RNA polymerase II at specific promoter regions; protein abundance increases in response to DNA replication stress
YPL077C	1.65	Putative protein of unknown function; regulates PIS1 expression; mutant displays spore wall assembly defect in ether sensitivity screen; YPL077C is not an essential gene; YPL077C has a paralog, YBR197C, that arose from the whole genome duplication
YOR390W	1.65	Protein involved in fluoride export; nearly identical to FEX2, and deletion of both proteins results in a large increase in fluoride sensitivity compared with the single mutant; contains two FEX domains connected by a linker; part of a widespread family of conserved fluoride export proteins
SIN4	1.64	Subunit of the RNA polymerase II mediator complex; associates with core polymerase subunits to form the RNA polymerase II holoenzyme; contributes to both positive and negative transcriptional regulation; dispensible for basal transcription
IRC16	1.64	Putative protein of unknown function; partially overlaps verified gene ERV2/YPR037C but doesn't share phenotypes; also overlaps dubious ORF YPR039W; null mutant displays increased levels of spontaneous Rad52p foci, increased biofilm information, different altered sensitivities to various chemcicals
SCW10	1.64	Cell wall protein with similarity to glucanases; may play a role in conjugation during mating based on mutant phenotype and its regulation by Ste12p; SWC10 has a paralog, SCW4, that arose from the whole genome duplication
ERB1	1.64	Constituent of 66S pre-ribosomal particles; forms a complex with Nop7p and Ytm1p that is required for maturation of the large ribosomal subunit; required for maturation of the 25S and 5.8S ribosomal RNAs; binds RNA via its C-terminal domain; homologous to mammalian Bop1
CWC25	1.64	Splicing factor required for the first step of pre-mRNA splicing; binding to the spliceosome requires Prp2p and Yju2p; heat-stable protein; has similarity to S. pombe Cwf25p
YEH2	1.64	Steryl ester hydrolase; catalyzes steryl ester hydrolysis at the plasma membrane; involved in sterol metabolism; YEH2 has a paralog, YEH1, that arose from the whole genome duplication
HUT1	1.64	Protein with a role in UDP-galactose transport to the Golgi lumen; has similarity to human UDP-galactose transporter UGTrel1, exhibits a genetic interaction with S. cerevisiae ERO1
YGL057C	1.64	Protein of unknown function; null mutant exhibits a respiratory growth defect and synthetic interactions with prohibitin (phb1) and gem1; authentic, non-tagged protein is detected in highly purified mitochondria in high-throughput studies
TFB4	1.64	Subunit of TFIIH complex; involved in transcription initiation, similar to 34 kDa subunit of human TFIIH; interacts with Ssl1p
NAN1	1.63	U3 snoRNP protein; component of the small (ribosomal) subunit (SSU) processosome containing U3 snoRNA; required for the biogenesis of18S rRNA
YIL152W	1.63	Putative protein of unknown function
COG2	1.63	Essential component of the conserved oligomeric Golgi complex; a cytosolic tethering complex that functions in protein trafficking to mediate fusion of transport vesicles to Golgi compartments; the components of the Golgi complex are Gog1p through Cog8p
MSN2	1.63	Stress-responsive transcriptional activator; activated in stochastic pulses of nuclear localization in response to various stress conditions; binds DNA at stress response elements of responsive genes; light sensing pathway component that accumulates in the nucleus in response to blue light; relative distribution to nucleus increases upon DNA replication stress
ROQ1	1.63	Ub-ligase substrate-specificity factor; proteolytically-cleaved form acts as a pseudosubstrate, binding and altering the substrate specificity of Ubr1p towards misfolded and native ER membrane and cytosolic proteins, as part of the stress-induced homeostatically regulated protein degradation (SHRED) pathway; hydrophilin essential during desiccation-rehydration; induced by osmotic stress, starvation and during stationary phase; protein abundance increases in response to DNA replication stress
RAD6	1.63	Ubiquitin-conjugating enzyme (E2); involved in postreplication repair as a heterodimer with Rad18p, regulation of K63 polyubiquitination in response to oxidative stress, DSBR and checkpoint control as a heterodimer with Bre1p, ubiquitin-mediated N-end rule protein degradation as a heterodimer with Ubr1p, ERAD with Ubr1p in the absence of canonical ER membrane ligases, and Rpn4p turnover as part of proteasome homeostasis, in complex with Ubr2p and Mub1p
YCH1	1.62	Phosphatase with sequence similarity to Mih1p; member of the Cdc25p subfamily of tyrosine phosphatases and Arr2p, an arsenate reductase, based on the presence of a rhodanese-homology domain; green fluorescent
CYB2	1.62	Cytochrome b2 (L-lactate cytochrome-c oxidoreductase); component of the mitochondrial intermembrane space, required for lactate utilization; expression is repressed by glucose and anaerobic conditions
YDL162C	1.62	Dubious open reading frame; unlikely to encode a functional protein, based on available experimental and comparative sequence data; partially overlaps ENT1/YDL161W, a verified gene involved in endocytosis and actin cortical patch assembly
YIL171W-A	1.61	Dubious open reading frame; unlikely to encode a functional protein, based on available experimental and comparative sequence data; overlaps ORF IMA3/YIL172C
MNN11	1.61	Subunit of a Golgi mannosyltransferase complex; this complex also contains Anp1p, Mnn9p, Mnn10p, and Hoc1p, and mediates elongation of the polysaccharide mannan backbone; has homology to Mnn10p
POT1	1.61	3-ketoacyl-CoA thiolase with broad chain length specificity; cleaves 3-ketoacyl-CoA into acyl-CoA and acetyl-CoA during beta-oxidation of fatty acids
WTM1	1.61	Transcriptional modulator; involved in regulation of meiosis, silencing, and expression of RNR genes; acts as a nuclear anchor to retain the ribonucleotide reductase small subunit heterodimer, Rnr2p-Rnr4p, in the nucleus when cells are not in S phase; contains WD repeats
ENT1	1.60	Epsin-like protein involved in endocytosis and actin patch assembly; K63-specific Ub chain binding protein that functions as an endocytic adaptor; binds clathrin via a clathrin-binding domain motif at C-terminus; contains two ubiquitin-interacting motifs (UIMs); functionally redundant with Ent2p; relocalizes from bud neck to cytoplasm upon DNA replication stress; ENT1 has a paralog, ENT2, that arose from the whole genome duplication
YPR127W	1.60	Putative pyridoxine 4-dehydrogenase; differentially expressed during alcoholic fermentation; expression activated by transcription factor YRM1/YOR172W; green fluorescent protein (GFP)-fusion protein localizes to both the cytoplasm and the nucleus
SSP120	1.60	Protein packaged into COPII vesicles for ER to Golgi trafficking; forms a complex with Emp47p that may function in trafficking plasma membrane glycoproteins through early secretory pathway; green fluorescent protein (GFP)-fusion protein localizes to the cytoplasm in a punctate pattern
MGT1	1.60	DNA repair methyltransferase (6-O-methylguanine-DNA methylase); involved in protection against DNA alkylation damage; localizes to the peroxisome in a Pex5p-dependent manner
ALD4	1.60	Mitochondrial aldehyde dehydrogenase; required for growth on ethanol and conversion of acetaldehyde to acetate; phosphorylated; activity is K+ dependent; utilizes NADP+ or NAD+ equally as coenzymes; expression is glucose repressed; can substitute for cytosolic NADP-dependent aldehyde dehydrogenase when directed to the cytosol; human homolog ALDH2 can complement yeast ald4 mutant
NOG1	1.60	Putative GTPase; associates with free 60S ribosomal subunits in the nucleolus and is required for 60S ribosomal subunit biogenesis; constituent of 66S pre-ribosomal particles; member of the ODN family of nucleolar G-proteins
IOC4	1.59	Member of a complex (Isw1b) with Isw1p and Ioc2p; interacts directly with H3K36me3 nucleosomes through its PWWP domain to recruit the Isw1b complex to open reading frames in a Set2p-dependent manner; Isw1b exhibits nucleosome-stimulated ATPase activity and acts within coding regions to coordinate transcription elongation with termination and processing
ISU1	1.59	Conserved protein of the mitochondrial matrix; performs a scaffolding function during assembly of iron-sulfur clusters, interacts physically and functionally with yeast frataxin (Yfh1p); ISU1 has a paralog, ISU2, that arose from the whole genome duplication; isu1 isu2 double mutant is inviable; human homolog ISCU implicated in mitochondrial myopathy, can complement isu1 isu2 double mutant
DIB1	1.58	17-kDa component of the U4/U6aU5 tri-snRNP; plays an essential role in pre-mRNA splicing; human ortholog TXNL4A (the human U5-specific 15-kDa protein) complements yeast dib1 null mutant
PCI8	1.58	Possible shared subunit of Cop9 signalosome (CSN) and eIF3; binds eIF3b subunit Prt1p, has possible dual functions in transcriptional and translational control, contains a PCI (Proteasome-COP9 signalosome (CSN)-eIF3) domain
ELG1	1.58	Subunit of an alternative replication factor C complex; important for DNA replication and genome integrity; suppresses spontaneous DNA damage; involved in homologous recombination-mediated repair and telomere homeostasis; required for PCNA (Pol30p) unloading during DNA replication
SNU56	1.58	Component of U1 snRNP required for mRNA splicing via spliceosome; yeast specific, no metazoan counterpart; interacts with mRNA in commitment complex
PBN1	1.58	Component of glycosylphosphatidylinositol-mannosyltransferase I; essential component; required for the autocatalytic post-translational processing of the protease B precursor Prb1p; localizes to ER in lumenal orientation; homolog of mammalian PIG-X
ARP1	1.57	Actin-related protein of the dynactin complex; required for spindle orientation and nuclear migration; forms actin-like short filament composed of 9 or 10 Arp1p monomers; putative ortholog of mammalian centractin
APJ1	1.57	Hsp40 chaperone with a role in SUMO-mediated protein degradation; works in concert with Hsp70 and Hsp110 (Sse1p) to promote disaggregation of intranuclear protein inclusions; competes with Hsp104 in disaggregation, supporting turnover instead of refolding; member of DnaJ-like family, conserved across eukaryotes; overexpression interferes with propagation of the [Psi+] prion; forms nuclear foci upon DNA replication stres
MMM1	1.57	ER integral membrane protein, ERMES complex subunit; ERMES links the ER to mitochondria and may promote inter-organellar calcium and phospholipid exchange as well as coordinating mitochondrial DNA replication and growth; required for mitophagy; ERMES complex is often co-localized with peroxisomes and with concentrated areas of pyruvate dehydrogenase; localizes to the peroxisome in glucose
NAM8	1.57	RNA binding protein, component of the U1 snRNP protein; mutants are defective in meiotic recombination and in formation of viable spores, involved in the formation of DSBs through meiosis-specific splicing of REC107 pre-mRNA; Nam8p regulon embraces the meiotic pre-mRNAs of REC107, HFM1, AMA1 SPO22 and PCH2; the putative RNA binding domains RRM2 and RRM3 are required for Nam8p meiotic function
SDD3	1.57	Putative metalloprotease; overproduction suppresses lethality due to expression of the dominant PET9 allele AAC2-A128P
MIF2	1.57	Centromeric CDEIII region binding protein; nucleates kinetochore assembly; required for the structural integrity of elongating spindles; copurifies with subunits of the MIND complex and centromeric nucleosome components (Cse4p and histones H2A, H2B, and H4); phosphorylated by Ipl1p; orthologous to human centromere constitutive-associated network (CCAN) subunit CENP-C and fission yeast cnp3; localizes to the kinetochore
TOS6	1.57	Glycosylphosphatidylinositol-dependent cell wall protein; expression is periodic and decreases in respone to ergosterol perturbation or upon entry into stationary phase; depletion increases resistance to lactic acid
SIN3	1.57	Component of both the Rpd3S and Rpd3L histone deacetylase complexes; involved in transcriptional repression and activation of diverse processes, including mating-type switching and meiosis; involved in the maintenance of chromosomal integrity
INA1	1.56	Protein of unknown function; not an essential gene; YLR413W has a paralog, FAT3, that arose from the whole genome duplicatio
YHR112C	1.56	Protein of unknown function; localizes to the cytoplasm and nucleus; overexpression affects protein trafficking through the endocytic pathway
OGG1	1.55	Nuclear and mitochondrial glycosylase/lyase; specifically excises 7,8-dihydro-8-oxoguanine residues located opposite cytosine or thymine residues in DNA, repairs oxidative damage to mitochondrial DNA, contributes to UVA resistance
GPI12	1.55	ER membrane protein involved in the second step of GPI anchor assembly; the second step is the de-N-acetylation of the N-acetylglucosaminylphosphatidylinositol intermediate; functional homolog of human PIG-Lp; GPI stands for glycosylphosphatidylinositol
YLR262C-A	1.55	Protein of unknown function that associates with ribosomes; null mutant exhibits translation defects, altered polyribosome profiles, and resistance to the translation inhibitor anisomcyin; protein abundance increases in response to DNA replication stress
SYH1	1.54	Protein of unknown function that influences nuclear pore distribution; co-purifies with ribosomes; contains a GYF domain, which bind proline-rich sequences; deletion extends chronological lifespan; SYH1 has a paralog, SMY2, that arose from the whole genome duplication
MPD1	1.54	Member of the protein disulfide isomerase (PDI) family; interacts with and inhibits the chaperone activity of Cne1p; MPD1 overexpression in a pdi1 null mutant suppresses defects in Pdi1p functions such as carboxypeptidase Y maturation
ZIM17	1.54	Protein co-chaperone with a zinc finger motif; essential for protein import into mitochondria; may act with Pam18p to facilitate recognition and folding of imported proteins by Ssc1p (mtHSP70) in the mitochondrial matrix; required for the maintenance of Ssc1p solubility and assists in the functional interaction of Ssc1p with substrate proteins
PSA1	1.54	GDP-mannose pyrophosphorylase (mannose-1-phosphate guanyltransferase); synthesizes GDP-mannose from GTP and mannose-1-phosphate in cell wall biosynthesis; required for normal cell wall structure
SCW4	1.54	ell wall protein with similarity to glucanases; scw4 scw10 double mutants exhibit defects in mating; SCW4 has a paralog, SCW10, that arose from the whole genome duplication
SDC25	1.54	Non-essential Ras guanine nucleotide exchange factor (GEF); localized to the membrane; expressed in poor nutrient conditions and on nonfermentable carbon sources; contains a stop codon in S288C, full-length gene includes YLL017W; SDC25 has a paralog, CDC25, that arose from the whole genome duplication
YJR124C	1.54	Putative protein of unknown function; expression induced under calcium shortage
AIM32	1.53	2Fe-2S mitochondrial protein involved in redox quality control; may maintain protein redox status by targeting oxidation sensitive cysteine residues; bishistidinyl coordinated, non-Rieske [2Fe-2S] cluster containing thiredoxin-like ferredoxin; forms a functional complex with Osm1p and Erv1p in the IMS; required for assembly of numerous mitochondrial import complexes; essential for anaerobiosis; localizes to the mitochondrial matrix and intermembrane space (IMS); homologous to APD1
ARF3	1.53	Glucose-repressible ADP-ribosylation factor; GTPase of Ras superfamily involved in regulating cell polarity and invasive growth; localizes to dynamic spots at plasma membrane and modulates PtdIns(4,5)P2 levels to facilitate endocytosis; required for localization of endocytic protein Lsb5p to correct cortical site in cells; also has mRNA binding activity; homolog of mammalian Arf6
SIW14	1.53	Inositol phosphatase involved in inositol pyrophosphate metabolism; hydrolyzes the beta-phosphate from the 5-diphosphate group of PP-InsPs including: 5PP-IP5 (IP7), 1,5PP-IP4 (IP8) and 5-PP-InsP4; functions as an anti-prion (anti-[PSI]) component; member of the atypical dual-specificity subgroup of the protein tyrosine-phosphatase (PTP) superfamily; localizes to the cytoplasm
TPM2	1.53	Minor isoform of tropomyosin; binds to and stabilizes actin cables and filaments, which direct polarized cell growth and the distribution of several organelles; appears to have distinct and also overlapping functions with Tpm1p; TPM2 has a paralog, TPM1, that arose from the whole genome duplication
PRM10	1.52	Pheromone-regulated protein; proposed to be involved in mating; predicted to have 5 transmembrane segments; induced by treatment with 8-methoxypsoralen and UVA irradiation
HOS3	1.52	Trichostatin A-insensitive homodimeric histone deacetylase (HDAC); specificity in vitro for histones H3, H4, H2A, and H2B; similar to Hda1p, Rpd3p, Hos1p, and Hos2p; deletion results in increased histone acetylation at rDNA repeats
YKR015C	1.52	Putative protein of unknown function
ATP7	1.52	Subunit d of the stator stalk of mitochondrial F1F0 ATP synthase; F1F0 ATP synthase is a large, evolutionarily conserved enzyme complex required for ATP synthesis
YDR269C	1.52	Dubious open reading frame; unlikely to encode a functional protein, based on available experimental and comparative sequence data
YEL073C	1.52	Putative protein of unknown function; located adjacent to ARS503 and the telomere on the left arm of chromosome V; regulated by inositol/choline
YAP6	1.52	Basic leucine zipper (bZIP) transcription factor; physically interacts with the Tup1-Cyc8 complex and recruits Tup1p to its targets; overexpression increases sodium and lithium tolerance; computational analysis suggests a role in regulation of expression of genes involved in carbohydrate metabolism; YAP6 has a paralog, CIN5, that arose from the whole genome duplication
YLR407W	1.51	Putative protein of unknown function; null mutant displays elongated buds and a large fraction of budded cells have only one nucleus
RAD51	1.51	Strand exchange protein; forms helical filament with DNA that searches for homology; involved in recombinational repair of DNA DSBs during vegetative growth and meiosis; phosphorylation by Cdc28p in G2/M phase promotes DNA binding, strand invasion, and primer extension; L1 and L2 motifs of DNA binding site I have critical roles in ensuring DNA fidelity during meiotic recombination; RAD51 and RAD4 pathways confer resistance to benzo[a]pyrene dihydrodiol; homolog of Dmc1p and bacterial RecA
MPS2	1.51	Essential membrane protein localized at nuclear envelope and SPBs; required for insertion of the newly duplicated spindle pole body into the nuclear envelope; potentially phosphorylated by Cdc28p; MPS2 has a paralog, CSM4, that arose from the whole genome duplication
CPR8	1.51	Peptidyl-prolyl cis-trans isomerase (cyclophilin); catalyzes the cis-trans isomerization of peptide bonds N-terminal to proline residues; potential role in the secretory pathway; CPR8 has a paralog, CPR4, that arose from the whole genome duplication
GDH1	1.51	NADP(+)-dependent glutamate dehydrogenase; synthesizes glutamate from ammonia and alpha-ketoglutarate; rate of alpha-ketoglutarate utilization differs from Gdh3p; expression regulated by nitrogen and carbon sources; GDH1 has a paralog, GDH3, that arose from the whole genome duplication
MRPL24	1.50	Mitochondrial ribosomal protein of the large subunit; two mitochondrial ribosomal proteins, YmL14 and YmL24, have been assigned to the same gene
YPR053C	1.50	Putative protein of unknown function; conserved among S. cerevisiae strains; YPR053C is not an essential gene; partially overlaps verified ORF NHP6A/YPR052C
PAU7	-1.50	Member of the seripauperin multigene family; active during alcoholic fermentation, regulated by anaerobiosis, inhibited by oxygen, repressed by heme
SAP185	-1.50	Protein that forms a complex with the Sit4p protein phosphatase; required for Sit4p function; member of a family of similar proteins including Sap4p, Sap155p, and Sap190p; SAP185 has a paralog, SAP190, that arose from the whole genome duplication
YGR277C	-1.50	Subunit of the CoA-Synthesizing Protein Complex (CoA-SPC); subunits of this complex are: Cab2p, Cab3p, Cab4p, Cab5p, Sis2p and Vhs3p; involved in histone acylation; probable pantetheine-phosphate adenylyltransferase (PPAT); PPAT catalyzes the fourth step in the biosynthesis of coenzyme A from pantothenate; null mutant lethality is complemented by E. coli coaD (encoding PPAT) and by human COASY
YDL156W	-1.51	Nuclear protein with role in transcription, protein quality control; localizes to the intranuclear quality control compartment (INQ) in response to proteasome inhibition or DNA replication stress; INQ likely sequesters proteins involved in DNA metabolism for degradation or re-folding; also localizes to coding regions of transcribed genes; contains three WD domains (WD-40 repeat); human ortholog WDR76 also exhibits perinuclear localization under similar stress conditions
SYO1	-1.51	Transport adaptor or symportin; assembly chaperone that co-translationally associates with nascent Rpl5p, preventing aggregation; facilitates synchronized nuclear coimport of two 5S-rRNA binding proteins, Rpl5p and Rpl11p, mediated by import receptor Kap104p; required for biogenesis of the large ribosomal subunit; green fluorescent protein (GFP)-fusion protein localizes to the cytoplasm and nucleus
SRD1	-1.51	Protein involved in the processing of pre-rRNA to mature rRNA; contains a C2/C2 zinc finger motif; srd1 mutation suppresses defects caused by the rrp1-1 mutatio
KRE29	-1.52	Subunit of the SMC5-SMC6 complex; this complex is involved in removal of X-shaped DNA structures that arise between sister chromatids during DNA replication and repair; heterozygous mutant shows haploinsufficiency in K1 killer toxin resistance
YPL191C	-1.52	K48-specific deubiquitinating (DUB) enzyme; MINDY family endo-type deubiquitinase that preferentially cleaves long K48-linked polyubiquitin chains between moieties; diploid deletion strain exhibits high budding index; GFP-fusion protein localizes to the cytoplasm endoplasmic reticulum and cell periphery in high-throughput studies; YPL191C has a paralog, YGL082W, that arose from the whole genome duplication; ortholog of human MINDY2/FAM63B
FAU1	-1.52	5,10-methenyltetrahydrofolate synthetase; involved in folic acid biosynthesis
PEX27	-1.52	Peripheral peroxisomal membrane protein; involved in controlling peroxisome size and number, interacts with Pex25p; PEX27 has a paralog, PEX25, that arose from the whole genome duplication
SFG1	-1.52	Putative transcription factor; induces superficial pseudohyphal growth, positively regulates invasive growth, but is not required for invasive pseudohyphal growth; may act together with Phd1p; promotes cell adhesion independent of Flo11p by repressing genes that encode cell wall degrading enzymes; localizes to the nucleus; potential Cdc28p substrate
YBL111C	-1.53	Helicase-like protein encoded within the telomeric Y' element; relocalizes from mitochondrion to cytoplasm upon DNA replication stress
MRPS8	-1.53	Mitochondrial ribosomal protein of the small subunit
MKC7	-1.53	GPI-anchored aspartyl protease; member of the yapsin family of proteases involved in cell wall growth and maintenance; shares functions with Yap3p and Kex2p; MKC7 has a paralog, YPS1, that arose from the whole genome duplication
SSQ1	-1.53	Mitochondrial hsp70-type molecular chaperone; required for assembly of iron/sulfur clusters into proteins at a step after cluster synthesis; involved in maturation of Grx5p and Yfh1p, which is a homolog of human frataxin implicated in Friedreich's ataxia
FPR2	-1.54	Membrane-bound peptidyl-prolyl cis-trans isomerase (PPIase); binds to the drugs FK506 and rapamycin; expression pattern suggests possible involvement in ER protein trafficking; relocalizes from nucleus to vacuole upon DNA replication stress; mutation is functionally complemented by human FKBP2
TEF1	-1.54	Translational elongation factor EF-1 alpha; GTP-bound active form, binds to and delivers aminoacylated tRNA to the A-site of ribosomes for elongation of nascent polypeptides; moonlighting function as an actin binding and bundling protein; association with GTPase Rho1p on the vacuolar membrane may facilitate F-actin remodeling; involved in tRNA re-export from the nucleus
YSW1	-1.54	rotein required for normal prospore membrane formation; interacts with Gip1p, which is the meiosis-specific regulatory subunit of the Glc7p protein phosphatase; expressed specifically in spores and localizes to the prospore membrane; YSW1 has a paralog, SPO21, that arose from the whole genome duplication
RPL19B	-1.54	Ribosomal 60S subunit protein L19B; rpl19a and rpl19b single null mutations result in slow growth, while the double null mutation is lethal; homologous to mammalian ribosomal protein L19, no bacterial homolog; RPL19B has a paralog, RPL19A, that arose from the whole genome duplication
ULI1	-1.55	Protein of unknown function; involved in and induced by the endoplasmic reticulum unfolded protein response (UPR); SWAT-GFP and mCherry fusion proteins localize to the endoplasmic reticulum
PIM1	-1.55	ATP-dependent Lon protease; involved in degradation of misfolded mitochondrial proteins; required for mitochondrial maintenance and biogenesis; regulates mitochondrial DNA copy number with Mrx6p; subunit of a complex containing Mrx6p, Pet20p, and Mam33p that may regulate mtDNA replication; protease-independent, chaperone-like function in mitochondrial membrane complex assembly; localizes to the mitochondrial matrix
MTW1	-1.55	Essential component of the MIND kinetochore complex; joins kinetochore subunits contacting DNA to those contacting microtubules; critical to kinetochore assembly; complex consists of Mtw1p Including Nnf1p-Nsl1p-Dsn1p (MIND)
HPR5	-1.55	DNA helicase and DNA-dependent ATPase; role in DNA repair and checkpoint recovery, in the proper timing of commitment to meiotic recombination and the Meiosis I to II transition; blocks trinucleotide repeat expansion; affects genome stability; disassembles Rad51p nucleoprotein filaments during meiotic recombination; stimulates Mus81p-Mms4p endonuclease activity independent of catalytic activity; ATPase and ssDNA translocating motor activities inhibited by Dmc1p; functional homolog of human RTEL1
YHR125W	-1.56	Putative protein of unknown function; conserved across S. cerevisiae strains
PGI1	-1.56	Glycolytic enzyme phosphoglucose isomerase; catalyzes the interconversion of glucose-6-phosphate and fructose-6-phosphate; required for cell cycle progression and completion of the gluconeogenic events of sporulation

COR1	-1.56	Core subunit of the ubiquinol-cytochrome c reductase complex; the ubiquinol-cytochrome c reductase complex (bc1 complex) is a component of the mitochondrial inner membrane electron transport chain
PPM2	-1.56	AdoMet-dependent tRNA methyltransferase; also involved in methoxycarbonylation; required for the synthesis of wybutosine (yW), a modified guanosine found at the 3'-position adjacent to the anticodon of phe-tRNA; similarity to Ppm1p
PMT4	-1.57	Protein O-mannosyltransferase; transfers mannose residues from dolichyl phosphate-D-mannose to protein serine/threonine residues; appears to form homodimers in vivo and does not complex with other Pmt proteins; target for new antifungals
PAN5	-1.57	2-dehydropantoate 2-reductase; part of the pantothenic acid pathway, structurally homologous to E. coli panE
PWP2	-1.58	Conserved 90S pre-ribosomal component; essential for proper endonucleolytic cleavage of the 35 S rRNA precursor at A0, A1, and A2 sites; contains eight WD-repeats; PWP2 deletion leads to defects in cell cycle and bud morphogenesis
ACH1	-1.58	Protein with CoA transferase activity; particularly for CoASH transfer from succinyl-CoA to acetate; has minor acetyl-CoA-hydrolase activity; phosphorylated; required for acetate utilization and for diploid pseudohyphal growth
HMS1	-1.58	bHLH protein with similarity to myc-family transcription factors; overexpression confers hyperfilamentous growth and suppresses the pseudohyphal filamentation defect of a diploid mep1 mep2 homozygous null mutant
ASN1	-1.58	Asparagine synthetase; catalyzes the synthesis of L-asparagine from L-aspartate in the asparagine biosynthetic pathway; ASN1 has a paralog, ASN2, that arose from the whole genome duplication
SEC13	-1.58	Structural component of 3 complexes; subunit of the Nup84p nuclear pore subcomplex that contributes to nucleocytoplasmic transport and NPC biogenesis; subunit of the COPII vesicle coat required for ER-to-Golgi transport; subunit of SEACAT, a subcomplex of the coatomer-related, vacuolar-associated SEA complex, that inhibits the TORC1 inhibitory role of SEACIT (Iml1p-Npr2p-Npr3p), a GAP for Gtr1p, thereby resulting in activation of TORC1 signaling; human SEC13 homolog
RKI1	-1.58	Ribose-5-phosphate ketol-isomerase; catalyzes the interconversion of ribose 5-phosphate and ribulose 5-phosphate in the pentose phosphate pathway; participates in pyridoxine biosynthesis
MET3	-1.58	ATP sulfurylase; catalyzes the primary step of intracellular sulfate activation, essential for assimilatory reduction of sulfate to sulfide, involved in methionine metabolism; human homolog PAPSS2 complements yeast null mutant
EFB1	-1.58	Translation elongation factor 1 beta; stimulates nucleotide exchange to regenerate EF-1 alpha-GTP for the next elongation cycle; part of the EF-1 complex, which facilitates binding of aminoacyl-tRNA to the ribosomal A site; human homolog EEF1B2 can complement yeast efb1 mutants
APC1	-1.59	Largest subunit of the Anaphase-Promoting Complex/Cyclosome; APC/C is a ubiquitin-protein ligase required for degradation of anaphase inhibitors, including mitotic cyclins, during the metaphase/anaphase transition; component of the platform domain of the APC/C, based on structural analysis; localizes to nuclear foci that become diffuse upon DNA replication stress
YDL016C	-1.59	Dubious open reading frame; unlikely to encode a functional protein, based on available experimental and comparative sequence data; partially overlaps the verified ORF CDC7/YDL153C, the catalytic subunit of a complex that regulates DNA replication
PEP8	-1.60	Vacuolar protein component of the retromer; forms part of the multimeric membrane-associated retromer complex involved in vacuolar protein sorting along with Vps35p, Vps29p, Vps17p, and Vps5p; essential for endosome-to-Golgi retrograde protein transport; interacts with Ypt7p; protein abundance increases in response to DNA replication stress
YPT32	-1.60	Rab family GTPase involved in the exocytic pathway; mediates intra-Golgi traffic or the budding of post-Golgi vesicles from the trans-Golgi; protein abundance increases in response to DNA replication stress; YPT32 has a paralog, YPT31, that arose from the whole genome duplication
PEX32	-1.61	Peroxisomal integral membrane protein; involved in negative regulation of peroxisome size; partially functionally redundant with Pex31p; genetic interactions suggest action at a step downstream of steps mediated by Pex28p and Pex29p
UTR4	-1.61	Protein with sequence similarity to acireductone synthases; involved in methionine salvage; found in both the cytoplasm and nucleus
MRP21	-1.61	Mitochondrial ribosomal protein of the small subunit; MRP21 exhibits genetic interactions with mutations in the COX2 and COX3 mRNA 5'-untranslated leader sequences
BUD14	-1.61	Protein involved in bud-site selection; Bud14p-Glc7p complex is a cortical regulator of dynein; forms a complex with Kel1p and Kel2p that regulates Bnr1p (formin) to affect actin cable assembly, cytokinesis, and polarized growth; diploid mutants display a random budding pattern instead of the wild-type bipolar pattern; relative distribution to the nucleus increases upon DNA replication stress
YAR070C	-1.61	Dubious open reading frame; unlikely to encode a protein, based on available experimental and comparative sequence data; YAR070C has a paralog, YHR214C-B, that arose from a segmental duplication
ECM13	-1.62	Non-essential protein of unknown function; induced by treatment with 8-methoxypsoralen and UVA irradiation; ECM13 has a paralog, YJR115W, that arose from the whole genome duplication
EMI2	-1.62	Hexokinase expressed during growth with low glucose levels; induced on non-fermentable carbon sources; required for transcriptional induction of the early meiotic-specific transcription factor IME1; required for sporulation; expression regulated by glucose-repression transcription factors Mig1/2p; protein abundance increases in response to DNA replication stress; EMI2 has a paralog, GLK1, that arose from the whole genome duplication
YBL113W-A	-1.62	Dubious open reading frame; unlikely to encode a functional protein, based on available experimental and comparative sequence data; identified by gene-trapping, microarray-based expression analysis, and genome-wide homology searching
MAD2	-1.62	Component of the spindle-assembly checkpoint complex; delays onset of anaphase in cells with defects in mitotic spindle assembly; forms a complex with Mad1p; regulates APC/C activity during prometaphase and metaphase of meiosis I; gene dosage imbalance between MAD1 and MAD2 leads to chromosome instability
CDS1	-1.62	Phosphatidate cytidylyltransferase (CDP-diglyceride synthetase); an enzyme that catalyzes that conversion of CTP + phosphate into diphosphate + CDP-diaclglyerol, a critical step in the synthesis of all major yeast phospholipids; human homolog CDS1 can complement yeast cds1 null mutant
CDC19	-1.63	Pyruvate kinase; functions as a homotetramer in glycolysis to convert phosphoenolpyruvate to pyruvate, the input for aerobic (TCA cycle) or anaerobic (glucose fermentation) respiration; regulated via allosteric activation by fructose bisphosphate; CDC19 has a paralog, PYK2, that arose from the whole genome duplication
NHA1	-1.63	Na+/H+ antiporter; involved in sodium and potassium efflux through the plasma membrane; required for alkali cation tolerance at acidic pH
YAL069W	-1.64	Dubious open reading frame; unlikely to encode a functional protein, based on available experimental and comparative sequence data
AI1	-1.64	Reverse transcriptase required for splicing of the COX1 pre-mRNA; encoded by a mobile group II intron within the mitochondrial COX1 gene
ENB1	-1.64	Ferric enterobactin transmembrane transporter; expressed under conditions of iron deprivation; member of the major facilitator superfamily; expression is regulated by Rcs1p and affected by chloroquine treatment
HRT3	-1.64	Putative SCF-ubiquitin ligase F-box protein; based on both genetic and physical interactions and sequence similarity; identified in association with Cdc53p, Skp1p and Ubi4 in large and small-scale studies
Deleted	-1.66	
BZZ1	-1.66	SH3 domain protein implicated in regulating actin polymerization; able to recruit actin polymerization machinery through its SH3 domains; colocalizes with cortical actin patches and Las17p; interacts with type I myosins
TDH2	-1.66	Glyceraldehyde-3-phosphate dehydrogenase (GAPDH), isozyme 2 y 3; involved in glycolysis and gluconeogenesis; tetramer that catalyzes the reaction of glyceraldehyde-3-phosphate to 1,3 bis-phosphoglycerate; detected in the cytoplasm and cell wall; GAPDH-derived antimicrobial peptides secreted by S. cerevisiae are active against a wide variety of wine-related yeasts and bacteria; binds AU-rich RNA
RSC1	-1.67	Component of the RSC chromatin remodeling complex; required for expression of mid-late sporulation-specific genes; contains two essential bromodomains, a bromo-adjacent homology (BAH) domain, and an AT hook; RSC1 has a paralog, RSC2, that arose from the whole genome duplication
BCK1	-1.67	MAPKKK acting in the protein kinase C signaling pathway; the kinase C signaling pathway controls cell integrity; upon activation by Pkc1p phosphorylates downstream kinases Mkk1p and Mkk2p; MAPKKK is an acronym for mitogen-activated protein (MAP) kinase kinase kinase
RCO1	-1.67	Essential component of the Rpd3S histone deacetylase complex; interacts with Eaf3p
RTT101	-1.67	Cullin subunit of a Roc1p-dependent E3 ubiquitin ligase complex; role in anaphase progression; Rtt101p-Mms22p ligase associates with replisome complex during S phase via Ctf4p; required for recovery after DSB repair; implicated in Mms22-dependent DNA repair; involved with Mms1p in nonfunctional rRNA decay; modified by the ubiquitin-like protein, Rub1p
UTP18	-1.68	Small-subunit processome protein involved in pre-18S rRNA maturation; part of a subunit of the 90S preribosomal particle capable of interacting directly with the 5' ETS of the 35S pre-rRNA; contains WD40 repeats
KEX1	-1.68	Cell death protease essential for hypochlorite-induced apoptosis; involved in the processing of killer toxin and alpha factor precursor; cleaves Lys and Arg residues from the C-terminus of peptides and proteins
TFG1	-1.68	TFIIF (Transcription Factor II) largest subunit; involved in both transcription initiation and elongation of RNA polymerase II; homologous to human RAP74
RIM11	-1.68	Protein kinase; required for signal transduction during entry into meiosis; promotes the formation of the Ime1p-Ume6p complex by phosphorylating Ime1p and Ume6p; shares similarity with mammalian glycogen synthase kinase 3-beta; protein abundance increases in response to DNA replication stress; RIM11 has a paralog, MRK1, that arose from the whole genome duplication
PDS1	-1.68	Securin; inhibits anaphase by binding separin Esp1p; blocks cyclin destruction and mitotic exit, essential for meiotic progression and mitotic cell cycle arrest; localization is cell-cycle dependent and regulated by Cdc28p phosphorylation
PRY3	-1.69	GPI-anchored cell wall protein involved in export of sterols; redundant role with PRY1 and PRY2 in the export of free fatty acids; role in mating efficiency; expression of full-length transcript is daughter cell-specific; in response to alpha factor, a short transcript starting at +452 is expressed and the long form is repressed by Ste12p; member of the CAP protein superfamily (cysteine-rich secretory proteins (CRISP), antigen 5, and pathogenesis related 1 proteins)
SAE3	-1.69	Meiosis-specific protein involved in meiotic recombination; involved in DMC1-dependent meiotic recombination; forms heterodimer with Mei5p; proposed to be an assembly factor for Dmc1p
YGR045C	-1.69	Putative protein of unknown function; conserved across S. cerevisiae strains
YBL081W	-1.69	Non-essential protein of unknown function; null mutation results in a decrease in plasma membrane electron transport
MLC1	-1.70	Essential light chain for Myo1p; light chain for Myo2p; stabilizes Myo2p by binding to the neck region; interacts with Myo1p, Iqg1p, and Myo2p to coordinate formation and contraction of the actomyosin ring with targeted membrane deposition
SPR3	-1.70	Sporulation-specific homolog of the CDC3/10/11/12 family of genes; septin protein involved in sporulation; regulated by ABFI; the yeast CDC3/10/11/12 family is a family of bud neck microfilament genes
KCC4	-1.70	Protein kinase of the bud neck involved in the septin checkpoint; associates with septin proteins, negatively regulates Swe1p by phosphorylation, shows structural homology to bud neck kinases Gin4p and Hsl1p; KCC4 has a paralog, GIN4, that arose from the whole genome duplication
YGR067C	-1.71	Putative protein of unknown function; contains a zinc finger motif similar to that of Adr1p
YHR177W	-1.71	Putative transcription factor containing a WOPR domain; binds DNA in vitro; similar to C. albicans Wor1p transcription factor that regulates white-opaque switching; overexpression causes a cell cycle delay or arrest
YGL007W	-1.71	Putative protein of unknown function; conserved among S. cerevisiae strains; located in the upstream region of PMA1; deletion leads to polyamine resistance due to downregulation of PMA1
LDH1	-1.71	Serine hydrolase; exhibits active esterase plus weak triacylglycerol lipase activities; proposed role in lipid homeostasis, regulating phospholipid and non-polar lipid levels and required for mobilization of LD-stored lipids; localizes to the lipid droplet (LD) surface; contains a classical serine containing catalytic triad (GxSxG motif)
YNR066C	-1.71	Putative membrane-localized protein of unknown function
YDL086W	-1.72	Putative carboxymethylenebutenolidase; the authentic, non-tagged protein is detected in highly purified mitochondria in high-throughput studies; YDL086W is not an essential gene
DRS2	-1.72	Trans-golgi network aminophospholipid translocase (flippase); P4-type ATPase; maintains membrane lipid asymmetry in post-Golgi secretory vesicles; contributes to clathrin-coated vesicle formation, endocytosis, protein trafficking between the Golgi and endosomal system and the cellular response to mating pheromone; autoinhibited by its C-terminal tail; localizes to the trans-Golgi network
ECM32	-1.72	DNA dependent ATPase/DNA helicase; helicase belonging to the Dna2p- and Nam7p-like family of helicases that is involved in modulating translation termination; interacts with the translation termination factors, localized to polysomes
RPL16A	-1.72	Ribosomal 60S subunit protein L16A; N-terminally acetylated, binds 5.8 S rRNA; transcriptionally regulated by Rap1p; homologous to mammalian ribosomal protein L13A and bacterial L13; RPL16A has a paralog, RPL16B, that arose from the whole genome duplication; protein abundance increases in response to DNA replication stress
YJL068C	-1.73	Esterase that can function as an S-formylglutathione hydrolase; non-essential intracellular esterase; may be involved in the detoxification of formaldehyde, which can be metabolized to S-formylglutathione; similar to human esterase D
YOR1	-1.73	Plasma membrane ATP-binding cassette (ABC) transporter; multidrug transporter mediates export of many different organic anions including oligomycin; homolog of human cystic fibrosis transmembrane receptor (CFTR)
YPR091C	-1.74	Lipid-binding ER protein, enriched at nucleus-vacuolar junctions (NVJ); involved in nonvesicular transfer of ceramides from ER to Golgi; may be involved in sterol metabolism or signaling at the NVJ; contains a synaptotagmin-like-mitochondrial-lipid binding protein (SMP) domain; binds phosphatidylinositols and other lipids in a large-scale study; may interact with ribosomes, based on co-purification experiments
SSK22	-1.74	MAP kinase kinase kinase of HOG1 mitogen-activated signaling pathway; functionally redundant with Ssk2p; interacts with and is activated by Ssk1p; phosphorylates Pbs2p; SSK22 has a paralog, SSK2, that arose from the whole genome duplication
TED1	-1.74	GPI-glycan remodelase; conserved phosphoesterase domain-containing protein; acts together with Emp24p/Erv25p in cargo exit from the ER; functional ortholog of mammalian GPI-glycan remodelase PGAP5; deletion confers sensitivity to 4-(N-(S-glutathionylacetyl)amino) phenylarsenoxide (GSAO)
CTK2	-1.74	Beta subunit of C-terminal domain kinase I (CTDK-I); which phosphorylates both RNA pol II subunit Rpo21p to affect transcription and pre-mRNA 3' end processing, and ribosomal protein Rps2p to increase translational fidelity; relocalizes to the cytosol in response to hypoxia
YGR058W	-1.74	Penta-EF-hand protein; required for polar bud growth and cell wall abscission; binds calcium and zinc with different affinity; localizes to bud site in G1, bud neck in G2; binds to Sec31p and modulates COPII coat assembly
MPS3	-1.75	Nuclear envelope protein; required for SPB insertion, SPB duplication, Kar5p localization near the SPB and nuclear fusion; interacts with Mps2p to tether half-bridge to core SPB; N-terminal acetylation by Eco1p regulates its role in nuclear organization; localizes to the SPB half bridge and telomeres during meiosis; required with Ndj1p and Csm4p for meiotic bouquet formation and telomere-led rapid prophase movement; member of the SUN protein family (Sad1-UNC-84 homology)
YJL022W	-1.76	Dubious open reading frame; unlikely to encode a functional protein, based on available experimental and comparative sequence data; partially overlaps the verified gene PET130
TKL2	-1.76	Transketolase; catalyzes conversion of xylulose-5-phosphate and ribose-5-phosphate to sedoheptulose-7-phosphate and glyceraldehyde-3-phosphate in the pentose phosphate pathway; needed for synthesis of aromatic amino acids; TKL2 has a paralog, TKL1, that arose from the whole genome duplication
YFL032W	-1.76	Dubious open reading frame; unlikely to encode a functional protein, based on available experimental and comparative sequence data; partially overlaps the verified gene HAC1/YFL031W; YFL032W is not an essential gene
PSD2	-1.76	Phosphatidylserine decarboxylase of the Golgi and vacuolar membranes; converts phosphatidylserine to phosphatidylethanolamine; controls vacuolar membrane phospholipid content by regulating phospholipids in compartments that will eventually give rise to the vacuole; loss of Psd2p causes a specific reduction in vacuolar membrane PE levels while total PE levels are not significantly affected
SSZ1	-1.77	Hsp70 protein that interacts with Zuo1p (a DnaJ homolog); interacts with Zuo1p to form a ribosome-associated complex that binds the ribosome via the Zuo1p subunit; also involved in pleiotropic drug resistance via sequential activation of PDR1 and PDR5; binds ATP
TAF1	-1.77	TFIID subunit, involved in RNA pol II transcription initiation; possesses in vitro histone acetyltransferase activity but its role in vivo appears to be minor; involved in promoter binding and G1/S progression; relocalizes to the cytosol in response to hypoxia
YDR131C	-1.77	F-box protein subunit of SCF ubiquitin ligase complex; substrate-specific adaptor subunit that recruits substrates to a core ubiquitination complex
CBP6	-1.77	Mitochondrial protein required for translation of the COB mRNA; forms a complex with Cbp3p that binds to mt ribosomes near the polypeptide tunnel exit and promotes efficient translation of the COB mRNA; Cbp3p-Cbp6p complex also interacts with newly synthesized cytochrome b (Cobp) and Cbp4p to promote assembly of Cobp into the cytochrome bc1 complex; Cbp3p-Cbp6p complex is sequestered if assembly of Complex III is blocked, downregulating COB mRNA translation
RPS4B	-1.78	Protein component of the small (40S) ribosomal subunit; homologous to mammalian ribosomal protein S4, no bacterial homolog; RPS4B has a paralog, RPS4A, that arose from the whole genome duplication
LIP2	-1.78	Lipoyl ligase; involved in the modification of mitochondrial enzymes by the attachment of lipoic acid groups
RPS14A	-1.79	Protein component of the small (40S) ribosomal subunit; required for ribosome assembly and 20S pre-rRNA processing; mutations confer cryptopleurine resistance; homologous to mammalian ribosomal protein S14 and bacterial S11; RPS14A has a paralog, RPS14B, that arose from the whole genome duplication
YIM1	-1.79	Aldehyde reductase; involved in detoxification of lignocellulose-derived aldehydes; catalyzes the reduction of furan, aliphatic and aromatic aldehydes using either NADH or NADPH as co-factor; membrane of the quinone oxidoreductase subfamily of the medium-chain dehydrogenase/reductase family; null mutant displays sensitivity to DNA damaging agents; protein abundance increases in response to DNA replication stress
MEC1	-1.79	Genome integrity checkpoint protein and PI kinase superfamily member; Mec1p and Dun1p function in same pathway to regulate dNTP pools and telomere length; signal transducer required for cell cycle arrest and transcriptional responses to damaged or unreplicated DNA; facilitates replication fork progression and regulates P-body formation under replication stress; promotes interhomolog recombination by phosphorylating Hop1p; associates with shortened, dysfunctional telomeres
YHR145C	-1.80	Dubious open reading frame; unlikely to encode a functional protein, based on available experimental and comparative sequence data
EMA17	-1.81	Protein involved in targeting mitochondrial membrane protein precursors to mitochondrial translocation system; deletion confers sensitivity to 4-(N-(S-glutathionylacetyl)amino) phenylarsenoxide (GSAO); YIL029C has a paralog, YPR071W, that arose from a single-locus duplication
BUD5	-1.81	GTP/GDP exchange factor for Rsr1p (Bud1p); required for both axial and bipolar budding patterns; mutants exhibit random budding in all cell types
YDR111C	-1.82	Catalytically inactive alanine transaminase; expression is repressed in the presence of alanine and repression is mediated by Nrg1p; ALT2 has a paralog, ALT1, that arose from the whole genome duplication
AIP1	-1.82	Actin cortical patch component; interacts with the actin depolymerizing factor cofilin; inhibits elongation of aged ADP-actin filaments decorated with cofilin to maintain a high level of assembly-competent actin species; required to restrict cofilin localization to cortical patches; putative regulator of cytokinesis; contains WD repeats; mutations in human homolog WDR1 are associated with cardiac hypertrophy
MMS21	-1.83	Highly conserved SUMO E3 ligase subunit of SMC5-SMC6 complex; required for anchoring dsDNA breaks to the nuclear periphery; SMC5-SMC6 plays a key role in removal of X-shaped DNA structures that arise between sister chromatids during DNA replication and repair; required for efficient sister chromatid cohesion; mutants are sensitive to MMS, show increased spontaneous mutation and mitotic recombination; SUMOylates and inhibits Snf1p function; supports nucleolar function
BUD8	-1.83	Protein involved in bud-site selection; diploid mutants display a unipolar budding pattern instead of the wild-type bipolar pattern, and bud at the proximal pole; BUD8 has a paralog, BUD9, that arose from the whole genome duplication
YGR064W	-1.83	Dubious open reading frame; unlikely to encode a functional protein, based on available experimental and comparative sequence data; partially overlaps the verified ORF SPT4/YGR063C
CYS3	-1.84	Cystathionine gamma-lyase; catalyzes one of the two reactions involved in the transsulfuration pathway that yields cysteine from homocysteine with the intermediary formation of cystathionine; protein abundance increases in response to DNA replication stress
YGL235W	-1.85	Putative protein of unknown function; potential Cdc28p substrate; null mutant displays increased resistance to antifungal agents gliotoxin, cycloheximide and H2O2
SPS1	-1.85	STE20-family GCKIII protein kinase; required for prospore membrane closure, efficient spore packaging, and localization of enzymes involved in spore wall synthesis; component of a non-canonical Hippo pathway with Cdc15p involved in spindle disassembly and sustained release of Cdc14p during anaphase II; forms a complex with Cdc15p; phosphorylation requires Cdc15p; required for Ssp1p phosphorylation and turnover; interacts with 14-3-3 proteins; expressed in late meiosis; nuclear and cytoplasmic
APM2	-1.86	Protein of unknown function; homologous to the medium chain of mammalian clathrin-associated protein complex; involved in vesicular transport
PUG1	-1.86	Plasma membrane protein involved in protoprophyrin and heme transport; roles in the uptake of protoprophyrin IX and the efflux of heme; expression is induced under both low-heme and low-oxygen conditions; member of the fungal lipid-translocating exporter (LTE) family of proteins
HBS1	-1.87	GTPase with similarity to translation release factors; together with binding partner Dom34p, facilitates ribosomal subunit dissociation and peptidyl-tRNA release when translation is stalled, particularly in 3' UTRs; genetically implicated in mRNA no-go decay; HBS1 has a paralog, SKI7, that arose from the whole genome duplication
RRP14	-1.87	Essential protein, constituent of 66S pre-ribosomal particles; interacts with proteins involved in ribosomal biogenesis and cell polarity; member of the SURF-6 family
MSS11	-1.87	Transcription factor; involved in regulation of invasive growth and starch degradation; controls the activation of FLO11 and STA2 in response to nutritional signals; forms a heterodimer with Flo8p that interacts with the Swi/Snf complex during transcriptional activation of FLO1, FLO11, and STA1
DLS1	-1.88	Subunit of ISW2/yCHRAC chromatin accessibility complex; ISW2/yCHRAC also includes Itc1p, Isw2p, and Dpb4p; involved in inheritance of telomeric silencing; DLS1 has a paralog, DPB3, that arose from the whole genome duplication
CDC7	-1.88	S/T kinase and catalytic subunit of DDK (Dbf4-dependent kinase); required for origin firing and replication initiation; phosphorylates MCM subunits and Cdc45 to drive assembly of the pre-IC and the CMG helicase; maintains Cse4p at kinetochores through phosphorylation to regulate chromosome segregation, regulates pre-meiotic replication, meiotic DSB formation, monopolar attachment of homologs during MI through monopolin recruitment and middle meiotic gene expression; HsCDC7 and DBF4 complement
YBL111C	-1.88	Helicase-like protein encoded within the telomeric Y' element; relocalizes from mitochondrion to cytoplasm upon DNA replication stress
YDR117C	-1.89	Protein of unknown function that associates with ribosomes; has a putative RNA binding domain; in mammals the corresponding protein, eIF2D, has been shown to possess translation initiation factor activity
FAB1	-1.89	1-phosphatidylinositol-3-phosphate 5-kinase; vacuolar membrane kinase that generates phosphatidylinositol (3,5)P2, which is involved in vacuolar sorting and homeostasis

YAL042C-A	-1.89	Dubious open reading frame; unlikely to encode a functional protein, based on available experimental and comparative sequence data; partially overlaps verified ORF ERV46/YAL042W; YAL042C-A is a non-essential gene
RPT4	-1.90	ATPase of the 19S regulatory particle of the 26S proteasome; one of six ATPases of the regulatory particle; involved in degradation of ubiquitinated substrates; contributes preferentially to ERAD; required for spindle pole body duplication; mainly nuclear localization
DPB4	-1.91	Subunit of DNA pol epsilon and of ISW2 chromatin accessibility complex; involved in both chromosomal DNA replication and inheritance of telomeric silencing; stabilizes the interaction of Pol epsilon with primer-template DNA, positively affecting the processivity of the polymerase and exonuclease activities of Pol epsilon; interacts with extranucleosomal DNA and acts as anchor point for ISW2 complex that retains its position on DNA during nucleosome mobilization
DIA3	-1.91	Protein of unknown function; involved in invasive and pseudohyphal growth
MPM1	-1.92	Mitochondrial intermembrane space protein of unknown function
BSD2	-1.93	Heavy metal ion homeostasis protein; facilitates trafficking of Smf1p and Smf2p metal transporters to vacuole where they are degraded; acts as an adaptor protein with Rsp5p in the regulated endocytosis of Smf1p and is itself ubiquitylated by Rsp5p; controls metal ion transport, prevents metal hyperaccumulation, functions in copper detoxification
WHI3	-1.93	RNA binding protein that modulates mRNA stability; regulates cell cycle, sister chromatid cohesion, and stress response genes; modulates stability and translational efficiency of CLN3 mRNA; colocalizes with P-bodies/stress granules to regulate target mRNAs; regulates cell fate and critical cell size for Start passage; regulates ploidy; self-templating mnemon that forms inactive super-assemblies, preventing G1 arrest in mother cells after unsuccessful mating and causing sterility in old cells
Q0032	-1.93	Dubious open reading frame; unlikely to encode a functional protein, based on available experimental and comparative sequence data
GYP6	-1.93	GTPase-activating protein (GAP) for yeast Rab family member Ypt6p; involved in vesicle mediated protein transport
FYV6	-1.93	Protein of unknown function; required for survival upon exposure to K1 killer toxin; proposed to regulate double-strand break repair via non-homologous end-joining
RUB1	-1.93	Ubiquitin-like protein with similarity to mammalian NEDD8; conjugation (neddylation) substrates include the cullins Cdc53p, Rtt101p, and Cul3p; activated by Ula1p and Uba3p (E1 enzyme pair); conjugation mediated by Ubc12p (E2 enzyme)
YAP5	-1.94	Basic leucine zipper (bZIP) iron-sensing transcription factor; senses high-iron conditions via two Fe/S clusters bound to its activator domain; involved in diauxic shift; YAP5 has a paralog, YAP7, that arose from the whole genome duplication
RIX1	-1.94	Component of the Rix1 complex and possibly pre-replicative complexes; required for processing of ITS2 sequences from 35S pre-rRNA; component of the pre-60S ribosomal particle with the dynein-related AAA-type ATPase Mdn1p; required for pre-replicative complex (pre-RC) formation and maintenance during DNA replication licensing; relocalizes to the cytosol in response to hypoxia; essential gene
HEF3	-1.94	Translational elongation factor EF-3; member of the ABC superfamily; stimulates EF-1 alpha-dependent binding of aminoacyl-tRNA by the ribosome; normally expressed in zinc deficient cells; HEF3 has a paralog, YEF3, that arose from the whole genome duplication
AAC3	-1.95	Mitochondrial inner membrane ADP/ATP translocator; exchanges cytosolic ADP for mitochondrially synthesized ATP; expressed under anaerobic conditions; similar to Aac1p; has roles in maintenance of viability and in respiration; AAC3 has a paralog, PET9, that arose from the whole genome duplication
SPO16	-1.96	Meiosis-specific protein involved in synaptonemal complex assembly; implicated in regulation of crossover formation; required for sporulation
SKT5	-1.97	ctivator of Chs3p (chitin synthase III) during vegetative growth; recruits Chs3p to the bud neck via interaction with Bni4p; SKT5 has a paralog, SHC1, that arose from the whole genome duplication
JEM1	-1.98	DnaJ-like chaperone required for nuclear membrane fusion during mating; localizes to the ER membrane; exhibits genetic interactions with KAR2
YAR029W	-1.99	Member of DUP240 gene family but contains no transmembrane domains; green fluorescent protein (GFP)-fusion protein localizes to the cytoplasm in a punctate pattern
RPL19A	-1.99	Ribosomal 60S subunit protein L19A-B; rpl19a and rpl19b single null mutations result in slow growth, while the double null mutation is lethal; homologous to mammalian ribosomal protein L19, no bacterial homolog; RPL19A has a paralog, RPL19B, that arose from the whole genome duplication
SPP381	-2.00	mRNA splicing factor, component of U4/U6.U5 tri-snRNP; interacts genetically and physically with Prp38p; relocalizes to the cytosol in response to hypoxia; temperature-sensitive phenotype of prp38-1 mutant can be suppressed by human homolog of SPP381, MFAP1
KIN82	-2.01	Putative serine/threonine protein kinase; implicated in the regulation of phospholipid asymmetry through the activation of phospholipid translocases (flippases); involved in the phosphorylation of upstream inhibitory kinase Ypk1p along with Fpk1p; has a redundant role in the cellular response to mating pheromone; KIN82 has a paralog, FPK1, that arose from the whole genome duplication
BOL3	-2.01	Protein involved in Fe-S cluster transfer to mitochondrial clients; protects [4Fe-4S] clusters from damage due to oxidative stress by acting along with Nfu1p at a late step in the transfer of [4Fe-4S] clusters from the ISA complex to mitochondrial client proteins like lipoate synthase and succinate dehydrogenase; sequence similarity to human BOLA family member, BOLA3, mutations of which are associated with Multiple Mitochondria Dysfunctions Syndrome (MMDS2)
NDE1	-2.03	Mitochondrial external NADH dehydrogenase; type II NAD(P)H:quinone oxidoreductase that catalyzes the oxidation of cytosolic NADH; Nde1p and Nde2p provide cytosolic NADH to the mitochondrial respiratory chain; NDE1 has a paralog, NDE2, that arose from the whole genome duplication
KIN3	-2.04	Nonessential serine/threonine protein kinase; possible role in DNA damage response; influences tolerance to high levels of ethanol
AI2	-2.04	Reverse transcriptase required for splicing of the COX1 pre-mRNA; encoded by a mobile group II intron within the mitochondrial COX1 gene
OSH2	-2.05	Member of an oxysterol-binding protein family with seven members; in S. cerevisiae, family members have overlapping, redundant functions in sterol metabolism and collectively perform a function essential for viability; contains FFAT motif; interacts with ER anchor Scs2p at patches at the plasma membrane and at the nuclear envelope; regulated by sterol binding; OSH2 has a paralog, SWH1, that arose from the whole genome duplication
YEL008W	-2.06	Putative protein of unknown function; conserved among S. cerevisiae strains; YEL008W is not an essential gene; predicted to be involved in metabolism
CCR4	-2.07	Component of the CCR4-NOT transcriptional complex; CCR4-NOT is involved in regulation of gene expression; component of the major cytoplasmic deadenylase, which is involved in mRNA poly(A) tail shortening
RNP1	-2.07	Ribonucleoprotein that contains two RNA recognition motifs (RRM); RNP1 has a paralog, SBP1, that arose from the whole genome duplication
TEA1	-2.07	Ty1 enhancer activator involved in Ty enhancer-mediated transcription; required for full levels of Ty enhancer-mediated transcription; C6 zinc cluster DNA-binding protein
NPP2	-2.08	Nucleotide pyrophosphatase/phosphodiesterase; mediates extracellular nucleotide phosphate hydrolysis along with Npp1p and Pho5p; activity and expression enhanced during conditions of phosphate starvation; involved in spore wall assembly; SWAT-GFP and mCherry fusion proteins localize to the endoplasmic reticulum; NPP2 has a paralog, NPP1, that arose from the whole genome duplication; npp1 npp2 double mutant exhibits reduced dityrosine fluorescence relative to single mutants
SDP1	-2.08	Stress-inducible dual-specificity MAP kinase phosphatase; negatively regulates Slt2p MAP kinase by direct dephosphorylation, diffuse localization under normal conditions shifts to punctate localization after heat shock; SDP1 has a paralog, MSG5, that arose from the whole genome duplication
RPS16A	-2.08	Protein component of the small (40S) ribosomal subunit; homologous to mammalian ribosomal protein S16 and bacterial S9; RPS16A has a paralog, RPS16B, that arose from the whole genome duplication
ADE12	-2.08	Adenylosuccinate synthase; catalyzes the first step in synthesis of adenosine monophosphate from inosine 5'monophosphate during purine nucleotide biosynthesis; exhibits binding to single-stranded autonomously replicating (ARS) core sequence
GCN4	-2.08	bZIP transcriptional activator of amino acid biosynthetic genes; activator responds to amino acid starvation; expression is tightly regulated at both the transcriptional and translational levels; contains four upstream open reading frames (uORFs) in 5' untranslated region which regulate translation
YHR217C	-2.09	Dubious open reading frame; unlikely to encode a functional protein, based on available experimental and comparative sequence data; located in the telomeric region TEL08R
RRF1	-2.09	Mitochondrial ribosome recycling factor; essential for mitochondrial protein synthesis and for the maintenance of the respiratory function of mitochondri
SET5	-2.09	Methyltransferase involved in methylation of histone H4 Lys5, -8, -12; S-adenosylmethionine-dependent; zinc-finger protein, contains one canonical and two unusual fingers in unusual arrangements; deletion enhances replication of positive-strand RNA virus
PRM2	-2.10	Pheromone-regulated protein; predicted to have 4 transmembrane segments and a coiled coil domain; regulated by Ste12p; required for efficient nuclear fusion
IRC4	-2.10	Protein similar to bacterial secreted DfsB proteins; null mutant displays increased levels of spontaneous Rad52p foci; green fluorescent protein (GFP)-fusion protein localizes to the cytoplasm and nucleus
MPC1	-2.10	Highly conserved subunit of mitochondrial pyruvate carrier (MPC); MPC is a mitochondrial inner membrane complex that mediates pyruvate uptake and comprises Mpc1p and Mpc2p during fermentative growth, or Mcp1p and Mpc3p during respiratory growth; null mutant displays slow growth that is complemented by expression of human or mouse ortholog; mutation in human ortholog MPC1 is associated with lactic acidosis and hyperpyruvatemia
ASA1	-2.11	Subunit of the ASTRA complex, involved in chromatin remodeling; telomere length regulator involved in the stability or biogenesis of PIKKs such as TORC1
BI2	-2.11	Mitochondrial mRNA maturase with a role in splicing; encoded by both exon and intron sequences of partially processed COB mRNA
CPR2	-2.11	Peptidyl-prolyl cis-trans isomerase (cyclophilin); catalyzes the cis-trans isomerization of peptide bonds N-terminal to proline residues; potential role in the secretory pathway; seamless-GFP and mCherry fusion proteins localize to the vacuole, while SWAT-GFP fusion localizes to both the endoplasmic reticulum and vacuole; suppresses toxicity of slow-folding human Z-type alpha1-antitrypsin variant associated with liver cirrhosis and emphysema
YAL034C-B	-2.12	Dubious open reading frame; unlikely to encode a functional protein, based on available experimental and comparative sequence data
CYC3	-2.12	Cytochrome c heme lyase (holocytochrome c synthase); attaches heme to apo-cytochrome c (Cyc1p or Cyc7p) in mitochondrial intermembrane space; human homolog HCCS implicated in microphthalmia with linear skin defects (MLS), and can complement yeast null mutant
APD1	-2.12	2Fe-2S cytosolic protein of unknown function; bishistidinyl coordinated, non-Rieske [2Fe-2S] cluster containing thiredoxin-like ferredoxin; required for normal localization of actin patches and for normal tolerance of sodium ions and hydrogen peroxide; green fluorescent protein (GFP)-fusion protein localizes to both the nucleus and the cytoplasm; homologous to AIM32
SPT20	-2.13	Subunit of the SAGA transcriptional regulatory complex; involved in maintaining the integrity of the complex; mutant displays reduced transcription elongation in the G-less-based run-on (GLRO) assay
YJL045W	-2.14	Minor succinate dehydrogenase isozyme; participates in oxidation of succinate and transfer of electrons to ubiquinone; induced during the diauxic shift in a Cat8p-dependent manner; YJL045W has a paralog, SDH1, that arose from the whole genome duplication
URM1	-2.15	Ubiquitin-like protein involved in thiolation of cytoplasmic tRNAs; receives sulfur from the E1-like enzyme Uba4p and transfers it to tRNA; also functions as a protein tag with roles in nutrient sensing and oxidative stress response
RPL7A	-2.15	Ribosomal 60S subunit protein L7A-B; required for processing of 27SA3 pre-rRNA to 27SB pre-rRNA during assembly of large ribosomal subunit; depletion leads to a turnover of pre-rRNA; contains a conserved C-terminal Nucleic acid Binding Domain (NDB2); binds to Domain II of 25S and 5.8S rRNAs; homologous to mammalian ribosomal protein L7 and bacterial L30; RPL7B has a paralog, RPL7A, that arose from the whole genome duplication
ILS1	-2.16	Cytoplasmic isoleucine-tRNA synthetase; target of the G1-specific inhibitor reveromycin A
ALG1	-2.16	Mannosyltransferase; involved in asparagine-linked glycosylation in the endoplasmic reticulum (ER); essential for viability; human homolog ALG1 complements yeast null mutant
CLN3	-2.17	G1 cyclin involved in cell cycle progression; activates Cdc28p kinase to promote G1 to S phase transition; plays a role in regulating transcription of other G1 cyclins, CLN1 and CLN2; regulated by phosphorylation and proteolysis; acetyl-CoA induces CLN3 transcription in response to nutrient repletion to promote cell-cycle entry; cell cycle arrest phenotype of the cln1 cln2 cln3 triple null mutant is complemented by any of human cyclins CCNA2, CCNB1, CCNC, CCND1, or CCNE1
NIS1	-2.17	Protein involved in axial bud site selection; prevents repolarization of cells at previous division sites; recruited with Nba1p to the cell cortex and to cytokinesis remnants (bud scars) by Aim44p and Nap1p where Nba1p prevent Rsr1p-mediated activation of Cdc24p, and therefore Cdc42p activation; acts with Nba1p to establish transient bud scar localization of Rga1p, a Cdc42p GAP; localizes to the nucleus and to cell division site during G2/M, and is then stably recruited to cytokinesis remnants
RPL16B	-2.19	Ribosomal 60S subunit protein L16B; N-terminally acetylated, binds 5.8 S rRNA; transcriptionally regulated by Rap1p; homologous to mammalian ribosomal protein L13A and bacterial L13; RPL16B has a paralog, RPL16A, that arose from the whole genome duplication
ERV46	-2.19	Protein localized to COPII-coated vesicles; forms a complex with Erv41p; involved in the membrane fusion stage of transport
UBA4	-2.20	E1-like protein that activates Urm1p before urmylation; also acts in thiolation of the wobble base of cytoplasmic tRNAs by adenylating and then thiolating Urm1p; receives sulfur from Tum1p
EBP2	-2.20	Required for 25S rRNA maturation and 60S ribosomal subunit assembly; localizes to the nucleolus and in foci along nuclear periphery; constituent of 66S pre-ribosomal particles; cooperates with Rrs1p and Mps3p to mediate telomere clustering by binding Sir4p, but is not involved in telomere tethering
UBC11	-2.20	Ubiquitin-conjugating enzyme; most similar in sequence to Xenopus ubiquitin-conjugating enzyme E2-C, but not a true functional homolog of this E2; unlike E2-C, not required for the degradation of mitotic cyclin Clb2
DNF3	-2.21	Trans-golgi network aminophospholipid translocase (flippase); type 4 P-type ATPase; involved in phospholipid translocation, contributing to the maintenance of membrane lipid asymmetry in post-Golgi secretory vesicles; role in protein trafficking between the Golgi and endosomal system; localizes to the trans-Golgi network; localizes to the shmoo tip where it has a redundant role in the cellular response to mating pheromone
YAL037W	-2.21	Putative protein of unknown function; YAL037W has a paralog, YOR342C, that arose from the whole genome duplication
TEF2	-2.22	Translational elongation factor EF-1 alpha; GTP-bound active form, binds to and delivers aminoacylated tRNA to the A-site of ribosomes for elongation of nascent polypeptides; moonlighting function as an actin binding and bundling protein; association with GTPase Rho1p on the vacuolar membrane may facilitate F-actin remodeling; involved in tRNA re-export from the nucleus; Tef2p-RFP levels increase during replicative aging
ARG2	-2.23	Acetylglutamate synthase (glutamate N-acetyltransferase); mitochondrial enzyme that catalyzes the first step in the biosynthesis of the arginine precursor ornithine; forms a complex with Arg5,6p
TOK1	-2.24	Outward-rectifier potassium channel of the plasma membrane; has two pore domains in tandem, each of which forms a functional channel permeable to potassium; carboxy tail functions to prevent inner gate closures; target of K1 toxin
GWT1	-2.25	Protein involved in the inositol acylation of GlcN-PI; the inositol acylation of glucosaminyl phosphatidylinositol (GlcN-PI) forms glucosaminyl(acyl)phosphatidylinositol (GlcN(acyl)PI), an intermediate in the biosynthesis of glycosylphosphatidylinositol (GPI) anchors
YDR066C	-2.26	Protein of unknown function; exhibits genetic interactions with Rtr1p; green fluorescent protein (GFP)-fusion protein localizes to the cytoplasm; YDR066C is not an essential gene; relocalizes from nucleus to cytoplasmic foci upon DNA replication stress; RTR2 has a paralog, RTR1, that arose from the whole genome duplication
VMA22	-2.26	Protein that is required for vacuolar H+-ATPase (V-ATPase) function; peripheral membrane protein; not an actual component of the V-ATPase complex; functions in the assembly of the V-ATPase; localized to the yeast endoplasmic reticulum (ER)
MUB1	-2.27	MYND domain-containing protein; component of the Mub1p-Ubr2p-Rad6p ubiquitin ligase complex, required for ubiquitination and degradation of Rpn4p; interacts with Ubr2p (E3) and indirectly with Rad6p (E2); short-lived protein degraded in a Ubr2p/Rad6p dependent manner; proposed to function as both a partner and substrate of the Ubr2p/Rad6p ubiquitin ligase; similar to the A. nidulans samB gene
GRX3	-2.28	Glutathione-dependent oxidoreductase; hydroperoxide and superoxide-radical responsive monothiol glutaredoxin subfamily member with Grx4p and Grx5p; redundantly protects cells from oxidative damage along with GRX4 and GRX5; with Grx4p, promotes the dissociation of Aft1p from iron regulon gene promoters and subsequent nuclear export in iron-replete conditions, regulating iron homeostasis; involved with Grx4p in the deglutathionylation of Sir2p, restoring deacetylase activity after disulfide stress
YML018C	-2.28	Protein of unknown function; green fluorescent protein (GFP)-fusion protein localizes to the membrane of the vacuole; physical interaction with Atg27p suggests a possible role in autophagy; YML018C is not an essential gene; relative distribution to the vacuolar membrane decreases upon DNA replication stress; YML018C has a paralog, THI74, that arose from the whole genome duplication
APT2	-2.28	Potential adenine phosphoribosyltransferase; encodes a protein with similarity to adenine phosphoribosyltransferase, but artificially expressed protein exhibits no enzymatic activity; APT2 has a paralog, APT1, that arose from the whole genome duplication
TEF1	-2.28	Translational elongation factor EF-1 alpha; GTP-bound active form, binds to and delivers aminoacylated tRNA to the A-site of ribosomes for elongation of nascent polypeptides; moonlighting function as an actin binding and bundling protein; association with GTPase Rho1p on the vacuolar membrane may facilitate F-actin remodeling; involved in tRNA re-export from the nucleus; Tef2p-RFP levels increase during replicative aging
DPH6	-2.29	Diphthamide synthetase; catalyzes the last amidation step of diphthamide biosynthesis using ammonium and ATP; evolutionarily conserved in eukaryotes; dph6 mutants exhibit diphthine accumulation and resistance to sordarin, which is indicative of defects in diphthamide formation on EF2; green fluorescent protein (GFP)-tagged protein localizes to the cytoplasm; DPH6/YLR143W is not an essential gene
AI3	-2.29	Endonuclease I-SceIII; encoded by a mobile group I intron within the mitochondrial COX1 gene
TUS1	-2.29	Guanine nucleotide exchange factor (GEF) that modulates Rho1p activity; involved in the cell integrity signaling pathway; interacts with Rgl1p; localization of Tus1p to the bed neck is regulated by Rgl1p; multicopy suppressor of tor2 mutation and ypk1 ypk2 double mutation; potential Cdc28p substrate
MTG1	-2.30	Putative GTPase peripheral to the mitochondrial inner membrane; essential for respiratory competence, likely functions in assembly of the large ribosomal subunit, has homologs in plants and animals
FLO8	-2.31	Transcription factor; required for flocculation, diploid filamentous growth, and haploid invasive growth; forms a heterodimer with Mss11p that interacts with the Swi/Snf complex during transcriptional activation of FLO1, FLO11, and STA1; S. cerevisiae genome reference strain S288C contains an internal in-frame stop at codon 142, which in other strains encodes tryptophan
VPS8	-2.31	Membrane-binding component of the CORVET complex; involved in endosomal vesicle tethering and fusion in the endosome to vacuole protein targeting pathway; interacts with Vps21p; contains RING finger motif
RIB4	-2.31	Lumazine synthase (DMRL synthase); catalyzes synthesis of immediate precursor to riboflavin; DMRL synthase stands for 6,7-dimethyl-8-ribityllumazine synthase
MAK16	-2.32	Essential nuclear protein; constituent of 66S pre-ribosomal particles; required for maturation of 25S and 5.8S rRNAs; required for maintenance of M1 satellite double-stranded RNA of the L-A virus
MLH1	-2.32	Protein required for mismatch repair in mitosis and meiosis; also required for crossing over during meiosis; forms a complex with Pms1p and Msh2p-Msh3p during mismatch repair; required for silencing at the silent mating-type loci and telomeres; human homolog is associated with hereditary non-polyposis colon cancer
MTL1	-2.33	Putative plasma membrane sensor; involved in cell integrity signaling and stress response during glucose starvation and oxidative stress; has structural and functional similarity to Mid2p; MTL1 has a paralog, MID2, that arose from the whole genome duplication
STU1	-2.37	Microtubule plus-end-tracking non-motor protein; binds to MTs, stabilizing interpolar MT plus ends, facilitating polymerization of spindle MTs and producing an outward force on spindle poles; required for structural integrity of the mitotic spindle; binds detached kinetochores (KTs) and promotes capture; relocalizes to MTs and stabilizes the spindle once captured KTs reach a spindle pole; localizes to the spindle, spindles poles, and detached KTs; CLASP family member homologous to human CLASP1/2
FUN30	-2.42	Snf2p family member with ATP-dependent chromatin remodeling activity; has a role in silencing at the mating type locus, telomeres and centromeres; enriched at centromeres and is required for correct chromatin structure around centromeres, as well as at the boundary element of the silent HMR; recruited to DNA double-strand breaks (DSBs) where it promotes 5' strand resection of DSBs; potential Cdc28p substrate
TDA3	-2.46	Putative oxidoreductase involved in late endosome to Golgi transport; physical and genetical interactions with Btn2p; null mutant is viable, has extended S phase, and sensitive to expression of top1-T722A allele; similar to human FOXRED1
MDR1	-2.47	Cytoplasmic GTPase-activating protein; activates Ypt/Rab transport GTPases Ypt6p, Ypt31p and Sec4p; involved in recycling of internalized proteins and regulation of Golgi secretory function
PEX8	-2.47	Intraperoxisomal organizer of the peroxisomal import machinery; organizes the formation of the importomer complex, bridging the docking complex with the RING finger complex; tightly associated with the lumenal face of the peroxisomal membrane; essential for peroxisome biogenesis; binds PTS1-signal receptor Pex5p, and PTS2-signal receptor Pex7p
RPL13B	-2.49	Ribosomal 60S subunit protein L13B; not essential for viability; homologous to mammalian ribosomal protein L13, no bacterial homolog; RPL13B has a paralog, RPL13A, that arose from the whole genome duplication
BRE1	-2.51	E3 ubiquitin ligase; forms heterodimer with Rad6p to regulate K63 polyubiquitination in response to oxidative stress and to monoubiquinate histone H2B-K123, which is required for the subsequent methylation of histone H3-K4 and H3-K79; required for DSBR, transcription, silencing, and checkpoint control; interacts with RNA-binding protein Npl3p, linking histone ubiquitination to mRNA processing; Bre1p-dependent histone ubiquitination promotes pre-mRNA splicing
ADE17	-2.53	Enzyme of 'de novo' purine biosynthesis; contains both 5-aminoimidazole-4-carboxamide ribonucleotide transformylase and inosine monophosphate cyclohydrolase activities; ADE17 has a paralog, ADE16, that arose from the whole genome duplication; ade16 ade17 mutants require adenine and histidine
SPO13	-2.54	Meiotic regulator; involved in maintaining sister chromatid cohesion during meiosis I as well as promoting proper attachment of kinetochores to the spindle during meiosis I and meiosis II; anaphase-promoting complex (APC) substrate that is degraded during anaphase I; expressed only in meiotic cells
YBL086C	-2.55	Protein of unknown function; green fluorescent protein (GFP)-fusion protein localizes to the cell periphery
TDH1	-1.66	Glyceraldehyde-3-phosphate dehydrogenase (GAPDH), isozyme 1; involved in glycolysis and gluconeogenesis; tetramer that catalyzes the reaction of glyceraldehyde-3-phosphate to 1,3 bis-phosphoglycerate; detected in the cytoplasm and cell wall; protein abundance increases in response to DNA replication stress; GAPDH-derived antimicrobial peptides secreted by S. cerevisiae are active against a wide variety of wine-related yeasts and bacteria
CDC24	-2.61	Guanine nucleotide exchange factor (GEF) for Cdc42p; required for polarity establishment and maintenance, and mutants have morphological defects in bud formation and shmooing; relocalizes from nucleus to cytoplasm upon DNA replication stress; thermosensitivity of the cdc24-4 mutant in the presence of sorbitol is functionally complemented by human CDC42
SPO1	-2.62	Meiosis-specific prospore protein; required for meiotic spindle pole body duplication and separation; required to produce bending force necessary for proper prospore membrane assembly during sporulation; has similarity to phospholipase B
MPS3	-2.72	Nuclear envelope protein; required for SPB insertion, SPB duplication, Kar5p localization near the SPB and nuclear fusion; interacts with Mps2p to tether half-bridge to core SPB; N-terminal acetylation by Eco1p regulates its role in nuclear organization; localizes to the SPB half bridge and telomeres during meiosis; required with Ndj1p and Csm4p for meiotic bouquet formation and telomere-led rapid prophase movement; member of the SUN protein family (Sad1-UNC-84 homology)
PAD1	-2.73	henylacrylic acid decarboxylase; decarboxylates aromatic carboxylic acids to the corresponding vinyl derivatives; confers resistance to cinnamic acid; overexpression of both Pad1p and Fdc1p increases cinnamic acid decarboxylase activity due to the Pad1p-catalyzed formation of a diffusible cofactor required for Fdc1p activity; contains mRNA binding activity; homolog of E. coli UbiX
MF(ALPHA)1	-2.74	Mating pheromone alpha-factor, made by alpha cells; interacts with mating type a cells to induce cell cycle arrest and other responses leading to mating; also encoded by MF(ALPHA)2, although MF(ALPHA)1 produces most alpha-factor; binds copper(II) ions
YBL109W	-2.84	Dubious open reading frame; unlikely to encode a functional protein, based on available experimental and comparative sequence data
BBC1	-2.92	Protein possibly involved in assembly of actin patches; interacts with an actin assembly factor Las17p and with the SH3 domains of Type I myosins Myo3p and Myo5p; localized predominantly to cortical actin patches
NSP1	-2.92	FG-nucleoporin component of central core of the nuclear pore complex; contributes directly to nucleocytoplasmic transport and maintenance of the nuclear pore complex (NPC) permeability barrier; forms a stable association with Nup82p, Gle2p and two other FG-nucleoporins (Nsp1p and Nup159p); NUP116 has a paralog, NUP100, that arose from the whole genome duplication

DPB11	-2.94	DNA replication initiation protein; loads DNA pol epsilon onto pre-replication complexes at origins; checkpoint sensor recruited to stalled replication forks by the checkpoint clamp complex where it activates Mec1p; along with Rfa1p, binds to ultrafine anaphase bridges in mitotic cells and prevents accumulation of chromatin bridges by stimulating the Mec1p kinase and suppressing homologous recombination; ortholog of human TopBP1; forms nuclear foci upon DNA replication stress
KHA1	-2.98	Putative K+/H+ antiporter; involved in intracellular cation homeostasis; promotes copper binding to Fet3p multicopper oxidase; localized to Golgi vesicles and detected in highly purified mitochondria in high-throughput studies
YAL031C	-2.99	Cytoplasmic protein that regulates protein phosphatase 1 Glc7p; protein overexpression relocalizes Glc7p from the nucleus and prevents chromosome segregation; potential Cdc28p substrate
SDT1	-3.05	Pyrimidine nucleotidase; responsible for production of nicotinamide riboside and nicotinic acid riboside; overexpression suppresses the 6-AU sensitivity of transcription elongation factor S-II, as well as resistance to other pyrimidine derivatives; SDT1 has a paralog, PHM8, that arose from the whole genome duplication
RGP1	-3.10	Subunit of a Golgi membrane exchange factor (Ric1p-Rgp1p); this complex catalyzes nucleotide exchange on Ypt6p

Supplemental Table S2. Gene identified of Microarray of 3 dpi from S. cerevisiae

Symbol	Zscore	Function
YHL009W-A	3.39	Retrotransposon TYA Gag gene co-transcribed with TYB Pol; translated as TYA or TYA-TYB polyprotein; Gag is a nucleocapsid protein that is the structural constituent of virus-like particles (VLPs); similar to retroviral Gag
MAM1	3.17	Monopolin; meiosis-specific kinetochore-associated protein involved in monopolar attachment of sister kinetochores to the meiotic spindle; subunit of monopolin, a complex that prevents biorientation of sister kinetochores to ensure homolog biorientation during meiosis I; regulates the conformation, enzyme kinetics and substrate specificity of the Dsn1p kinase, Hrr1p; expressed only during the first meiotic division
GAL3	3.16	Transcriptional regulator; involved in activation of the GAL genes in response to galactose; forms a complex with Gal80p to relieve Gal80p inhibition of Gal4p; binds galactose and ATP but does not have galactokinase activity; GAL3 has a paralog, GAL1, that arose from the whole genome duplication
RRP12	3.11	Protein required for export of the ribosomal subunits; associates with the RNA components of the pre-ribosomes; has a role in nuclear import in association with Pse1p; also plays a role in the cell cycle and the DNA damage response; contains HEAT-repeats
GLG2	3.08	Glycogenin glucosyltransferase; self-glucosylating initiator of glycogen synthesis, also glucosylates n-dodecyl-beta-D-maltoside; similar to mammalian glycogenin; GLG2 has a paralog, GLG1, that arose from the whole genome duplication
REF2	3.02	RNA-binding protein; involved in the cleavage step of mRNA 3'-end formation prior to polyadenylation, and in snoRNA maturation; part of holo-CPF subcomplex APT, which associates with 3'-ends of snoRNA- and mRNA-encoding genes; putative regulatory subunit of type 1 protein phosphatase Glc7p, required for actomyosin ring formation, and for timely dephosphorylation and release of Bnr1p from the division site; relocalizes to the cytosol in response to hypoxia
HAS1	2.97	ATP-dependent RNA helicase; involved in the biogenesis of 40S and 60S ribosome subunits; localizes to both the nuclear periphery and nucleolus; highly enriched in nuclear pore complex fractions; constituent of 66S pre-ribosomal particles
YKU70	2.95	Subunit of the telomeric Ku complex (Yku70p-Yku80p); involved in telomere length maintenance, structure and telomere position effect; required for localization of telomerase ribonucleoprotein to nucleus via interaction with the TLC1 guide RNA; relocates to sites of double-strand cleavage to promote nonhomologous end joining during DSB repair
ATP7	2.92	Subunit d of the stator stalk of mitochondrial F1F0 ATP synthase; F1F0 ATP synthase is a large, evolutionarily conserved enzyme complex required for ATP synthesis
PAC11	2.92	Dynein intermediate chain, microtubule motor protein; required for intracellular transport and cell division; acts in cytoplasmic dynein pathway; forms complex with dynein light chain Dyn2p that promotes Dyn1p homodimerization and potentiates motor processivity; Dyn2p-Pac11p complex is also important for interaction of dynein motor complex with dynactin complex; forms cortical cytoplasmic microtubule capture site with Num1p; essential in the absence of CIN8
SER2	2.87	Phosphoserine phosphatase of the phosphoglycerate pathway; involved in serine and glycine biosynthesis, expression is regulated by the available nitrogen source
MTC1	2.85	Protein of unknown function that may interact with ribosomes; green fluorescent protein (GFP)-fusion protein localizes to the cytoplasm and to COPI-coated vesicles (early Golgi); mtc1 is synthetically lethal with cdc13-1
COS2	2.84	Endosomal protein involved in turnover of plasma membrane proteins; member of the DUP380 subfamily of conserved, often subtelomeric COS genes; required for the multivesicular vesicle body sorting pathway that internalizes plasma membrane proteins for degradation; Cos proteins provide ubiquitin in trans for nonubiquitinated cargo proteins
THI6	2.82	Thiamine-phosphate diphosphorylase and hydroxyethylthiazole kinase; required for thiamine biosynthesis; GFP-fusion protein localizes to the cytoplasm in a punctate pattern
SPT23	2.82	ER membrane protein involved in regulation of OLE1 transcription; inactive ER form dimerizes and one subunit is then activated by ubiquitin/proteasome-dependent processing followed by nuclear targeting; SPT23 has a paralog, MGA2, that arose from the whole genome duplication
CTR1	2.81	High-affinity copper transporter of plasma membrane; mediates nearly all copper uptake under low copper conditions; transcriptionally induced at low copper levels and degraded at high copper levels; protein increases in abundance and relocalizes from nucleus to plasma membrane upon DNA replication stress; human homolog SLC31A1 can complement a yeast ctr1 ctr3 double deletion
YPR148C	2.79	Protein of unknown function; may interact with ribosomes, based on co-purification experiments; green fluorescent protein (GFP)-fusion protein localizes to the cytoplasm in a punctate pattern
UPS3	2.63	Mitochondrial protein of unknown function; similar to Ups1p and Ups2p which are involved in regulation of mitochondrial cardiolipin and phosphatidylethanolamine levels; null is viable but interacts synthetically with ups1 and ups2 mutations; UPS3 has a paralog, UPS2, that arose from the whole genome duplication
YOR389W	2.61	Putative protein of unknown function; expression regulated by copper levels
PHO89	2.56	Plasma membrane Na+/Pi cotransporter; active in early growth phase; similar to phosphate transporters of Neurospora crassa; transcription regulated by inorganic phosphate concentrations and Pho4p; mutations in related human transporter genes hPit1 and hPit2 are associated with hyperphosphatemia-induced calcification of vascular tissue and familial idiopathic basal ganglia calcification
YBR209W	2.53	Putative protein of unknown function; conserved across S. cerevisiae strains; YBR209W is not an essential gene
SBE22	2.50	Protein involved in bud growth; involved in the transport of cell wall components from the Golgi to the cell surface; similar in structure and functionally redundant with Sbe2p; SBE22 has a paralog, SBE2, that arose from the whole genome duplication
MRPL38	2.49	Mitochondrial ribosomal protein of the large subunit; appears as two protein spots (YmL34 and YmL38) on two-dimensional SDS gels; protein abundance increases in response to DNA replication stress
CDC31	2.46	Calcium-binding component of the spindle pole body (SPB) half-bridge; required for SPB duplication in mitosis and meiosis II; homolog of mammalian centrin; binds multiubiquitinated proteins and is involved in proteasomal protein degradation
CKA1	2.46	Alpha catalytic subunit of casein kinase 2 (CK2); a Ser/Thr protein kinase with roles in cell growth and proliferation; CK2, comprised of CKA1, CKA2, CKB1 and CKB2, has many substrates including transcription factors and all RNA polymerases; regulates Fkh1p-mediated donor preference during mating-type switching
SUI1	2.44	Translation initiation factor eIF1; component of complex involved in recognition of initiator codon; modulates translation accuracy at initiation phase by discriminating against suboptimal initiation sites to prevent excessive uORF translation genome-wide
MRPL49	2.42	Mitochondrial ribosomal protein of the large subunit
MME1	2.39	Transporter of the mitochondrial inner membrane that exports magnesium; involved in mitochondrial Mg2+ homeostasis; has similarity to human mitochondrial ATP-Mg/Pi carriers
SML1	2.39	Ribonucleotide reductase inhibitor; involved in regulating dNTP production; regulated by Mec1p and Rad53p during DNA damage and S phase; SML1 has a paralog, DIF1, that arose from the whole genome duplication
ESP1	2.38	Separase/separin, a caspase-like cysteine protease; cleaves Mcd1p/Scc1p, a mitotic cohesin complex subunit, resulting in the dissociation of cohesin from chromatin and sister chromatid separation; cleaves meiotic-cohesin subunit Rec8p along chromosome arms in meiosis I and at centromeric sites during meiosis II; inhibits PP2A-Cdc55p to promote mitotic exit; inhibited by Pds1p (securin); relative distribution to the nucleus increases upon DNA replication stress
SAP155	2.38	Protein required for function of the Sit4p protein phosphatase; forms a complex with Sit4p; member of a family of similar proteins including Sap4p, Sap185p, and Sap190p; protein abundance increases in response to DNA replication stress; SAP155 has a paralog, SAP4, that arose from the whole genome duplication
ATG22	2.38	Vacuolar membrane protein; required for efflux of amino acids during autophagic body breakdown in vacuole; targeted to vacuole via AP-3 pathway; null mutation causes gradual loss of viability during starvation
KAP104	2.37	Transportin or cytosolic karyopherin beta 2; functions in the rg-nuclear localization signal-mediated nuclear import/reimport of mRNA-binding proteins Nab2p and Hrp1p; regulates asymmetric protein synthesis in daughter cells during mitosis
DCP2	2.36	Catalytic subunit of Dcp1p-Dcp2p decapping enzyme complex; removes 5' cap structure from mRNAs prior to their degradation; also enters nucleus and positively regulates transcription initiation; nudix hydrolase family member; forms cytoplasmic foci upon DNA replication stress; human homolog DCP2 complements yeast dcp2 thermosensitive mutant
YMR306C-A	2.33	Dubious open reading frame; unlikely to encode a functional protein, based on available experimental and comparative sequence data; almost completely overlaps 3' end of ORF FKS3/YMR306W
FAF1	2.32	Protein required for pre-rRNA processing; also required for 40S ribosomal subunit assembly
SHE4	2.31	Protein containing a UCS (UNC-45/CRO1/SHE4) domain; binds to myosin motor domains to regulate myosin function; involved in endocytosis, polarization of the actin cytoskeleton, and asymmetric mRNA localization
NIT3	2.30	Nit protein; one of two proteins in S. cerevisiae with similarity to the Nit domain of NitFhit from fly and worm and to the mouse and human Nit protein which interacts with the Fhit tumor suppressor; nitrilase superfamily member
BIM1	2.30	Microtubule plus end-tracking protein; forms a complex with Kar9p that makes up the cortical microtubule capture site and delays the exit from mitosis when the spindle is oriented abnormally; homolog of human end binding protein 1 (EB1)
YEA4	2.30	Uridine diphosphate-N-acetylglucosamine (UDP-GlcNAc) transporter; required for cell wall chitin synthesis; localized to the ER
RAM2	2.28	Alpha subunit of farnesyltransferase and geranylgeranyltransferase-I; farnesyltransferase (Ram2p-Ram1p heterodimer) catalyzes the addition of 15-carbon isoprenoid farnesyl to substrate proteins containing a CAAX consensus motif; type I geranylgeranyltransferase (Ram2p-Cdc43p heterodimer) catalyzes the addition of the 20-carbon isoprenoid geranylgeranyl to substrate proteins containing a CaaL consensus motif; required for membrane localization of Ras proteins and a-factor
UBX4	2.28	UBX domain-containing protein that interacts with Cdc48p; involved in degradation of polyubiquitinated proteins via the ERAD (ER-associated degradation) pathway; modulates the role of the Cdc48p-Nplp-Ufd1p AAA ATPase complex in delivery of misfolded proteins to the proteasome; protein abundance increases in response to DNA replication stress; human ortholog ASPSCR1 partially complements the yeast null mutant
PRM5	2.27	Pheromone-regulated protein, predicted to have 1 transmembrane segment; induced during cell integrity signaling; PRM5 has a paralog, YNL058C, that arose from the whole genome duplication
DDL1	2.26	DDHD domain-containing phospholipase A1; mitochondrial matrix enzyme with sn-1-specific activity, hydrolyzing cardiolipin, PE, PC, PG and PA; implicated in remodeling of mitochondrial phospholipids; antagonistically regulated by Aft1p and Aft2p; in humans, mutations in DDHD1 and DDHD2 genes cause specific types of hereditary spastic paraplegia, while DDL1-defective yeast share similar phenotypes such as mitochondrial dysfunction and defects in lipid metabolism
BUL2	2.26	Alpha-arrestin, component of the Rsp5p E3-ubiquitin ligase complex; ubiquitin-binding adaptor involved in intracellular amino acid permease sorting, functions in heat shock element mediated gene expression, essential for growth in stress conditions; BUL2 has a paralog, BUL1, that arose from the whole genome duplication
CDH1	2.25	Activator of anaphase-promoting complex/cyclosome (APC/C); antagonist of the spindle assembly checkpoint; directs ubiquitination of cyclins resulting in mitotic exit; targets the APC/C to specific substrates including: Cdc20p, Ase1p, Cin8p, Fin1p and Clb5p; partially active in metaphase, and fully active in anaphase; cell-cycle regulated
DNL4	2.23	DNA ligase required for nonhomologous end-joining (NHEJ); forms stable heterodimer with required cofactor Lif1p, interacts with Nej1p; involved in meiosis, not essential for vegetative growth; mutations in human ortholog lead to ligase IV syndrome and Dubowitz syndrome
SEC17	2.23	Alpha-SNAP cochaperone; SNARE-complex adaptor for Sec18 (NSF) during the disassembly of postfusion cis-SNARE complexes; stimulates the ATPase activity of Sec18p; peripheral membrane protein required for vesicular transport between ER and Golgi, the 'priming' step in homotypic vacuole fusion, and autophagy; similar to mammalian alpha-SNAP
DUS4	2.22	Dihydrouridine synthase; member of a widespread family of conserved proteins including Smm1p, Dus1p, and Dus3p
JHD2	2.22	JmjC domain family histone demethylase; promotes global demethylation of H3K4 and represses noncoding intergenic transcription during sporulation; removes methyl groups added by Set1p; phosphorylation by Tpk2p during glycolysis inhibits activity, nuclear localization, chromatin binding and promotes Not4p Ub-mediated degradation; negatively regulated by H3K14 acetylation; regulates sporulation timing by extending period of active transcription; regulates rDNA silencing; human homolog is JARID1C
SAH1	2.21	S-adenosyl-L-homocysteine hydrolase; catabolizes S-adenosyl-L-homocysteine which is formed after donation of the activated methyl group of S-adenosyl-L-methionine (AdoMet) to an acceptor; regulates cellular lipid homoeostasis by regulating phosphatidylcholine(PC)synthesis and triacylglycerol (TG) levels
RCM1	2.21	rRNA m5C methyltransferase; methylates cytosine at position 2278 of 25S rRNA while Nop2p methylates cytosine at position 2870; contains seven beta-strand methyltransferase motif; localized to the nucleolus; interacts with Trm112p; homolog of NSUN5A, a human gene which is deleted in Williams-Beuren Syndrome
MTR2	2.19	mRNA transport regulator; essential nuclear protein; Mex67p and Mtr2p form a mRNA export complex which binds to RNA
YDL211C	2.18	Protein of unknown function; green fluorescent protein (GFP)-fusion protein localizes to the vacuole; YDL211C has a paralog, TDA7, that arose from the whole genome duplication
YPR1	2.17	NADPH-dependent aldo-keto reductase; reduces multiple substrates including 2-methylbutyraldehyde and D,L-glyceraldehyde, expression is induced by osmotic and oxidative stress; functionally redundant with other aldo-keto reductases; protein abundance increases in response to DNA replication stress; YPR1 has a paralog, GCY1, that arose from the whole genome duplication; human homolog AKR1B1 can complement yeast null mutant
DAD2	2.17	Essential subunit of the Dam1 complex (aka DASH complex); complex couples kinetochores to the force produced by MT depolymerization thereby aiding in chromosome segregation; is transferred to the kinetochore prior to mitosis
Ara24	2.17	No description
SCS3	2.15	Protein required for normal ER membrane biosynthesis in response to ER stress; ortholog of the FIT family of proteins involved in triglyceride droplet biosynthesis; human homolog FIT2/FITM2, a fatty acyl-CoA diphosphatase, complements the yeast null mutant; disputed role in the synthesis of inositol phospholipids from inositol; required for inositol prototrophy
ERV1	2.14	Flavin-linked sulfhydryl oxidase of the mitochondrial IMS; N-terminus is an intrinsically disordered domain that in the cytosol helps target Erv1p to mitochondria, and in the intermembrane space oxidizes Mia40p as part of a disulfide relay system that promotes intermembrane space retention of imported proteins; functional ortholog of human GFER (ALR); human GFER carrying N-terminal 21 amino acids of Erv1p functionally complements the lethality of the erv1 null mutation
YCL075W	2.14	Pseudogene: encodes fragment of Ty Pol protein
PGS1	2.12	Phosphatidylglycerolphosphate synthase; catalyzes the synthesis of phosphatidylglycerolphosphate from CDP-diacylglycerol and sn-glycerol 3-phosphate in the first committed and rate-limiting step of cardiolipin biosynthesis
CBP2	2.10	Required for splicing of the group I intron bI5 of the COB pre-mRNA; nuclear-encoded mitochondrial protein that binds to the RNA to promote splicing; also involved in but not essential for splicing of the COB bI2 intron and the intron in the 21S rRNA gene
YPR064W	2.09	Putative protein of unknown function; conserved among S. cerevisiae strains; YPR064W is not an essential gene
YB92	2.08	5'-deoxynucleotidase involved in deoxyribonucleoside monophosphate degradation; green fluorescent protein (GFP)-fusion protein localizes to the cytoplasm and nucleus; non-essential gene; YBR242W has a paralog, YGL101W, that arose from the whole genome duplication
LPX1	2.07	Peroxisomal matrix-localized lipase; required for normal peroxisome morphology; contains a peroxisomal targeting signal type 1 (PTS1) and a lipase motif; peroxisomal import requires the PTS1 receptor, Pex5p and self-interaction; transcriptionally activated by Yrm1p along with genes involved in multidrug resistance; oleic acid inducible
MCM1	2.07	Transcription factor; involved in cell-type-specific transcription and pheromone response; plays a central role in the formation of both repressor and activator complexes; relocalizes to the cytosol in response to hypoxia
MUS81	2.06	Subunit of structure-specific Mms4p-Mus81p endonuclease; cleaves branched DNA; involved in DNA repair, replication fork stability, and joint molecule formation/resolution during meiotic recombination; promotes template switching during break-induced replication (BIR), causing non-reciprocal translocations (NRTs); helix-hairpin-helix protein; phosphorylation of non-catalytic subunit Mms4p by Cdc28p and Cdcp during mitotic cell cycle activates function of Mms4p-Mus81p
ORC6	2.04	Subunit of the origin recognition complex (ORC); ORC directs DNA replication by binding to replication origins and is also involved in transcriptional silencing; phosphorylated by Cdc28p; mutation in the human Orc6p is linked to Meier-Gorlin syndrome
HEL2	2.02	RING finger ubiquitin ligase (E3); subunit of ribosome-associated quality control trigger complex (RQT); involved in ubiquitination and degradation of excess histones; interacts with Ubc4p and Rad53p; required to trigger ribosome-associated quality control (RQC) and canonical no-go decay; ubiquitinates Rps20 and Rps3; binds to ribosomal RNA, mRNA and tRNA
YLL066C	2.02	ORF , Uncharacterized
SMB1	2.02	Core Sm protein Sm B; part of heteroheptameric complex (with Smd1p, Smd2p, Smd3p, Sme1p, Smx3p, and Smx2p) that is part of the spliceosomal U1, U2, U4, and U5 snRNPs; homolog of human Sm B and Sm B
POA1	2.02	Phosphatase that is highly specific for ADP-ribose 1''-phosphate; a tRNA splicing metabolite; may have a role in regulation of tRNA splicing
RPS28A	2.01	Protein component of the small (40S) ribosomal subunit; has an extraribosomal function in regulation of RPS28B, in which Rps28Ap binds to a decapping complex via Edc3p, which then binds to RPS28B mRNA leading to its decapping and degradation; homologous to mammalian ribosomal protein S28, no bacterial homolog; RPS28A has a paralog, RPS28B, that arose from the whole genome duplication
TPK2	2.00	cAMP-dependent protein kinase catalytic subunit; promotes nutrient responsive growth via the Ras-cAMP signaling pathway; phosphorylates and inhibits histone demethylase Jhd2p in response to glucose, promoting H3K4 trimethylation and maintaining H3K14 acetylation by preventing chromatin binding of Rpd3p; required for Sfl1p-mediated pseudohyphal growth; partially redundant with Tpk1p and Tpk3p; localizes to P-bodies during stationary phase; relocalizes to the cytosol in response to hypoxia
PAC10	2.00	Part of the heteromeric co-chaperone GimC/prefoldin complex; complex promotes efficient protein folding
CCP1	2.00	Mitochondrial cytochrome-c peroxidase; degrades reactive oxygen species in mitochondria; acts as hydrogen peroxide sensor in mitochondria, involved in the response to oxidative stress
OCT1	1.99	Mitochondrial intermediate peptidase; cleaves destabilizing N-terminal residues of a subset of proteins upon import, after their cleavage by mitochondrial processing peptidase (Mas1p-Mas2p); may contribute to mitochondrial iron homeostasis
YBL113C	1.97	Putative protein of unknown function; potential Cdc28p substrate
Ara29	1.96	No description
YCR087C-A	1.95	Putative protein of unknown function; green fluorescent protein (GFP)-fusion protein localizes to the nucleolus; YCR087C-A is not an essential gene
Ara15	1.95	No description
YOR012W	1.95	Putative protein of unknown function
SLD3	1.94	Protein involved in the initiation of DNA replication; required for proper assembly of replication proteins at the origins of replication; interacts with the DDK-phosphorylated MCM complex and required for the recruitment of Cdc45p; localizes to nuclear foci that become diffuse upon DNA replication stress; homologous to the human Treslin/Ticrr protein
YAR023C	1.94	Putative integral membrane protein; member of DUP240 gene family
ARF1	1.94	ADP-ribosylation factor; GTPase of the Ras superfamily involved in regulation of coated vesicle formation in intracellular trafficking within the Golgi; involved in mitochondrial morphology; ARF1 has a paralog, ARF2, that arose from the whole genome duplication; arf1 arf2 double null mutation is complemented by human ARF1, ARF4, ARF5, or ARF6
RNR2	1.94	Ribonucleotide-diphosphate reductase (RNR), small subunit; the RNR complex catalyzes the rate-limiting step in dNTP synthesis and is regulated by DNA replication and DNA damage checkpoint pathways via localization of the small subunits; RNR2 has a paralog, RNR4, that arose from the whole genome duplication
YGL239C	1.94	Dubious open reading frame; unlikely to encode a functional protein, based on available experimental and comparative sequence data; partially overlaps verified gene CSE1
AME1	1.93	Essential kinetochore protein associated with microtubules and SPBs; component of the kinetochore sub-complex COMA (Ctf19p, Okp1p, Mcm21p, Ame1p); involved in spindle checkpoint maintenance; orthologous to human centromere constitutive-associated network (CCAN) subunit CENP-U and fission yeast Mis17; relative distribution to the nucleus increases upon DNA replication stress
YNL285W	1.93	Dubious open reading frame; unlikely to encode a functional protein, based on available experimental and comparative sequence data
ATG17	1.93	Scaffold protein responsible for phagophore assembly site organization; regulatory subunit of an autophagy-specific complex that includes Atg1p and Atg13p; stimulates Atg1p kinase activity; human ortholog RB1CC1/FIP200 interacts with p53, which inhibits autophagy in human cells
CLB1	1.93	B-type cyclin involved in cell cycle progression; activates Cdc28p to promote the transition from G2 to M phase; accumulates during G2 and M, then targeted via a destruction box motif for ubiquitin-mediated degradation by the proteasome; CLB1 has a paralog, CLB2, that arose from the whole genome duplication
SML1	1.92	Ribonucleotide reductase inhibitor; involved in regulating dNTP production; regulated by Mec1p and Rad53p during DNA damage and S phase; SML1 has a paralog, DIF1, that arose from the whole genome duplication
MMS21	1.90	Highly conserved SUMO E3 ligase subunit of SMC5-SMC6 complex; required for anchoring dsDNA breaks to the nuclear periphery; SMC5-SMC6 plays a key role in removal of X-shaped DNA structures that arise between sister chromatids during DNA replication and repair; required for efficient sister chromatid cohesion; mutants are sensitive to MMS, show increased spontaneous mutation and mitotic recombination; SUMOylates and inhibits Snf1p function; supports nucleolar function
MAL33	1.90	MAL-activator protein; part of complex locus MAL3; nonfunctional in genomic reference strain S288C
ECM17	1.88	Sulfite reductase beta subunit; involved in amino acid biosynthesis, transcription repressed by methionine
CMR1	1.88	Nuclear protein with role in transcription, protein quality control; localizes to the intranuclear quality control compartment (INQ) in response to proteasome inhibition or DNA replication stress; INQ likely sequesters proteins involved in DNA metabolism for degradation or re-folding; also localizes to coding regions of transcribed genes; contains three WD domains (WD-40 repeat); human ortholog WDR76 also exhibits perinuclear localization under similar stress conditions
END3	1.86	EH domain-containing protein involved in endocytosis; actin cytoskeletal organization and cell wall morphogenesis; forms a complex with Sla1p and Pan1p
MCM6	1.86	Protein involved in DNA replication; component of the Mcm2-7 hexameric helicase complex that binds chromatin as a part of the pre-replicative complex; forms a subcomplex with Mcm4p and Mcm7p
YPD1	1.86	Osmotic stress-responsive phosphorelay intermediate sensor protein; phosphorylated by the plasma membrane sensor Sln1p in response to osmotic stress and then in turn phosphorylates the response regulators Ssk1p in the cytosol and Skn7p in the nucleus
HPM1	1.83	AdoMet-dependent methyltransferase; involved in a novel 3-methylhistidine modification of multiple proteins, including ribosomal protein Rpl3p; seven beta-strand MTase family member; null mutant exhibits a weak vacuolar protein sorting defect and caspofungin resistance

Ara19	1.83	No description
VMA6	1.82	Subunit d of the V0 integral membrane domain of V-ATPase; part of the electrogenic proton pump found in the endomembrane system; required for V1 domain assembly on the vacuolar membrane; the V0 integral membrane domain of vacuolar H+-ATPase (V-ATPase) has five subunits
HXT9	1.81	Putative hexose transporter that is nearly identical to Hxt11p; has similarity to major facilitator superfamily (MFS) transporters, expression of HXT9 is regulated by transcription factors Pdr1p and Pdr3p
TAF12	1.81	Subunit (61/68 kDa) of TFIID and SAGA complexes; involved in RNA polymerase II transcription initiation and in chromatin modification, similar to histone H2A; overexpression of the human ortholog, TAF12, an oncogene involved in the formation of choroid plexus carcinomas, results in dosage chromosomal instability (dCIN) in a human cell line similar to the dCIN observed in yeast overexpressors
GBP2	1.81	Poly(A+) RNA-binding protein; key surveillance factor for the selective export of spliced mRNAs from the nucleus to the cytoplasm; preference for intron-containing genes; similar to Npl3p; also binds single-stranded telomeric repeat sequence in vitro; relocalizes to the cytosol in response to hypoxia; GBP2 has a paralog, HRB1, that arose from the whole genome duplication
YKR040C	1.81	Dubious open reading frame; unlikely to encode a functional protein, based on available experimental and comparative sequence data; partially overlaps the uncharacterized ORF YKR041W
YLR444C	1.80	Dubious open reading frame; unlikely to encode a functional protein, based on available experimental and comparative sequence data
FIG2	1.80	Cell wall adhesin, expressed specifically during mating; may be involved in maintenance of cell wall integrity during mating; FIG2 has a paralog, AGA1, that arose from the whole genome duplication
CDC27	1.80	Subunit of the Anaphase-Promoting Complex/Cyclosome (APC/C); APC/C is a ubiquitin-protein ligase required for degradation of anaphase inhibitors, including mitotic cyclins, during the metaphase/anaphase transition
CDC28	1.80	Cyclin-dependent kinase (CDK) catalytic subunit; master regulator of mitotic and meiotic cell cycles; alternately associates with G1, S, G2/M phase cyclins, which provide substrate specificity; regulates metabolism, basal transcription, chromosome dynamics, growth and morphogenesis; transcript induction in osmostress involves antisense RNA; human homologs CDK1, CDK2, CDK3 can complement yeast conditional cdc28 mutants; human CDK1, CDK2 can complement yeast cdc28 null mutant
NKP1	1.80	Central kinetochore protein and subunit of the Ctf19 complex; mutants have elevated rates of chromosome loss; orthologous to fission yeast kinetochore protein fta4
RIM8	1.79	Protein involved in proteolytic activation of Rim101p; part of response to alkaline pH; interacts with ESCRT-1 subunits Stp22p and Vps28p; essential for anaerobic growth; member of the arrestin-related trafficking adaptor family
ENV9	1.78	Conserved oxidoreductase involved in lipid droplet morphology; mutant shows defects in CPY processing and vacuolar morphology; required for replication of Brome mosaic virus in S. cerevisiae, a model system for studying replication of positive-strand RNA viruses in their natural hosts; homologus to human RDH12 linked to Leber Congenital Amaurosis
TPI1	1.78	Triose phosphate isomerase, abundant glycolytic enzyme; mRNA half-life is regulated by iron availability; transcription is controlled by activators Reb1p, Gcr1p, and Rap1p through binding sites in the 5' non-coding region; inhibition of Tpi1p activity by PEP (phosphoenolpyruvate) stimulates redox metabolism in respiring cells; E104D mutation in human homolog TPI1 causes a rare autosomal disease; human TPI1 can complement yeast null mutant
CIN1	1.77	Tubulin folding factor D involved in beta-tubulin (Tub2p) folding; isolated as mutant with increased chromosome loss and sensitivity to benomyl
RSC2	1.77	Component of the RSC chromatin remodeling complex; required for expression of mid-late sporulation-specific genes; involved in telomere maintenance; RSC2 has a paralog, RSC1, that arose from the whole genome duplication
EMP65	1.76	Integral membrane protein of the ER; forms an ER-membrane associated protein complex with Slp1p; identified along with SLP1 in a screen for mutants defective in the unfolded protein response (UPR); proposed to function in the folding of integral membrane proteins; interacts genetically with MPS3; the authentic, non-tagged protein is detected in highly purified mitochondria in high-throughput studies
FRE8	1.76	Protein with sequence similarity to iron/copper reductases; involved in iron homeostasis; deletion mutant has iron deficiency/accumulation growth defects; expression increased in the absence of copper-responsive transcription factor Mac1p
RPA190	1.76	RNA polymerase I largest subunit A190
POP7	1.76	Subunit of RNase MRP, nuclear RNase P and telomerase; forms a soluble heterodimer with Pop6p that binds P3 domain of RNase MRP and RNase P RNAs; RNase MRP cleaves pre-rRNA, nuclear RNase P cleaves tRNA precursors to generate mature 5' ends and facilitates turnover of nuclear RNAs, while telomerase replenishes telomeric DNA
Ara22	1.75	No description
MAK3	1.74	Catalytic subunit of the NatC type N-terminal acetyltransferase (NAT); involved in subcellular targeting of select N-terminally acetylated substrates to the Golgi apparatus (Arl3p and Grh1p) and the inner nuclear membrane (Trm1p); required for replication of dsRNA virus; human NatC ortholog, NAA30, functionally complements the null, requiring either auxiliary subunit Mak10p or co-expression of human ortholog, NAA35; NAA60, the human NatF gene, also complements the null allele
TMH11	1.74	Mitochondrial protein of unknown function; GFP-fusion protein is induced in response to the DNA-damaging agent MMS; the authentic, non-tagged protein is detected in highly purified mitochondria in high-throughput studies; protein abundance increases in response to DNA replication stress
MXR2	1.73	Methionine-R-sulfoxide reductase; involved in the response to oxidative stress; protects iron-sulfur clusters from oxidative inactivation along with MXR1; involved in the regulation of lifespan
DIA2	1.72	Origin-binding F-box protein; forms SCF ubiquitin ligase complex with Skp1p and Cdc53p; functions in ubiquitination of silent chromatin structural protein Sir4p; required to target Cdc6p for destruction during G1 phase; required for deactivation of Rad53 checkpoint kinase, completion of DNA replication during recovery from DNA damage, assembly of RSC complex, RSC-mediated transcription regulation, and nucleosome positioning; involved in invasive and pseudohyphal growth
BLI1	1.71	Subunit of the BLOC-1 complex involved in endosomal maturation; interacts with Msb3p; green fluorescent protein (GFP)-fusion protein localizes to the endosome
COX2	1.71	Subunit II of cytochrome c oxidase (Complex IV); Complex IV is the terminal member of the mitochondrial inner membrane electron transport chain; one of three mitochondrially-encoded subunits
CBF1	1.71	Basic helix-loop-helix (bHLH) protein; forms homodimer to bind E-box consensus sequence CACGTG present at MET gene promoters and centromere DNA element I (CDEI); affects nucleosome positioning at this motif; associates with other transcription factors such as Met4p and Isw1p to mediate transcriptional activation or repression; associates with kinetochore proteins, required for chromosome segregation; protein abundance increases in response to DNA replication stress
TRM13	1.71	2'-O-methyltransferase; responsible for modification of tRNA at position 4; C-terminal domain has similarity to Rossmann-fold (RFM) superfamily of RNA methyltransferases
SMC4	1.70	Subunit of the condensin complex; condensin reorganizes chromosomes during both mitosis and meiosis; forms a subcomplex with Smc2p that has ATP-hydrolyzing and DNA-binding activity, but other condensin subunits are required for chromatin binding; required for tRNA gene clustering at the nucleolus; potential Cdc28p substrate
ESC8	1.70	Protein involved in telomeric and mating-type locus silencing; interacts with Sir2p and also interacts with Gal11p, which is a component of the RNA pol II mediator complex; ESC8 has a paralog, IOC3, that arose from the whole genome duplication
APS2	1.70	Small subunit of the clathrin-associated adaptor complex AP-2; AP-2 is involved in protein sorting at the plasma membrane; related to the sigma subunit of the mammalian plasma membrane clathrin-associated protein (AP-2) complex
SAE2	1.70	Endonuclease required for telomere elongation; required for telomeric 5' C-rich strand resection; involved in ds-break repair and processing hairpin DNA structures with the MRX complex; function requires sumoylation and phosphorylation; exists as inactive oligomers that are transiently released into smaller active units by phosphorylation; DNA damage triggers Sae2p removal, so active Sae2p is present only transiently; sequence and functional similarity with human CtIP/RBBP8
YOL166C	1.70	Dubious open reading frame; unlikely to encode a functional protein, based on available experimental and comparative sequence data
MRPL9	1.68	Mitochondrial ribosomal protein of the large subunit
ECM2	1.68	Pre-mRNA splicing factor; facilitates the cooperative formation of U2/U6 helix II in association with stem II in the spliceosome, function may be regulated by Slu7p
PSF2	1.67	Subunit of the GINS complex (Sld5p, Psf1p, Psf2p, Psf3p); complex is localized to DNA replication origins and implicated in assembly of the DNA replication machinery
TDA1	1.67	Protein kinase of unknown cellular role; green fluorescent protein (GFP)-fusion protein localizes to the cytoplasm and nucleus; null mutant is sensitive to expression of the top1-T722A allele; not an essential gene; relocalizes from nucleus to cytoplasm upon DNA replication stress
Ara9	1.66	No description
WWM1	1.66	WW domain containing protein of unknown function; binds to Mca1p, a caspase-related protease that regulates H_2O_2-induced apoptosis; overexpression causes G1 phase growth arrest and clonal death that is suppressed by overexpression of MCA1
YBL100W-A	1.66	Retrotransposon TYA Gag gene co-transcribed with TYB Pol; translated as TYA or TYA-TYB polyprotein; Gag is a nucleocapsid protein that is the structural constituent of virus-like particles (VLPs); similar to retroviral Gag
GAL1	1.65	Galactokinase; phosphorylates alpha-D-galactose to alpha-D-galactose-1-phosphate in the first step of galactose catabolism; expression regulated by Gal4p; human homolog GALK2 complements yeast null mutant; GAL1 has a paralog, GAL3, that arose from the whole genome duplication
HVG1	1.65	Protein of unknown function; HVG1 has a paralog, VRG4, that arose from the whole genome duplication
YPS7	1.65	Putative GPI-anchored aspartic protease; member of the yapsin family of proteases involved in cell wall growth and maintenance; located in the cytoplasm and endoplasmic reticulum
CEX1	1.64	Component of COPI-mediated trafficking and tRNA nuclear export; enables Rna1p to access and activate Gsp1p-GTP bound to the export receptor tRNA complex during aminoacylation-dependent tRNA export; copurifies with tRNA export receptors; interacts with COPI coat proteins and regulates Golgi-to-ER trafficking; membrane fraction associated; mutations in members of the homologous human SCY1-like (SCYL) family of pseudokinases are linked to peripheral neuropathy, cerebellar atrophy, ataxia and ALS
YGL176C	1.64	Protein of unknown function; contributes to high inhibitor stress tolerance; deletion mutant is viable
TSR3	1.64	Protein required for 20S pre-rRNA processing; involved in processing of the 20S pre-rRNA at site D to generate mature 18S rRNA; green fluorescent protein (GFP)-fusion protein localizes to both the cytoplasm and the nucleus; relative distribution to the nucleus increases upon DNA replication stress; also detected in peroxisomes
YGL138C	1.63	Putative protein of unknown function; has no significant sequence similarity to any known protein
RTS3	1.63	Putative component of the protein phosphatase type 2A complex
POC4	1.63	Component of a heterodimeric Poc4p-Irc25p chaperone; involved in assembly of alpha subunits into the 20S proteasome; may regulate formation of proteasome isoforms with alternative subunits under different conditions; upregulates proteasome assembly in response to the unfolded protein response activated by mistargeting of proteins (UPRam)
BIO2	1.63	Biotin synthase; catalyzes the conversion of dethiobiotin to biotin, which is the last step of the biotin biosynthesis pathway; complements E. coli bioB mutant
YPL113C	1.63	Glyoxylate reductase; acts on glyoxylate and hydroxypyruvate substrates; YPL113C is not an essential gene
PAM16	1.63	Subunit of the import motor (PAM complex); the PAM complex is a component of the Translocase of the Inner Mitochondrial membrane (TIM23 complex); forms a 1:1 subcomplex with Pam18p and inhibits its cochaperone activity; contains a J-like domain
VPS45	1.61	Protein of the Sec1p/Munc-18 family; essential for vacuolar protein sorting; required for the function of Pep12p and the early endosome/late Golgi SNARE Tlg2p; essential for fusion of Golgi-derived vesicles with the prevacuolar compartment; mutation in human VPS45 is associated with congenital neutropenia and primary myelofibrosis of infancy
VPS4	1.61	AAA-ATPase involved in multivesicular body (MVB) protein sorting; ATP-bound Vps4p localizes to endosomes and catalyzes ESCRT-III disassembly and membrane release; ATPase activity is activated by Vta1p; regulates cellular sterol metabolism
FPR3	1.61	Nucleolar peptidyl-prolyl cis-trans isomerase (PPIase); FK506 binding protein; affects expression of multiple genes via its role in nucleosome assembly; phosphorylated by casein kinase II (Cka1p-Cka2p-Ckb1p-Ckb2p) and dephosphorylated by Ptp1p; PPIase domain acts as a transcriptional repressor when tethered to DNA by lexA, and repressor activity is dependent on PPIase activity; FPR3 has a paralog, FPR4, that arose from the whole genome duplication
JJJ2	1.60	Protein of unknown function; contains a J-domain, which is a region with homology to the E. coli DnaJ protein
CAK1	1.60	Cyclin-dependent kinase-activating kinase; required for passage through the cell cycle; phosphorylates and activates Cdc28p; nucleotide-binding pocket differs significantly from those of most other protein kinases; required for premeiotic DNA synthesis, expression of early and middle sporulation specific genes and direct phosphorylation of the Smk1p MAPK to regulate spore morphogenesis
DFP4	1.60	Putative integral membrane protein; member of DUP240 gene family; green fluorescent protein (GFP)-fusion protein localizes to the plasma membrane in a punctate pattern
IRC11	1.60	Dubious open reading frame; unlikely to encode a functional protein, based on available experimental and comparative sequence data; partially overlaps the uncharacterized gene YOR012W; null mutant displays increased levels of spontaneous Rad52 foci
SPT8	1.60	Subunit of the SAGA transcriptional regulatory complex; not present in SAGA-like complex SLIK/SALSA; required for SAGA-mediated inhibition at some promoters
PSY3	1.59	Component of Shu complex (aka PCSS complex); Shu complex also includes Shu1, Csm2, Shu2, and promotes error-free DNA repair; promotes Rad51p filament assembly; Shu complex mediates inhibition of Srs2p function; Psy3p and Csm2p contain similar DNA-binding regions which work together to form a single DNA binding site; deletion of PSY3 results in a mutator phenotype; deletion increases sensitivity to anticancer drugs oxaliplatin and cisplatin but not mitomycin C
SYF1	1.59	Member of the NineTeen Complex (NTC); that contains Prp19p and stabilizes U6 snRNA in catalytic forms of the spliceosome containing U2, U5, and U6 snRNAs; null mutant has splicing defect and arrests in G2/M; relocalizes to the cytosol in response to hypoxia; homologs in human and C. elegans
MED8	1.59	Subunit of the RNA polymerase II mediator complex; associates with core polymerase subunits to form the RNA polymerase II holoenzyme; essential for transcriptional regulation
LAF1	1.59	Sterol-binding beta-propeller protein; physically associates with sterol-binding StARkin domain-containing proteins Ysp2/Lam2/Ltc4 and Lam4/Ltc3; essential for retrograde transport of ergosterol from the plasma membrane to the endoplasmic reticulum at ER-PM contact sites; transcription is activated by paralogous transcription factors Yrm1p and Yrr1p along with genes involved in multidrug resistance; non-essential gene; has a paralog, DGR2, that arose from the whole genome duplication
RSM7	1.59	Mitochondrial ribosomal protein of the small subunit; has similarity to E. coli S7 ribosomal protein
YLL044W	1.58	Dubious open reading frame; unlikely to encode a functional protein, based on available experimental and comparative sequence data; transcription of both YLL044W and the overlapping gene RPL8B is reduced in the gcr1 null mutant
MIP1	1.58	Mitochondrial DNA polymerase gamma; single subunit of mitochondrial DNA polymerase in yeast, in contrast to metazoan complex of catalytic and accessory subunits; polymorphic in yeast, petites occur more frequently in some lab strains; human ortholog POLG complements yeast mip1 mutant; mutations in human POLG associated with Alpers-Huttenlocher syndrome (AHS), progressive external ophthalmoplegia (PEO), parkinsonism, other mitochondrial diseases
SNT309	1.58	Member of the NineTeen Complex (NTC); this complex contains Prp19p and stabilizes U6 snRNA in catalytic forms of the spliceosome containing U2, U5, and U6 snRNAs; interacts physically and genetically with Prp19p
LCL3	1.57	Putative protein of unknown function; mutant has long chronological lifespan; has homology to Staphylococcus aureus nuclease; GFP-fusion protein localizes to mitochondria; is induced in response to the DNA-damaging agent MMS
IKI3	1.56	Subunit of Elongator complex; Elongator is required for modification of wobble nucleosides in tRNA; maintains structural integrity of Elongator; homolog of human IKAP, mutations in which cause familial dysautonomia (FD)
ADH5	1.56	Alcohol dehydrogenase isoenzyme V; involved in ethanol production; ADH5 has a paralog, ADH1, that arose from the whole genome duplication
YJL135W	1.56	Dubious open reading frame; unlikely to encode a functional protein, based on available experimental and comparative sequence data; partially overlaps the verified genes YJL134W/LCB3
RAD18	1.55	E3 ubiquitin ligase; forms heterodimer with Rad6p to monoubiquitinate PCNA-K164; heterodimer binds single-stranded DNA and has single-stranded DNA dependent ATPase activity; required for postreplication repair; SUMO-targeted ubiquitin ligase (STUbl) that contains a SUMO-interacting motif (SIM) which stimulates its ubiquitin ligase activity towards the sumoylated form of PCNA
PMT4	1.54	Protein O-mannosyltransferase; transfers mannose residues from dolichyl phosphate-D-mannose to protein serine/threonine residues; appears to form homodimers in vivo and does not complex with other Pmt proteins; target for new antifungals
FKH1	1.54	Forkhead family transcription factor; rate-limiting replication origin activator; evolutionarily conserved lifespan regulator; binds multiple chromosomal elements with distinct specificities, cell cycle dynamics; regulates transcription elongation, chromatin silencing at mating loci, expression of G2/M phase genes; facilitates clustering, activation of early-firing replication origins; binds HML recombination enhancer, regulates donor preference during mating-type switching
GMC1	1.54	Protein involved in meiotic progression; mutants are delayed in meiotic nuclear division and are defective in synaptonemal complex assembly; possible membrane-localized protein; SWAT-GFP and mCherry fusion proteins localize to the endoplasmic reticulum and vacuole respectively
ARP2	1.53	Essential component of the Arp2/3 complex; Arp2/3 is a highly conserved actin nucleation center required for the motility and integrity of actin patches; involved in endocytosis and membrane growth and polarity; required for efficient Golgi-to-ER trafficking in COPI mutants
AIM25	1.53	Mitochondria protein of unknown function; interacts genetically with TOR1 to regulate chronological lifespan, and the response to both heat shock and oxidative stress; involved in maintaining the integrity of the mitochondrial network; negative regulator of mitophagy flux; non-tagged protein is detected in purified mitochondria in high-throughput studies; null mutant is viable and displays an elevated frequency of mitochondrial genome loss; similar to murine NOR1
YNL033W	1.53	Putative protein of unknown function; YNL033W has a paralog, YNL019C, that arose from a segmental duplication
Ara20	1.52	No description
GEP3	1.52	Protein required for mitochondrial ribosome small subunit biogenesis; null mutant is defective in respiration and in maturation of 15S rRNA; protein is localized to the mitochondrial inner membrane; null mutant interacts synthetically with prohibitin (Phb1p)
EBP2	1.52	Protein required for 25S rRNA maturation and 60S ribosomal subunit assembly; localizes to the nucleolus and in foci along nuclear periphery; constituent of 66S pre-ribosomal particles; cooperates with Rrs1p and Mps3p to mediate telomere clustering by binding Sir4p, but is not involved in telomere tethering
SWI3	1.51	Subunit of the SWI/SNF chromatin remodeling complex; SWI/SNF regulates transcription by remodeling chromosomes; contains SANT domain that is required for SWI/SNF assembly; is essential for displacement of histone H2A-H2B dimers during ATP-dependent remodeling; required for transcription of many genes, including ADH1, ADH2, GAL1, HO, INO1 and SUC2; relocates to the cytosol under hypoxic conditions
ULI1	1.51	Protein of unknown function; involved in and induced by the endoplasmic reticulum unfolded protein response (UPR); SWAT-GFP and mCherry fusion proteins localize to the endoplasmic reticulum
LSC2	-1.50	Beta subunit of succinyl-CoA ligase; succinyl-CoA ligase is a mitochondrial enzyme of the TCA cycle that catalyzes the nucleotide-dependent conversion of succinyl-CoA to succinate
UCC1	-1.51	F-box protein and component of SCF ubiquitin ligase complexes; involved in ubiquitin-dependent protein catabolism; readily monoubiquitinated in vitro by SCF-Ubc4 complexes; SCF-Ucc1 regulates level of Cit2 citrate synthase protein to maintain citrate homeostasis, acts as metabolic switch for glyoxylate cycle; UCC1 transcription is downregulated in cells grown on C2-compounds
ERG27	-1.51	3-keto sterol reductase; catalyzes last of three steps required to remove two C-4 methyl groups from an intermediate in ergosterol biosynthesis; Erg27 protein is mainly ER-localized in strains devoid of mtDNA (rho° cells); mutants are sterol auxotrophs; mutation is functionally complemented by human HSD17B7
YOL019W	-1.51	Protein involved in secretion and cell wall organization; localizes to plasma membrane microdomains called MCCs (membrane compartment occupied by Can1); GFP-fusion protein localizes to the cell periphery and vacuole; Sur7/PalI family transmembrane domain (TMD) containing protein; TOS7 has a paralog, DCV1, that arose from the whole genome duplication
YBL100W-B	-1.52	Retrotransposon TYA Gag and TYB Pol genes; transcribed/translated as one unit; polyprotein is processed to make a nucleocapsid-like protein (Gag), reverse transcriptase (RT), protease (PR), and integrase (IN); similar to retroviral genes
YBL100W-A	-1.52	Retrotransposon TYA Gag gene co-transcribed with TYB Pol; translated as TYA or TYA-TYB polyprotein; Gag is a nucleocapsid protein that is the structural constituent of virus-like particles (VLPs); similar to retroviral Gag
YBL100W-B	-1.52	Retrotransposon TYA Gag and TYB Pol genes; transcribed/translated as one unit; polyprotein is processed to make a nucleocapsid-like protein (Gag), reverse transcriptase (RT), protease (PR), and integrase (IN); similar to retroviral genes
SAC3	-1.53	mRNA export factor; required for biogenesis of the small ribosomal subunit; component of TREX-2 complex (Sac3p-Thp1p-Sus1p-Cdc31p) involved in transcription elongation and mRNA export from the nucleus; involved in post-transcriptional tethering of active genes to the nuclear periphery and to non-nascent mRNP; similar to the human germinal center-associated nuclear protein (GANP)
YJL007C	-1.53	Putative protein of unknown function; conserved among S. cerevisiae strains
RPP2A	-1.53	Ribosomal protein P2 alpha; a component of the ribosomal stalk, which is involved in the interaction between translational elongation factors and the ribosome; free (non-ribosomal) P2 stimulates the phosphorylation of the eIF2 alpha subunit (Sui2p) by Gcn2p; regulates the accumulation of P1 (Rpp1Ap and Rpp1Bp) in the cytoplasm
YPR089W	-1.53	Protein of unknown function; exhibits genetic interaction with ERG11 and protein-protein interaction with Hsp82p
EFR3	-1.54	Protein required for Stt4-containing PI kinase complex localization; required for Stt4-containing phosphoinositide (PI) kinase patch assembly at plasma membrane; recruited to plasma membrane via conserved basic patch near N-terminus; exhibits synthetic lethal genetic interactions with PHO85; mutations in human homolog EFR3A implicated in autism spectrum disorder
YBL100W-A	-1.54	Retrotransposon TYA Gag gene co-transcribed with TYB Pol; translated as TYA or TYA-TYB polyprotein; Gag is a nucleocapsid protein that is the structural constituent of virus-like particles (VLPs); similar to retroviral Gag
YER119C-A	-1.54	Dubious open reading frame; unlikely to encode a functional protein, based on available experimental and comparative sequence data; not conserved in closely related Saccharomyces species; deletion mutation blocks replication of Brome mosaic virus in S. cerevisiae, but this is likely due to effects on the overlapping gene SCS2
PAU1	-1.54	Protein of unknown function; member of the seripauperin multigene family encoded mainly in subtelomeric regions; identical to Pau6p
SKG1	-1.55	Transmembrane protein with a role in cell wall polymer composition; localizes on inner surface of plasma membrane at bud and in daughter cell; SKG1 has a paralog, AIM20, that arose from the whole genome duplication

YGL101W	-1.55	Putative protein of unknown function; epitope-tagged protein localizes to the cytoplasm
YBR226C	-1.55	Dubious open reading frame; unlikely to encode a functional protein, based on available experimental and comparative sequence data; partially overlaps the uncharacterized ORF YBR225W
YAR009C	-1.55	Retrotransposon TYA Gag and TYB Pol genes; transcribed/translated as one unit; polyprotein is processed to make a nucleocapsid-like protein (Gag), reverse transcriptase (RT), protease (PR), and integrase (IN); similar to retroviral genes
YCL042W	-1.56	Putative protein of unknown function; epitope-tagged protein localizes to the cytoplasm
YMR316C-B	-1.56	Dubious open reading frame; unlikely to encode a functional protein, based on available experimental and comparative sequence data; almost completely overlaps 5' end of ORF YMR317W
FZO1	-1.56	Mitofusin, protein involved in mitochondrial outer membrane fusion; role in mitochondrial genome maintenance; efficient tethering and degradation of Fzo1p requires an intact N-terminal GTPase domain; targeted for destruction by the ubiquitin ligase SCF-Mdm30p and the cytosolic ubiquitin-proteasome system; activity regulated by ubiquitylation at conserved lysine residues and by deubiquitylases Ubp2p and Ubp12p
DBP8	-1.56	ATPase, putative RNA helicase of the DEAD-box family; component of 90S preribosome complex involved in production of 18S rRNA and assembly of 40S small ribosomal subunit; ATPase activity stimulated by association with Esf2p
TAF10	-1.56	Subunit (145 kDa) of TFIID and SAGA complexes; involved in RNA polymerase II transcription initiation and in chromatin modification
YJL086C	-1.56	Dubious open reading frame; unlikely to encode a functional protein, based on available experimental and comparative sequence data; partially overlaps the verified genes YJL085W/EXO70 and YJL087C/TRL1
FBA1	-1.57	Fructose 1,6-bisphosphate aldolase; required for glycolysis and gluconeogenesis; catalyzes conversion of fructose 1,6 bisphosphate to glyceraldehyde-3-P and dihydroxyacetone-P; locates to mitochondrial outer surface upon oxidative stress; N-terminally propionylated in vivo
YJL211C	-1.57	Dubious open reading frame; unlikely to encode a functional protein, based on available experimental and comparative sequence data; partially overlaps the verified gene YJL210W/PEX2
YAR010C	-1.57	Retrotransposon TYA Gag gene co-transcribed with TYB Pol; translated as TYA or TYA-TYB polyprotein; Gag is a nucleocapsid protein that is the structural constituent of virus-like particles (VLPs); similar to retroviral Gag
LDB16	-1.57	Protein involved in lipid droplet (LD) assembly; forms a complex with Sei1p at ER-LD contact sites, stabilizing contact sites; ensures that LDs bud from the ER towards the cytosolic side of the membrane; null mutants have decreased net negative cell surface charge and localized accumulation of phosphatidic acid (PA) marker proteins; GFP-fusion protein expression is induced in response to MMS; null mutant can be complemented by the human seipin, BSCL2
SEO1	-1.58	Putative permease; member of the allantoate transporter subfamily of the major facilitator superfamily; mutation confers resistance to ethionine sulfoxide
HXT2	-1.58	High-affinity glucose transporter of the major facilitator superfamily; expression is induced by low levels of glucose and repressed by high levels of glucose
YLR296W	-1.58	Putative protein of unknown function; conserved across S. cerevisiae strains
APM1	-1.59	Mu1-like medium subunit of the AP-1 complex; binds clathrin; involved in clathrin-dependent Golgi protein sorting; the AP-1 complex is the clathrin-associated protein complex
ANP1	-1.59	Subunit of the alpha-1,6 mannosyltransferase complex; type II membrane protein; has a role in retention of glycosyltransferases in the Golgi; involved in osmotic sensitivity and resistance to aminonitrophenyl propanediol
DIF1	-1.59	Protein that regulates nuclear import of Rnr2p and Rnr4p; phosphorylated by Dun1p in response to DNA damage and degraded; N-terminal half shows similarity to S. pombe Spd1 protein; DIF1 has a paralog, SML1, that arose from the whole genome duplication
YPL071C	-1.59	Putative protein of unknown function; green fluorescent protein (GFP)-fusion protein localizes to both the cytoplasm and the nucleus
MUK1	-1.60	Guanine nucleotide exchange factor (GEF); involved in vesicle-mediated vacuolar transport, including Golgi-endosome trafficking and sorting through the multivesicular body (MVB); specifically stimulates the intrinsic guanine nucleotide exchange activity of Rab family members (Vps21p/Ypt52p/Ypt53p); partially redundant with GEF VPS9; required for localization of the CORVET complex to endosomes; contains a VPS9 domain
YAR010C	-1.60	Retrotransposon TYA Gag gene co-transcribed with TYB Pol; translated as TYA or TYA-TYB polyprotein; Gag is a nucleocapsid protein that is the structural constituent of virus-like particles (VLPs); similar to retroviral Gag
YOR238W	-1.61	Putative protein of unknown function; green fluorescent protein (GFP)-fusion protein localizes to the cytoplasm
DMA1, CHF1	-1.61	Ubiquitin-protein ligase (E3); controls septin dynamics, spindle position checkpoint (SPOC) with ligase Dma2p by regulating recruitment of Elm1p to bud neck; regulates levels of eIF2 subunit Gcd11p, as well as abundance, localization, and ubiquitination of Cdk inhibitory kinase Swe1p; ubiquitinates cyclin Pcl1p; ortholog of human RNF8, similar to human Chfr; contains FHA, RING fingers; DMA1 has a paralog, DMA2, that arose from the whole genome duplication
YMR114C	-1.61	Protein of unknown function; may interact with ribosomes, based on co-purification experiments; green fluorescent protein (GFP)-fusion protein localizes to the nucleus and cytoplasm; YMR114C is not an essential gene
TMA64	-1.62	Protein of unknown function that associates with ribosomes; has a putative RNA binding domain; in mammals the corresponding protein, eIF2D, has been shown to possess translation initiation factor activity
RFC3	-1.62	Subunit of heteropentameric Replication factor C (RF-C); which is a DNA binding protein and ATPase that acts as a clamp loader of the proliferating cell nuclear antigen (PCNA) processivity factor for DNA polymerases delta and epsilon; relocalizes to the cytosol in response to hypoxia
DAN2	-1.62	Protein of unknown function; member of the seripauperin multigene family encoded mainly in subtelomeric regions; SWAT-GFP and mCherry fusion proteins localize to the vacuole; YLL025W is not an essential gene 2
IFH1	-1.62	Coactivator, regulates transcription of ribosomal protein (RP) genes; recruited to RP gene promoters during optimal growth conditions via Fhl1p; subunit of CURI, a complex that coordinates RP production and pre-rRNA processing; regulated by acetylation and phosphorylation at different growth states via TORC1 signaling; IFH1 has a paralog, CRF1, that arose from the whole genome duplication
EGD1	-1.62	Subunit beta1 of the nascent polypeptide-associated complex (NAC); involved in protein targeting, associated with cytoplasmic ribosomes; enhances DNA binding of the Gal4p activator; homolog of human BTF3b; EGD1 has a paralog, BTT1, that arose from the whole genome duplication
GID8	-1.63	Subunit of GID Complex, binds strongly to central component Vid30p; GID Complex is involved in proteasome-dependent catabolite inactivation of fructose-1,6-bisphosphatase; recruits Rmd5p, Fyv10 and Vid28p to GID Complex; contains LisH, CTLH, and CRA domains that mediate binding to Vid30p (LisH) and Rmd5p and Vid28p (CTLH and CRA); dosage-dependent regulator of START
YAR009C	-1.63	Retrotransposon TYA Gag and TYB Pol genes; transcribed/translated as one unit; polyprotein is processed to make a nucleocapsid-like protein (Gag), reverse transcriptase (RT), protease (PR), and integrase (IN); similar to retroviral genes
YBL005W-A	-1.63	Retrotransposon TYA Gag gene co-transcribed with TYB Pol; translated as TYA or TYA-TYB polyprotein; Gag is a nucleocapsid protein that is the structural constituent of virus-like particles (VLPs); similar to retroviral Gag
YJR115W	-1.64	Putative protein of unknown function; YJR115W has a paralog, ECM13, that arose from the whole genome duplication
RPL38	-1.64	Ribosomal 60S subunit protein L38; homologous to mammalian ribosomal protein L38, no bacterial homolog
HTC1	-1.64	Putative protein of unknown function; green fluorescent protein (GFP)-fusion protein localizes to the cytoplasm; YPL067C is not an essential gene
SWI1	-1.64	Subunit of the SWI/SNF chromatin remodeling complex; regulates transcription by remodeling chromatin; required for transcription of many genes, including ADH1, ADH2, GAL1, HO, INO1 and SUC2; self-assembles to form [SWI+] prion and to alter expression pattern; human homolog ARID1A is a candidate tumor suppressor gene in breast cancer
YBL005W-A	-1.64	Retrotransposon TYA Gag gene; Gag is a nucleocapsid protein that is the structural constituent of virus-like particles (VLPs); similar to retroviral Gag; YDR170W-A is part of a mutant retrotransposon; distribution in the cytoplasm becomes irregular rather than punctate upon DNA replication stress
RPS3	-1.64	Protein component of the small (40S) ribosomal subunit; has apurinic/apyrimidinic (AP) endonuclease activity; essential for viability; nascent Rps3p is bound by specific chaperone Yar1p during translation; homologous to mammalian ribosomal protein S3 and bacterial S3
YBL100W-B	-1.65	Retrotransposon TYA Gag and TYB Pol genes; transcribed/translated as one unit; polyprotein is processed to make a nucleocapsid-like protein (Gag), reverse transcriptase (RT), protease (PR), and integrase (IN); similar to retroviral genes
RPT2	-1.65	ATPase of the 19S regulatory particle of the 26S proteasome; one of six ATPases of the regulatory particle; involved in the degradation of ubiquitinated substrates; required for normal peptide hydrolysis by the core 20S particle; N-myristoylation of Rpt2p at Gly2 is involved in regulating the proper intracellular distribution of proteasome activity by controlling the nuclear localization of the 26S proteasome
YAR010C	-1.65	Retrotransposon TYA Gag gene co-transcribed with TYB Pol; Gag processing produces capsid proteins; in YDRCTy1-3 TYB is mutant and probably non-functional
SMD2	-1.66	Core Sm protein Sm D2; part of heteroheptameric complex (with Smb1p, Smd1p, Smd3p, Sme1p, Smx3p, and Smx2p) that is part of the spliceosomal U1, U2, U4, and U5 snRNPs; homolog of human Sm D2
ANR2	-1.66	Protein of unknown function; may have a role in lipid metabolism, based on localization to lipid droplets; predicted to be palmitoylated
OM45	-1.66	Mitochondrial outer membrane protein of unknown function; major constituent of the outer membrane, extending into the intermembrane space; interacts with porin (Por1p) and with Om14p; imported via the presequence pathway involving the TOM and TIM23 complexes, then assembled in the outer membrane by Mim1p; protein abundance increases in response to DNA replication stress
YCR023C	-1.67	Vacuolar membrane protein of unknown function; targeted to vacuole via AP-3 pathway; member of multidrug resistance family; not an essential gene
SNU71	-1.67	Component of U1 snRNP required for mRNA splicing via spliceosome; yeast specific, no metazoan counterpart
COQ11	-1.67	Putative oxidoreductase, subunit of Coenzyme Q biosynthetic complexes; required for synthesis of wild-type levels of Coenzyme Q (ubiquinone); member of the short-chain dehydrogenase/reductase (SDR) superfamily; orthologous gene in some other fungi is fused to the COQ10 ortholog
YJR115W	-1.68	Putative protein of unknown function; YJR115W has a paralog, ECM13, that arose from the whole genome duplication
YDL034W	-1.68	Dubious open reading frame; unlikely to encode a functional protein, based on available experimental and comparative sequence data; partially overlaps with verified gene GPR1/YDL035C; YDL034W is not an essential gene
YDL012C	-1.68	Tail-anchored plasma membrane protein with a conserved CYSTM module; possibly involved in response to stress; may contribute to non-homologous end-joining (NHEJ) based on ydl012c htz1 double null phenotype; YDL012C has a paralog, YBR016W, that arose from the whole genome duplication
HAT2	-1.68	Subunit of the Hat1p-Hat2p histone acetyltransferase complex; required for high affinity binding of the complex to free histone H4, thereby enhancing Hat1p activity; similar to human RbAp46 and 48; has a role in telomeric silencing
YOL050C	-1.68	Dubious open reading frame; unlikely to encode a functional protein, based on available experimental and comparative sequence data; overlaps verified gene GAL11; deletion confers sensitivity to 4-(N-(S-glutathionylacetyl)amino) phenylarsenoxide (GSAO)
LYS21	-1.69	Homocitrate synthase isozyme; catalyzes the condensation of acetyl-CoA and alpha-ketoglutarate to form homocitrate, which is the first step in the lysine biosynthesis pathway; LYS21 has a paralog, LYS20, that arose from the whole genome duplication
YBL005W-A	-1.69	Retrotransposon TYA Gag gene co-transcribed with TYB Pol; translated as TYA or TYA-TYB polyprotein; Gag is a nucleocapsid protein that is the structural constituent of virus-like particles (VLPs); similar to retroviral Gag
YBL044W	-1.69	Putative protein of unknown function; YBL044W is not an essential protein
YBL100W-B	-1.70	TyB Gag-Pol protein; proteolytically processed to make the Gag, reverse transcriptase (RT), protease (PR), and integrase (IN) proteins that are required for retrotransposition
PMT1	-1.70	Protein O-mannosyltransferase of the ER membrane; transfers mannose from dolichyl phosphate-D-mannose to protein serine and threonine residues; 1 of 7 related proteins involved in O-glycosylation which is essential for cell wall rigidity; functions as a heterodimer with Pmt2p but can also pair with Pmt3p; involved in ER quality control; amino terminus faces cytoplasm, carboxyl terminus faces ER lumen
PFK27	-1.71	6-phosphofructo-2-kinase; catalyzes synthesis of fructose-2,6-bisphosphate; inhibited by phosphoenolpyruvate and sn-glycerol 3-phosphate, expression induced by glucose and sucrose, transcriptional regulation involves protein kinase A
CMK2	-1.71	Calmodulin-dependent protein kinase; negative feedback controller of calcium/calcineurin signalling pathway, also has additional Crz1-independent role in promoting calcium tolerance; amino acid sequence similar to mammalian Cam Kinase II; CMK2 has a paralog, CMK1, that arose from the whole genome duplication
PRP3	-1.71	Splicing factor; component of the U4/U6-U5 snRNP complex
BUD28	-1.71	Dubious open reading frame; unlikely to encode a functional protein, based on available experimental and comparative sequence data; not conserved in closely related Saccharomyces species; 98% of ORF overlaps the verified gene RPL22A; diploid mutant displays a weak budding pattern phenotype in a systematic assay
CWC27	-1.71	Component of a complex containing Cef1p; putatively involved in pre-mRNA splicing; has similarity to S. pombe Cwf27p; protein abundance increases in response to DNA replication stress
KAP95	-1.72	Karyopherin beta; forms a complex with Srp1p/Kap60p; interacts with nucleoporins to mediate nuclear import of NLS-containing cargo proteins via the nuclear pore complex; regulates PC biosynthesis; GDP-to-GTP exchange factor for Gsp1p
YPL108W	-1.72	Cytoplasmic protein of unknown function; non-essential gene that is induced in a GDH1 deleted strain with altered redox metabolism; GFP-fusion protein is induced in response to the DNA-damaging agent MMS
SEN34	-1.72	Subunit of the tRNA splicing endonuclease; tRNA splicing endonuclease (Sen complex) is composed of Sen2p, Sen15p, Sen34p, and Sen54p; Sen complex also cleaves the CBP1 mRNA at the mitochondrial surface; Sen34p contains the active site for tRNA 3' splice site cleavage and has similarity to Sen2p and to Archaeal tRNA splicing endonuclease
YAR010C	-1.72	Retrotransposon TYA Gag gene co-transcribed with TYB Pol; translated as TYA or TYA-TYB polyprotein; Gag is a nucleocapsid protein that is the structural constituent of virus-like particles (VLPs); similar to retroviral Gag
RNH70	-1.73	3'-5' exoribonuclease; required for maturation of 3' ends of 5S rRNA and tRNA-Arg3 from dicistronic transcripts
YER158C	-1.74	Protein of unknown function; potentially phosphorylated by Cdc28p; YER158C has a paralog, AFR1, that arose from the whole genome duplication
YLR184W	-1.76	Dubious open reading frame; unlikely to encode a functional protein, based on available experimental and comparative sequence data
MRPL31	-1.76	Mitochondrial ribosomal protein of the large subunit
YCK1	-1.76	Palmitoylated plasma membrane-bound casein kinase I (CK1) isoform; shares redundant functions with Yck2p in morphogenesis, proper septin assembly, endocytic trafficking, and glucose sensing; stabilized by Sod1p binding in the presence of glucose and oxygen, causing glucose repression of respiratory metabolism; involved in the phosphorylation and regulation of glucose sensor Rgt2p; YCK1 has a paralog, YCK2, that arose from the whole genome duplication
DDR2	-1.77	Multi-stress response protein; expression is activated by a variety of xenobiotic agents and environmental or physiological stresses; DDR2 has a paralog, HOR7, that arose from the whole genome duplication
CCW22	-1.77	Cell wall protein; YDR134C has a paralog, CCW12, that arose from the whole genome duplication; S. cerevisiae genome reference strain S288C contains an internal in-frame stop at codon 67, which in other strains encodes glutamine
HLR1	-1.77	Protein involved in regulation of cell wall composition and integrity; also involved in cell wall response to osmotic stress; overproduction suppresses a lysis sensitive PKC mutation; HLR1 has a paralog, LRE1, that arose from the whole genome duplication
YMR194C-A	-1.77	Dubious open reading frame; unlikely to encode a functional protein, based on available experimental and comparative sequence data
MF(ALPHA)2	-1.78	Mating pheromone alpha-factor, made by alpha cells; interacts with mating type a cells to induce cell cycle arrest and other responses leading to mating; also encoded by MF(ALPHA)1, which is more highly expressed; binds copper(II) ions
ENB1	-1.78	Ferric enterobactin transmembrane transporter; expressed under conditions of iron deprivation; member of the major facilitator superfamily; expression is regulated by Rcs1p and affected by chloroquine treatment
UTP15	-1.79	Nucleolar protein; component of the small subunit (SSU) processome containing the U3 snoRNA that is involved in processing of pre-18S rRNA
ATP19	-1.79	Subunit k of the mitochondrial F1F0 ATP synthase; F1F0 ATP synthase is a large, evolutionarily conserved enzyme complex required for ATP synthesis; associated only with the dimeric form of ATP synthase
YAR010C	-1.79	Retrotransposon TYA Gag gene co-transcribed with TYB Pol; translated as TYA or TYA-TYB polyprotein; Gag is a nucleocapsid protein that is the structural constituent of virus-like particles (VLPs); similar to retroviral Gag
EMP24	-1.80	Component of the p24 complex; role in misfolded protein quality control; binds to GPI anchor proteins and mediates their efficient transport from the ER to the Golgi; integral membrane protein that associates with endoplasmic reticulum-derived COPII-coated vesicles
YAR009C	-1.80	Retrotransposon TYA Gag and TYB Pol genes; transcribed/translated as one unit; polyprotein is processed to make a nucleocapsid-like protein (Gag), reverse transcriptase (RT), protease (PR), and integrase (IN); similar to retroviral genes
IRC9	-1.80	Putative protein of unknown function; partially overlaps verified gene YAK1/YJL141C but does not share all phenotypes; null mutant displays increased levels of spontaneous Rad52p foci, increased sporulation efficiency, and small defect in vacuolar fragmentation
MSB1	-1.81	Protein of unknown function; may be involved in positive regulation of 1,3-beta-glucan synthesis and the Pkc1p-MAPK pathway; multicopy suppressor of temperature-sensitive mutations in CDC24 and CDC42, and of mutations in BEM4; potential Cdc28p substrate; relocalizes from bud neck to cytoplasm upon DNA replication stress
MSC6	-1.81	Multicopy suppressor of HER2 involved in mitochondrial translation; mutant is defective in directing meiotic recombination events to homologous chromatids
NGR1	-1.82	RNA binding protein that negatively regulates growth rate; interacts with the 3' UTR of the mitochondrial porin (POR1) mRNA and enhances its degradation; overexpression impairs mitochondrial function; interacts with Dhh1p to mediate POR1 mRNA decay; expressed in stationary phase
SYG1	-1.82	Plasma membrane protein of unknown function; targeted to vacuole via AP-3 pathway; truncation and overexpression suppresses lethality of G-alpha protein deficiency
HXT3	-1.83	Low affinity glucose transporter of the major facilitator superfamily; expression is induced in low or high glucose conditions; HXT3 has a paralog, HXT5, that arose from the whole genome duplication|
YAR010C	-1.83	Retrotransposon TYA Gag gene co-transcribed with TYB Pol; translated as TYA or TYA-TYB polyprotein; Gag is a nucleocapsid protein that is the structural constituent of virus-like particles (VLPs); similar to retroviral Gag
YAR010C	-1.83	Retrotransposon TYA Gag gene co-transcribed with TYB Pol; translated as TYA or TYA-TYB polyprotein; Gag is a nucleocapsid protein that is the structural constituent of virus-like particles (VLPs); similar to retroviral Gag
YBR012W-A	-1.83	Retrotransposon TYA Gag gene co-transcribed with TYB Pol; translated as TYA or TYA-TYB polyprotein; Gag is a nucleocapsid protein that is the structural constituent of virus-like particles (VLPs); similar to retroviral Gag
TOM20	-1.84	Component of the TOM (translocase of outer membrane) complex; responsible for recognition and initial import steps for all mitochondrially directed proteins; acts as a receptor for incoming precursor protein
RPL43A	-1.84	Ribosomal 60S subunit protein L43A; null mutation confers a dominant lethal phenotype; homologous to mammalian ribosomal protein L37A, no bacterial homolog; RPL43A has a paralog, RPL43B, that arose from the whole genome duplication
MUD1	-1.85	U1 snRNP A protein; homolog of human U1-A; involved in nuclear mRNA splicing
NAM8	-1.85	RNA binding protein, component of the U1 snRNP protein; mutants are defective in meiotic recombination and in formation of viable spores, involved in the formation of DSBs through meiosis-specific splicing of REC107 pre-mRNA; Nam8p regulon embraces the meiotic pre-mRNAs of REC107, HFM1, AMA1 SPO22 and PCH2; the putative RNA binding domains RRM2 and RRM3 are required for Nam8p meiotic function
POP2	-1.86	Subunit of Ccr4-Not complex that mediates 3' to 5' mRNA deadenylation; exonuclease of the DEDD superfamily
ATE1	-1.86	Arginyl-tRNA-protein transferase; catalyzes post-translational conjugation of arginine to the amino termini of acceptor proteins which are then subject to degradation via the N-end rule pathway; may have a role in regulating stress response
YAR009C	-1.87	Retrotransposon TYA Gag and TYB Pol genes; polyprotein is processed to make a nucleocapsid-like protein (Gag), reverse transcriptase (RT), protease (PR), and integrase (IN); YLR035C-A is part of a mutant retrotransposon
GLO4	-1.87	Mitochondrial glyoxalase II; catalyzes the hydrolysis of S-D-lactoylglutathione into glutathione and D-lactate; GLO4 has a paralog, GLO2, that arose from the whole genome duplication
MAS6	-1.89	Essential component of the TIM23 complex; involved in protein import into mitochondrial matrix and inner membrane; with Tim17p, contributes to architecture and function of the import channel; TIM23 complex is short for the translocase of the inner mitochondrial membrane
RPL24B	-1.90	Ribosomal 60S subunit protein L24B; forms two bridges within ribosome, stimulates translation initiation and elongation; homologous to mammalian ribosomal protein L24, no bacterial homolog; RPL24B has a paralog, RPL24A, that arose from the whole genome duplication
CDC19	-1.90	Pyruvate kinase; functions as a homotetramer in glycolysis to convert phosphoenolpyruvate to pyruvate, the input for aerobic (TCA cycle) or anaerobic (glucose fermentation) respiration; regulated via allosteric activation by fructose bisphosphate; CDC19 has a paralog, PYK2, that arose from the whole genome duplication
COS1	-1.92	Endosomal protein involved in turnover of plasma membrane proteins; member of the DUP380 subfamily of conserved, often subtelomeric COS genes; required for the multivesicular vesicle body sorting pathway that internalizes plasma membrane proteins for degradation; Cos proteins provide ubiquitin in trans for nonubiquitinated cargo proteins
PSP1	-1.92	Asn and gln rich protein of unknown function; high-copy suppressor of POL1 (DNA polymerase alpha) and partial suppressor of CDC2 (polymerase delta) and CDC6 (pre-RC loading factor) mutations; overexpression results in growth inhibition; capable of forming the prion [PSP1+]; PSP1 has a paralog, YLR177W, that arose from the whole genome duplication
YIL032C	-1.92	Dubious open reading frame; unlikely to encode a functional protein, based on available experimental and comparative sequence data
URB1	-1.93	Protein required for the normal accumulation of 25S and 5.8S rRNAs; nucleolar protein; associated with the 27SA2 pre-ribosomal particle; proposed to be involved in the biogenesis of the 60S ribosomal subunit
YAR010C	-1.93	Retrotransposon TYA Gag gene co-transcribed with TYB Pol; translated as TYA or TYA-TYB polyprotein; Gag is a nucleocapsid protein that is the structural constituent of virus-like particles (VLPs); similar to retroviral Gag
RPS4B	-1.95	Protein component of the small (40S) ribosomal subunit; homologous to mammalian ribosomal protein S4, no bacterial homolog; RPS4B has a paralog, RPS4A, that arose from the whole genome duplicatio

YOL046C	-1.96	Dubious open reading frame; unlikely to encode a functional protein, based on available experimental and comparative sequence data; almost completely overlaps the verified gene PSK2/YOL045W
YAR010C	-1.96	Retrotransposon TYA Gag gene co-transcribed with TYB Pol; translated as TYA or TYA-TYB polyprotein; Gag is a nucleocapsid protein that is the structural constituent of virus-like particles (VLPs); similar to retroviral Gag
YBL100W-B	-1.98	Retrotransposon TYA Gag and TYB Pol genes; transcribed/translated as one unit; polyprotein is processed to make a nucleocapsid-like protein (Gag), reverse transcriptase (RT), protease (PR), and integrase (IN); similar to retroviral genes
HMS2	-1.99	Protein with similarity to heat shock transcription factors; overexpression suppresses the pseudohyphal filamentation defect of a diploid mep1 mep2 homozygous null mutant; HMS2 has a paralog, SKN7, that arose from the whole genome duplication
YDR269C	-1.99	Dubious open reading frame; unlikely to encode a functional protein, based on available experimental and comparative sequence data
SWI5	-2.01	Transcription factor that recruits Mediator and Swi/Snf complexes; activates transcription of genes expressed at the M/G1 phase boundary and in G1 phase; required for expression of the HO gene controlling mating type switching; localization to nucleus occurs during G1 and appears to be regulated by phosphorylation by Cdc28p kinase; SWI5 has a paralog, ACE2, that arose from the whole genome duplication
CUP1-1	-2.01	Metallothionein; binds copper and mediates resistance to high concentrations of copper and cadmium; locus is variably amplified in different strains, with two copies, CUP1-1 and CUP1-2, in the genomic sequence reference strain S288C; CUP1-1 has a paralog, CUP1-2, that arose from a segmental duplication
CTA1	-2.03	Catalase A; breaks down hydrogen peroxide in the peroxisomal matrix formed by acyl-CoA oxidase (Pox1p) during fatty acid beta-oxidation
YDR316W-B	-2.03	Retrotransposon TYA Gag and TYB Pol genes; transcribed/translated as one unit; polyprotein is processed to make a nucleocapsid-like protein (Gag), reverse transcriptase (RT), protease (PR), and integrase (IN); similar to retroviral genes
STE20	-2.03	Cdc42p-activated signal transducing kinase; involved in pheromone response, pseudohyphal/invasive growth, vacuole inheritance, down-regulation of sterol uptake; GBB motif binds Ste4p; member of the PAK (p21-activated kinase) family
Ara5	-2.06	No description
YDL023C	-2.06	Dubious open reading frame; unlikely to encode a functional protein, based on available experimental and comparative sequence data; not conserved in other Saccharomyces species; overlaps the verified gene GPD1; deletion confers sensitivity to GSAO; deletion in cyr1 mutant results in loss of stress resistance
LEM3	-2.07	Membrane protein of the plasma membrane and ER; interacts specifically in vivo with the phospholipid translocase (flippase) Dnf1p; involved in translocation of phospholipids and alkylphosphocholine drugs across the plasma membrane; null mutant requires tryptophan due to mislocalization of tryptophan permease Tat2p
NSG2	-2.07	Protein involved in regulation of sterol biosynthesis; specifically stabilizes Hmg2p, one of two HMG-CoA isoenzymes that catalyze the rate-limiting step in sterol biosynthesis; homolog of mammalian INSIG proteins; NSG2 has a paralog, NSG1, that arose from the whole genome duplication
YEL033W	-2.08	Protein of unknown function; predicted metabolic role based on network analysis derived from ChIP experiments, a large-scale deletion study and localization of transcription factor binding sites; null mutant is sensitive to temperature oscillation in a cdc13-1 mutant
YJL202C	-2.08	Dubious open reading frame; unlikely to encode a functional protein, based on available experimental and comparative sequence data; overlaps 3' end of essential PRP21 gene encoding a subunit of the SF3a splicing factor complex
YDR344C	-2.09	Putative protein of unknown function; conserved among S. cerevisiae strains
PAN3	-2.09	Essential subunit of the Pan2p-Pan3p poly(A)-ribonuclease complex; poly (A) mRNA binding subunit which recruits mRNA to the complex; the Pan2p-Pan3p complex controls poly(A) tail length and regulates the stoichiometry and activity of postreplication repair complexes
OLI1	-2.09	F0-ATP synthase subunit c (ATPase-associated proteolipid); encoded on the mitochondrial genome; mutation confers oligomycin resistance; expression is specifically dependent on the nuclear genes AEP1 and AEP2
YHR162W	-2.09	Highly conserved subunit of the mitochondrial pyruvate carrier (MPC); expressed during growth on fermentable carbon sources, and heterodimerizes with Mpc1p to form the fermentative isoform of MPC; MPC localizes to the mitochondrial inner membrane and mediates pyruvate uptake; MPC2 paralog, MPC3, heterodimerizes with Mpc1p to form the respiratory MPC isoform
THI22	-2.10	Protein with similarity to hydroxymethylpyrimidine phosphate kinases; member of a gene family with THI20 and THI21; not required for thiamine biosynthesis; SWAT-GFP and mCherry fusion proteins localize to the endoplasmic reticulum and vacuole respectively
ATP8	-2.12	Subunit 8 of the F0 sector of mitochondrial F1F0 ATP synthase; encoded on the mitochondrial genome; ATP8 and ATP6 mRNAs are not translated in the absence of the F1 sector of ATPase
YCH1	-2.15	Phosphatase with sequence similarity to Mih1p; member of the Cdc25p subfamily of tyrosine phosphatases and Arr2p, an arsenate reductase, based on the presence of a rhodanese-homology domain; green fluorescent protein (GFP)-fusion protein localizes to both the cytoplasm and the nucleus
YBL100W-B	-2.15	Retrotransposon TYA Gag and TYB Pol genes; transcribed/translated as one unit; polyprotein is processed to make a nucleocapsid-like protein (Gag), reverse transcriptase (RT), protease (PR), and integrase (IN); similar to retroviral genes
SLM4	-2.16	Subunit of EGO/GSE complex; vacuolar/endosomal membrane-associated EGO/GSE complex regulates exit from rapamycin-induced growth arrest, stimulating microautophagy and sorting of Gap1p from endosome to plasma membrane; essential for integrity and function of EGO; targeted to vacuole via AP-3 pathway; gene exhibits synthetic genetic interaction with MSS4
YMR158C-A	-2.17	Protein of unknown function; may contain a lipid attachment site; localizes to cytosol, and to peroxisomes in oleate-growing cells; YMR158C-A is not an essential gene
SDH4	-2.17	Membrane anchor subunit of succinate dehydrogenase (SDH); involved in coupling the oxidation of succinate to the transfer of electrons to ubiquinone as part of the TCA cycle and the mitochondrial respiratory chain; has similarity to human SDH subunit D (SDHD), which is implicated in paraganglioma
ERP6	-2.24	Member of the p24 family involved in ER to Golgi transport; similar to Emp24p and Erv25p; the authentic, non-tagged protein is detected in highly purified mitochondria in high-throughput studies; ERP6 has a paralog, ERP1, that arose from the whole genome duplication
NUP85	-2.25	Subunit of the Nup84p subcomplex of the nuclear pore complex (NPC); contributes to nucleocytoplasmic transport and NPC biogenesis and is involved in establishment of a normal nucleocytoplasmic concentration gradient of the GTPase Gsp1p; also plays roles in several processes that may require localization of genes or chromosomes at the nuclear periphery, including double-strand break repair, transcription and chromatin silencing; homologous to human NUP85 aka NUP75
YSA1	-2.27	Nudix hydrolase family member with ADP-ribose pyrophosphatase activity; shown to metabolize O-acetyl-ADP-ribose to AMP and acetylated ribose 5'-phosphate
YPL261C	-2.29	Putative protein of unknown function; conserved among S. cerevisiae strains; YPL261C is not an essential gene; partially overlaps verified ORF YPL260W
YBL100W-B	-2.32	Retrotransposon TYA Gag and TYB Pol genes; transcribed/translated as one unit; polyprotein is processed to make a nucleocapsid-like protein (Gag), reverse transcriptase (RT), protease (PR), and integrase (IN); similar to retroviral genes
FLC3	-2.37	Putative FAD transporter, similar to Flc1p and Flc2p; localized to the ER; FLC3 has a paralog, FLC1, that arose from the whole genome duplication
YBL100W-B	-2.37	Retrotransposon TYA Gag and TYB Pol genes; transcribed/translated as one unit; polyprotein is processed to make a nucleocapsid-like protein (Gag), reverse transcriptase (RT), protease (PR), and integrase (IN); similar to retroviral genes
YBL100W-B	-2.38	Retrotransposon TYA Gag and TYB Pol genes; transcribed/translated as one unit; polyprotein is processed to make a nucleocapsid-like protein (Gag), reverse transcriptase (RT), protease (PR), and integrase (IN); similar to retroviral genes
NMA111	-2.39	Serine protease and general molecular chaperone; cleaves Roq1p, which modifies the substrate specificity of the Ubr1p Ub-ligase, promoting the stress-induced homeostatically-regulated protein degradation (SHRED) of misfolded and native ER-membrane and cytosolic proteins; chaperone activity involved in the heat stress response; promotes apoptosis through proteolysis of Bir1p; role in lipid homeostasis; mammalian Omi/HtrA2 serine protease family member
SMY2	-2.41	GYF domain protein; involved in COPII vesicle formation; regulates Cdc48p function in transcription stress response; interacts with the Sec23p/Sec24p subcomplex; overexpression suppresses the temperature sensitivity of a myo2 mutant; homologous to human GIGYF1 and GIGYF2; similar to S. pombe Mpd2; SMY2 has a paralog, SYH1, that arose from the whole genome duplication
YBL100W-B	-2.41	Retrotransposon TYA Gag and TYB Pol genes; transcribed/translated as one unit; polyprotein is processed to make a nucleocapsid-like protein (Gag), reverse transcriptase (RT), protease (PR), and integrase (IN); similar to retroviral genes
GPA2	-2.44	Nucleotide binding alpha subunit of the heterotrimeric G protein; interacts with the receptor Gpr1p, has signaling role in response to nutrients; required for the recruitment of Ras-GTP at the plasma membrane and in the nucleus
YCR018C-A	-2.46	Dubious open reading frame; unlikely to encode a functional protein, based on available experimental and comparative sequence data; completely overlaps the Ty1 long terminal repeat, YPRWdelta12
AAD15	-2.49	Putative aryl-alcohol dehydrogenase; similar to P. chrysosporium aryl-alcohol dehydrogenase; mutational analysis has not yet revealed a physiological role; AAD15 has a paralog, AAD3, that arose from a segmental duplication; members of the AAD gene family comprise three pairs (AAD3 + AAD15, AAD6/AAD16 + AAD4, AAD10 + AAD14) whose two genes are more related to one another than to other members of the family
JJJ3	-2.53	Iron-binding protein; involved in targeting cytoplasmic SRP-independent proteins to the ER; contains a CSL Zn finger and a DnaJ-domain; involved in diphthamide biosynthesis; ortholog human Dph4
YNL284C-A	-2.59	Retrotransposon TYA Gag gene co-transcribed with TYB Pol; translated as TYA or TYA-TYB polyprotein; Gag is a nucleocapsid protein that is the structural constituent of virus-like particles (VLPs); similar to retroviral Gag
YDR316W-B	-2.63	Retrotransposon TYA Gag and TYB Pol genes; transcribed/translated as one unit; polyprotein is processed to make a nucleocapsid-like protein (Gag), reverse transcriptase (RT), protease (PR), and integrase (IN); similar to retroviral genes; YHR214C-B has a paralog, YAR070C, that arose from a segmental duplication
YAR010C	-2.79	Retrotransposon TYA Gag gene co-transcribed with TYB Pol; translated as TYA or TYA-TYB polyprotein; Gag is a nucleocapsid protein that is the structural constituent of virus-like particles (VLPs); similar to retroviral Gag
CUP1-2	-2.89	Metallothionein; binds copper and mediates resistance to high concentrations of copper and cadmium; locus is variably amplified in different strains, with two copies, CUP1-1 and CUP1-2, in the genomic sequence reference strain S288C; CUP1-2 has a paralog, CUP1-1, that arose from a segmental duplication
YBL100W-B	-2.89	Retrotransposon TYA Gag and TYB Pol genes; transcribed/translated as one unit; polyprotein is processed to make a nucleocapsid-like protein (Gag), reverse transcriptase (RT), protease (PR), and integrase (IN); similar to retroviral genes
YNL275W	-2.99	Boron efflux transporter of the plasma membrane; binds HCO3-, I-, Br-, NO3- and Cl-; has similarity to the characterized boron efflux transporter A. thaliana BOR1
YAR009C	-3.07	Retrotransposon TYA Gag and TYB Pol genes; transcribed/translated as one unit; polyprotein is processed to make a nucleocapsid-like protein (Gag), reverse transcriptase (RT), protease (PR), and integrase (IN); similar to retroviral genes
PEX22	-3.57	Putative peroxisomal membrane protein; required for import of peroxisomal proteins; functionally complements a Pichia pastoris pex22 mutation
