# Supplementary material for: The Lack of a COPII Cargo Receptor Erv14 Impacts Physiological Functions of the Vacuole in Saccharomyces cerevisiae
Source: Traffic. 2026 Apr 23;27:e70035. doi: 10.1111/tra.70035 (PMC13106738; doi:10.1111/tra.70035)
Supplement: Supplementary file 4 — Data S1: Supporting Information and Methods Table 1. Oligonucleotides used for gene cloning. [file TRA-27-e70035-s004.pdf]

Gene	*Forward primer* **5'-3'**	*Reverse primer* **5'-3'**
ScHXT3	GTACATTATAAAAAAAAATCCTGAACTTAGCTAGA TATT ATGAATTCAACTCCAGATTTAA	TAAAGCTCCGGAGCTTGCATGCCTGCAGGTCGACT CT TTTCTTGCCGAACATTTTCTTGT
ScHXT5	GTACATTATAAAAAAAAATCCTGAACTTAGCTAGA TATT ATGTCGGAACTTGAAAACGCTC	TAAAGCTCCGGAGCTTGCATGCCTGCAGGTCGACT CT TTTTTCTTTAGTGAACATCCTT
ScVPH1	GTACATTATAAAAAAAAATCCTGAACTTAGCTAGA TATT ATGGCAGAGAAGGAGGAAGCGA	TAAAGCTCCGGAGCTTGCATGCCTGCAGGTCGACT CT GCTTGAAGCGGAAGAGCTTGCAC

Supplementary Material and Methods Table 1. Oligonucleotides used for gene cloning.
