# Supplementary material for: The Lack of a COPII Cargo Receptor Erv14 Impacts Physiological Functions of the Vacuole in Saccharomyces cerevisiae
Source: Traffic. 2026 Apr 23;27:e70035. doi: 10.1111/tra.70035 (PMC13106738; doi:10.1111/tra.70035)
Supplement: Supplementary file 5 — Data S2: Supporting Information and Methods Table 2: List of interactions used to build the global interaction network of the erv14Δ strain. [file TRA-27-e70035-s001.pdf]

Supplementary Material and Methods Table 2. List of interactions to build the global interaction network that change their transcription rate in the *erv14Δ* null mutant.

Gene in transcriptome	Interaction	interacting with
VPS8	genetic	VAM7
VPS8	genetic	VAM7
VPS8	physical	VAM7
CCR4	physical	VAM7
KIN3	genetic	VAM7
TKL2	genetic	VAM7
YSW1	physical	VAM7
SPP381	genetic	VAM7
SPP381	genetic	VAM7
SPP381	genetic	VAM7
BSD2	genetic	VAM7
BSD2	genetic	VAM7
ENT1	genetic	VAM7
RGP1	genetic	VAM7
RGP1	genetic	VAM7
COX20	genetic	VAM7
MMS21	genetic	VAM7

OTU1	genetic	VAM7
SPR3	genetic	VAM7
SPR3	genetic	VAM7
COG2	genetic	VAM7
TFG1	genetic	VAM7
TAF1	genetic	VAM7
YOR1	genetic	VAM7
SLT2	genetic	VAM7
CPR2	genetic	VAM7
PAN5	genetic	VAM7
BZZ1	genetic	VAM7
BZZ1	genetic	VAM7
BZZ1	genetic	VAM7
ARP1	genetic	VAM7
RPS4B	genetic	VAM7
TED1	genetic	VAM7
GVP36	genetic	VAM7
GVP36	genetic	VAM7
GYP6	genetic	VAM7

GYP6	genetic	VAM7
JEM1	genetic	VAM7
JEM1	genetic	VAM7
JEM1	genetic	VAM7
KHA1	genetic	VAM7
BCK1	genetic	VAM7
MNN11	genetic	VAM7
MNN11	genetic	VAM7
MNN11	genetic	VAM7
MNN11	genetic	VAM7
NTA1	genetic	VAM7
CCE1	genetic	VAM7
RRP14	genetic	VAM7
ESL2	genetic	VAM7
MMM1	genetic	VAM7
UBR2	genetic	VAM7
RPL22A	genetic	VAM7
ARP6	genetic	VAM7
SSP120	genetic	VAM7

CDC3	physical	VAM7
MVP1	genetic	VAM7
MVP1	genetic	VAM7
MVP1	genetic	VAM7
MVP1	genetic	VAM7
MSN2	genetic	VAM7
PDS5	genetic	VAM7
SIW14	genetic	VAM7
NOP2	genetic	VAM7
NOP2	genetic	VAM7
RPL16B	genetic	VAM7
SIN4	genetic	VAM7
SIN4	genetic	VAM7
BRE5	genetic	VAM7
BRE5	genetic	VAM7
BRE5	genetic	VAM7
RTS1	genetic	VAM7
HMS1	genetic	VAM7
RKI1	genetic	VAM7

IDH2	genetic	VAM7
VPS30	genetic	VAM7
ASA1	genetic	VAM7
BSP1	physical	VAM7
